# Supplementary material for: The Walloon farmers position differently their ideal dairy production system between a global-based intensive and a local-based extensive model of farm
Source: PLoS One. 2020 Dec 3;15(12):e0223346. doi: 10.1371/journal.pone.0223346 (PMC7714150; doi:10.1371/journal.pone.0223346)
Supplement: S1 Appendix — (DOCX) [file pone.0223346.s001.docx]

S1 Appendix : Questions of the survey mobilized in the study

- : Unique answer
- : Multiple answers allowed

| Question | Answer |
| --- | --- |
| In which province is located your farm? | - Walloon Brabant - Liège - Namur - Hainaut - Luxembourg |
| What is your age? | - < 25 years - 25-34 years - 35-44 years - 45-54 years - 55-64 years - > 65 years |
| What is the composition of the workforce of your farm?   - Chief operating officer or associates | Number of persons = |
| What is the distribution of the workforce regarding their status?   - Employees | Number of persons = |
| Do you subscribe to an agricultural replacement service? | - Yes - No |
| What are the size of the followings area and productions?   - Total agricultural area - Meadow - Corn silage | For each proposition : number of hectares = |
| What is the importance of the dairy production in terms of working time against all the agricultural activities of your farm? | - Dairy production is the unique activity - Dairy production is the preponderant activity (principal or at least equally to the other activities) - Dairy production is a secondary activity (less important than the other activities) |
| What is the destination of your milk production? | - Sale to a dairy factory - Processing and/or direct sale - Other : |
| What is your milk delivery quota? | Number of liters = |
| What is the number of milked cows on your farm? | Number of cows = |
| What is the main breed of your cows? | - Holstein - Red-Holstein - Red dual-purpose - Belgian blue dual purpose - Montbéliarde - Normande - Jersey - Brown Swiss - Others and crossings : |
| Are there milked cows which come from another breed? | - No - Holstein - Red-Holstein - Red dual-purpose - Belgian blue dual purpose - Montbéliarde - Normande - Jersey - Brown Swiss - Others and crossings |
| Are there other animal production on the farm? | - Yes - No |
| Are there other activities on the farm?   - Direct sale (other than dairy) | Yes or no |
| What are the most important information source of your farm?   - Agricultural press | Yes or no |
| Since 2009, have you modified the milk quantity produced on your farm? | - Yes, I have increased the quantity of milk produced - Yes, I have decreased the quantity of milk produced - No, I have not modified the quantity of milk produced |
| Of which milk quantity have you increased since 2009? | Number of litres = |
| Of which milk quantity have you decreased since 2009? | Number of litres = |
| In the next five years, which evolution of milk quantity produced on your farm are considering? | - I am considering an increase of the milk quantity produced - I am considering a decrease of the milk quantity produced - I am considering to produce an equivalent milk quantity to what I am producing currently - I am considering the stop of the dairy activity |
| Currently, what is the degree of arduousness of the followings constraints on your dairy production?   - Workforce, workload | - Very high - High - Moderate - Low - Zero |
| What is the reason(s) which ask you to maintain your dairy production constant or to decrease it?   - Self-sufficiency on the farm | Yes or no |
| Concerning the other activities on your farm, which evolutions are you considering to better adapt yourself to the future changes (within five years)?   - Increase of the activities without investment | Yes or no |
| Since 2009, have you increased the agricultural area of your farm? | - Yes - No |
| Since 2009, have you increased one or more investments (other than agricultural area)? | - Yes, I realized investments (replacement or development/enlargement) - No, no investment, nor replacement, neither development/enlargement |
| Which investments did you realized since 2009 (other than agricultural area)?   - Milking robot - Agricultural equipment for a better technicality (automatic concentrate feeder, computerized system,…) | - Replacement - Development and/or enlargement |
| In the next five years, do you want to increase the agricultural area of your farm? | - Yes - No |
| Which investments do you want to realize in the next five years (other than agricultural area)?   - Milking robot - Agricultural equipment for a better technicality (automatic concentrate feeder, computerized system,…) | - Replacement - Development and/or enlargement |
| According to you, what are the main advantages related to the association, the regrouping or a society?   - Benefitting of the fiscal and administrative aspects - Working in cooperation, develop a joint project - Better marketing his product | For each proposition : yes or no |
| Which size of dairy factory is ideal? | - Not an important criterion - Dairy factory of small size - Dairy factory of middle size - Dairy factory of big size |
| Against the imposition of production limits to the producers, which kind of dairy factory would be ideal? | - Not an important criterion - A dairy factory which imposes a limit - A dairy factory which does not impose a limit |
| In the context of a farm like yours, which activities could be developed if sustained (even if it necessits investments)?   - Transformation of dairy products - Transformation of other than dairy products - HORECA, tourism, teaching activities - None | For each proposition : yes or no |
| According to you, what are the main advantages of the transformation and the diversification?   - Increase of the added value in farms - Link between producers and consumers - Conservation of farms in the region - Financial, decisional and technical autonomy - None | For each proposition : yes or no |
| According to you, what are the main limits of the transformation and the diversification?   - Consumer loyalty - Size of investments - Regulatory constraints (hygiene, etc.) - None | For each proposition : yes or no |
| In the context of your farm, are you concerned by the diversification of the agricultural activities (already realized or to realize)? | - Yes - No |
| Do you agree with the following sentences?  “The quota removal will give more flexibility to producers concerning their production quantities” | - Completely agree (5) - Somewhat agree (4) - Somewhat disagree (2) - Strongly disagree (1) - No opinion (3)   (x) : number on the scale of level of agreement |
| Do you agree with the following sentences?  “The contracts will lead to a system of production more integrated to dairy factories.” | - Completely agree (5) - Somewhat agree (4) - Somewhat disagree (2) - Strongly disagree (1) - No opinion (3)   (x) : number on the scale of level of agreement |
| To decrease the effects of the crisis (2009 or 2012), in a general way, did you pursue to produce… | - More milk - So much milk - Less milk |
| The crisis (2009 or 2012) did it decide you to modify deeply at long term your way to produce milk? | - Yes - No |
| Do you agree with the following assertions?  Your agricultural activity plays an important role concerning   - The biodiversity - The rurality of villages - The plantation and the maintenance of hedges - The conservation of permanent grasslands | For each proposition :   - Completely agree (5) - Somewhat agree (4) - Somewhat disagree (2) - Strongly disagree (1) - No opinion (3)   (x) : number on the scale of level of agreement |
| Do you agree with the following assertions?  For your farm, the law represents serious constraints in terms of   - Livestock manure application area | - Completely agree (5) - Somewhat agree (4) - Somewhat disagree (2) - Strongly disagree (1) - No opinion (3)   (x) : number on the scale of level of agreement |
| Among the following practices, what are those that you execute regularly to optimize the production?   - Measurement of the grass height - Forage mixture with leguminous plants - Field notebook (paper or computerized) | For each proposition : yes or no |
| In a general way, which strategies do you adopt to anticipate or to limit the negative impacts of the rare climatic hazards (droughts, storms, hail, etc.)?   - Increase of the concentrate distribution - Decrease of the herd | For each proposition : yes or no |
| Without taking into account your current farm, what is, according to you, the ideal future farm to ensure a revenue to the farmer?  Express your opinion on each groups of the followings criteria. | - Intensive - Extensive - No opinion |
|  | - Specialized - Diversified - No opinion |
|  | - Strongly based on new technologies - Lowly based on new technologies - No opinion |
|  | - Based on familial workforce - Based on salaried no-familial workforce - No opinion |
|  | - Managed by an independent farmer - Managed by an association of farmers (or society, regrouping, etc.) - No opinion |
|  | - Production for the local market - Production for the global market - No opinion |
|  | - Standard milk production - Quality differentiated milk production - No opinion |
| To support the implementation of the ideal future farm, what is the importance of the topics of trainings, information or studies?   - Animal selection - Animal feeding - Plant selection - Finance and management - Administrative - Legal framework - Transformation and diversification | For each proposition : yes or no |
| What is/are the kind(s) of trainings, information or studies appropriate to answer to the needs of the ideal future farm?   - Implementation of a network of pilot farms - Specific advices, consultancy company - Study days on farm | For each proposition : yes or no |
| What is/are the most important organization(s) to support the development of the ideal farms of tomorrow?   - Commercial companies - Associate, non-market sector | For each proposition : yes or no |
| What is your opinion on the following assertions?  For the producers, it is easy to answer to the society’s expectations which concerns :   - Environmentally friendly agricultural practices (regarding water, soil, air, biodiversity) - Landscape and territory maintenance | For each proposition :   - Completely agree (5) - Somewhat agree (4) - Somewhat disagree (2) - Strongly disagree (1) - No opinion (3)   (x) : number on the scale of level of agreement |
| What is your opinion on the following assertions?   - Answer to the society’s expectations is important for the producers from a financial point of view (valorization of the production, sales price, subsidies, etc.) | For each proposition :   - Completely agree (5) - Somewhat agree (4) - Somewhat disagree (2) - Strongly disagree (1) - No opinion (3)   (x) : number on the scale of level of agreement |
| Among the following stress sources, qualify their degree of arduousness according to you.   - Workload - Economic uncertainties on the inputs price (feeding, etc.) | For each proposition :   - Not arduous - Arduous - Highly arduous |
| To limit the stress sources, which solutions do you consider or do you wish to implement?  For help for the workload and the administrative aspects   - Worker engagement - Mechanisation and robotisation   For help for the financial aspects   - Request for advice   For help for technical choices   - Formation and study day - Producers technical groups | For each proposition :   - Already implemented - To implement in the future - Not interested |
