# Supplementary material for: The Walloon farmers position differently their ideal dairy production system between a global-based intensive and a local-based extensive model of farm
Source: PLoS One. 2020 Dec 3;15(12):e0223346. doi: 10.1371/journal.pone.0223346 (PMC7714150; doi:10.1371/journal.pone.0223346)
Supplement: Attachment — Submitted filename: PONE-D-19-26278_reviewer.pdf [file pone.0223346.s002.pdf]

PLOS ONE

How do dairy farmers wish their future farm?

--Manuscript Draft--

**Manuscript Number:**	PONE-D-19-26278
**Article Type:**	Research Article
**Full Title:**	How do dairy farmers wish their future farm?
**Short Title:**	Ideal future dairy farms to ensure revenue
**Corresponding Author:**	Anne-Catherine Dalcq<br>Universite de Liege Gembloux Agro-Bio Tech<br>Gembloux, BELGIUM
**Keywords:**	Dairy farming systems; future; producers; reasons; Environment; climatic hazard; formation
**Abstract:**	Dairy farming systems are evolving. This study presents dairy producers' perceptions of their ideal future farm (IFF) to ensure revenue and attempts to determine the reasons for this choice, the environmental aspects related to this choice, the proximity between the current farm and the IFF and the requirements for reaching this IFF. Just before the end of the European milk quota, a total of 245 dairy producers answered a survey about the characteristics of their IFF and other socio-environmental-economic information. A multiple correspondence analysis (MCA) was carried out using seven characteristics of IFF (intensive vs. extensive, specialised vs. diversified, strongly vs. weakly based on new technologies, managed by a group of managers vs. an independent farmer, employed vs. familial workforce, local vs. global market, standard vs. quality-differentiated production). Based on the main contributors to the second dimension of MCA, this axis was defined as an IFF gradient between the local-based extensive (LBE) producers (26%) and the global-based intensive (GBI) producers (46%). The differences of IFF gradient between modalities of qualitative variables were estimated using generalised linear models. Pearson correlations were calculated between the scores on the IFF gradient and quantitative variables. Finally, frequencies for IFF characteristic and the corresponding characteristic for the current situation were calculated to determine the percentages of "unhappy" producers. Some reasons for the choice of IFF by the producers have been highlighted in this study. Environmental initiatives were more valued by LBE than GBI producers. Low similarity was observed between the current farm situation of the respondents and their IFF choice. LBE and GBI producers differed significantly regarding domains of formation (technical and bureaucratic vs transformation and diversification respectively) and paths of formation (non-market vs. market respectively). Two kinds of farming systems were considered by dairy producers and some socioeconomic and environmental components differed between them.
**Order of Authors:**	Anne-Catherine Dalcq
	Thomas Dogot
	Yves Beckers
	Yves Brostaux
	Eric Froidmont
	Frédéric Vanwindekens
	Hélène Soyeurt
**Additional Information:**	
**Question**	**Response**
**Financial Disclosure**<br><br>Enter a financial disclosure statement that describes the sources of funding for the	The author(s) received no specific funding for this work.

Powered by Editorial Manager® and ProduXion Manager® from Aries Systems Corporation

work included in this submission. Review the submission guidelines for detailed requirements. View published research articles from *PLOS ONE* for specific examples.<br><br>This statement is required for submission and **will appear in the published article** if the submission is accepted. Please make sure it is accurate.<br><br>**Unfunded studies**<br>Enter: *The author(s) received no specific funding for this work.*<br><br>**Funded studies**<br>Enter a statement with the following details:<br>• Initials of the authors who received each award<br>• Grant numbers awarded to each author<br>• The full name of each funder<br>• URL of each funder website<br>• Did the sponsors or funders play any role in the study design, data collection and analysis, decision to publish, or preparation of the manuscript?<br>• **NO** - Include this sentence at the end of your statement: *The funders had no role in study design, data collection and analysis, decision to publish, or preparation of the manuscript.*<br>• **YES** - Specify the role(s) played.<br><br>* typeset	
**Competing Interests**<br><br>Use the instructions below to enter a competing interest statement for this submission. On behalf of all authors, disclose any competing interests that could be perceived to bias this work—acknowledging all financial support and any other relevant financial or non-financial competing interests.<br><br>This statement **will appear in the published article** if the submission is accepted. Please make sure it is accurate. View published research articles from *PLOS ONE* for specific examples.	The authors have declared that no competing interests exist.

Powered by Editorial Manager® and ProduXion Manager® from Aries Systems Corporation

**NO authors have competing interests**<br><br>Enter: *The authors have declared that no competing interests exist.*<br><br>**Authors with competing interests**<br><br>Enter competing interest details beginning with this statement:<br><br>*I have read the journal's policy and the authors of this manuscript have the following competing interests: [insert competing interests here]*<br><br>* typeset	
**Ethics Statement**<br><br>Enter an ethics statement for this submission. This statement is required if the study involved:<br><br>• Human participants<br>• Human specimens or tissue<br>• Vertebrate animals or cephalopods<br>• Vertebrate embryos or tissues<br>• Field research<br><br>Write "N/A" if the submission does not require an ethics statement.<br><br>General guidance is provided below. Consult the submission guidelines for detailed instructions. **Make sure that all information entered here is included in the Methods section of the manuscript.**	A remark has been done the 23th September 2019 by the Editorial board concerning the ethics statement of my manuscript. You informed me that there were human participants in my study. Indeed dairy producers answered to a survey (electronically and anonymously). I checked your guidelines regarding human participants. It explains the requirement to follow the Declaration of Helsinki. This declaration concerns medical research with humans participation. My study only used answers of persons to a survey. What do you need concerning my manuscript to have a fulfilling submission? I remain at your disposal for further information.

Powered by Editorial Manager® and ProduXion Manager® from Aries Systems Corporation

**Format for specific study types**<br><br>**Human Subject Research (involving human participants and/or tissue)**<br>• Give the name of the institutional review board or ethics committee that approved the study<br>• Include the approval number and/or a statement indicating approval of this research<br>• Indicate the form of consent obtained (written/oral) or the reason that consent was not obtained (e.g. the data were analyzed anonymously)<br><br>**Animal Research (involving vertebrate animals, embryos or tissues)**<br>• Provide the name of the Institutional Animal Care and Use Committee (IACUC) or other relevant ethics board that reviewed the study protocol, and indicate whether they approved this research or granted a formal waiver of ethical approval<br>• Include an approval number if one was obtained<br>• If the study involved *non-human primates*, add *additional details* about animal welfare and steps taken to ameliorate suffering<br>• If anesthesia, euthanasia, or any kind of animal sacrifice is part of the study, include briefly which substances and/or methods were applied<br><br>**Field Research**<br><br>Include the following details if this study involves the collection of plant, animal, or other materials from a natural setting:<br>• Field permit number<br>• Name of the institution or relevant body that granted permission	
**Data Availability**<br><br>Authors are required to make all data underlying the findings described fully available, without restriction, and from the time of publication. PLOS allows rare exceptions to address legal and ethical concerns. See the PLOS Data Policy and FAQ for detailed information.	No - some restrictions will apply

A Data Availability Statement describing where the data can be found is required at submission. Your answers to this question constitute the Data Availability Statement and **will be published in the article**, if accepted.<br><br>**Important:** Stating 'data available on request from the author' is not sufficient. If your data are only available upon request, select 'No' for the first question and explain your exceptional situation in the text box.<br><br>Do the authors confirm that all data underlying the findings described in their manuscript are fully available without restriction?	
**Describe where the data may be found in full sentences. If you are copying our sample text, replace any instances of XXX with the appropriate details.**<br><br>• If the data are **held or will be held in a public repository**, include URLs, accession numbers or DOIs. If this information will only be available after acceptance, indicate this by ticking the box below. For example: *All XXX files are available from the XXX database (accession number(s) XXX, XXX.*).<br>• If the data are all contained **within the manuscript and/or Supporting Information files**, enter the following: *All relevant data are within the manuscript and its Supporting Information files.*<br>• If neither of these applies but you are able to provide **details of access elsewhere**, with or without limitations, please do so. For example:<br><br>*Data cannot be shared publicly because of [XXX]. Data are available from the XXX Institutional Data Access / Ethics Committee (contact via XXX) for researchers who meet the criteria for access to confidential data.*<br><br>*The data underlying the results presented in the study are available from (include the name of the third party*	Data were collected by the Committee of 'Carrefour des Productions Animales', a committee of stakeholders of animal sector in the Walloon Region of Belgium (research center, agricultural unions, university). A request can be made to them to get data.

Powered by Editorial Manager® and ProduXion Manager® from Aries Systems Corporation

and contact information or URL).<br>• This text is appropriate if the data are owned by a third party and authors do not have permission to share the data.<br><br>* typeset	
Additional data availability information:	

Manuscript
Click here to access/download;Manuscript;Ideal future dairy farms to ensure revenue AC Dalcq.docx

How do dairy farmers wish their future farm?

Abstract 2
Introduction 4
Materials and methods 7
Survey and IFF typology 7
Characterisation of IFF choice 8
Results and discussion 11
Contrasted opinions of Walloon dairy farmers about the ideal future farm 11
Dualisation of ideal future farm aspirations 12
Relationships between ideal future farms and reasons, environmental considerations and formations 16
Reasons 17
Consideration of environmental aspects 27
Current situation vs. ideal future farm 28
Needs 29
Conclusions 32
Acknowledgments 32
References 33

Anne-Catherine Dalcq[1]*, Thomas Dogot[1], Yves Beckers[1], Yves Brostaux[1], Eric Froidmont[2], Frédéric Vanwindekens[2], Hélène Soyeurt[1]

[1] University of Liège, Gembloux Agro-Bio Tech, TERRA Teaching and Research Centre, Passage des Déportés 2, 5030 Gembloux, Belgium

[2] Walloon Research Centre, Rue du Liroux, 8, 5030 Gembloux, Belgium

*Corresponding author

E-mail : anne-catherine.dalcq@uliege.be (ACD)

Ideal future dairy farms to ensure revenue

Abstract

Dairy farming systems are evolving. This study presents dairy producers' perceptions of their ideal future farm (**IFF**) to ensure revenue and attempts to determine the reasons for this choice, the environmental aspects related to this choice, the proximity between the current farm and the IFF and the requirements for reaching this IFF. Just before the end of the European milk quota, a total of 245 dairy producers answered a survey about the characteristics of their IFF and other socio-environmental-economic information. A multiple correspondence analysis (**MCA**) was carried out using seven characteristics of IFF (intensive *vs.* extensive, specialised *vs.* diversified, strongly *vs.* weakly based on new technologies, managed by a group of managers *vs.* an independent farmer, employed *vs.* familial workforce, local *vs.* global market, standard *vs.* quality-differentiated production). Based on the main contributors to the second dimension of MCA, this axis was defined as an IFF gradient between the local-based

extensive (**LBE**) producers (26%) and the global-based intensive (**GBI**) producers (46%). The differences of IFF gradient between modalities of qualitative variables were estimated using generalised linear models. Pearson correlations were calculated between the scores on the IFF gradient and quantitative variables. Finally, frequencies for IFF characteristic and the corresponding characteristic for the current situation were calculated to determine the percentages of “unhappy” producers. Some reasons for the choice of IFF by the producers have been highlighted in this study. Environmental initiatives were more valued by LBE than GBI producers. Low similarity was observed between the current farm situation of the respondents and their IFF choice. LBE and GBI producers differed significantly regarding domains of formation (technical and bureaucratic vs transformation and diversification respectively) and paths of formation (non-market vs. market respectively). Two kinds of farming systems were considered by dairy producers and some socioeconomic and environmental components differed between them.

Introduction

Food is a basic need. Working to provide food for themselves and their family was the task of everyone at the dawn of humanity. The progressive organisation of society has led to the appearance of “producers” who are responsible for producing food for more than just themselves and their family. Since World War II, public policies have been set up to increase food production [1]. These policies impacted the development of producers and their farms in the European Union. In the southern part of Belgium, the mean number of cows and the mean agricultural area per producer increased between 1980 and 2017 from 20 to 66 heads and from 25 to 71 hectares, respectively [2].

Producers are now facing great challenges to stay profitable. The price of the inputs (e.g., buildings, agricultural machinery, installations, feeding, veterinary care) of dairy production (**DP**) are increasing while the milk price shows great variability and its inflation is not similar to that observed for the inputs [3, 4]. Moreover, the European Union has decreased financial support to farmers [5]. On 1st April 2015, the European Union removed the quota system which had managed the supply of DP [6]. This led to greater milk price volatility. Additionally, sanitary crises such as mad cow disease (bovine spongiform encephalopathy (BSE)) and the dioxine crisis, among others, have shocked consumers and led to new rules and regulations at European level and led to the creation of food security agencies in its countries. Moreover, these episodes modified consumers’ behaviours regarding their food purchases, they asked for more transparency and directed themselves towards organic food or local chains [7]. Besides the economic view, the impacts of farming on the environment have been noted and policies have been set up in the Common Agricultural Policy to solve these problems [4, 8].

In this context, the question often asked is what the future of dairy farming entails, how to remain profitable and more generally sustainable. Several authors have studied the evolution of dairy farming and the present dairy systems, finding trends that exist in the sector [3, 4, 9]. For instance, the project Mouve, funded by the French National Research Agency, studied the evolution of dairy farming systems in 6 dairy basins around the world. Their results gathered the publications of Napoleone *et al.* (2014) [9] and Havet *et al.* (2015) [3]. Moreover, authors have studied the future paths of development considered by dairy producers [10-13]. These studies were realised on the basis of data from 2001 to the beginning of 2013. These studies explored some reasons for these choices [10-14].

This study is innovative as it asks a different question to the other studies: what is the ideal future farm (**IFF**) to ensure revenue. Moreover, respondent producers were asked not to take into account their current farm. The data collection was conducted at the end of 2014 and the beginning of 2015. This was a particular context, just before the quota removal, when producers had this new perspective in mind and following two important milk crises associating low milk price due to a deregulation of EU milk production in 2009 and an increase of the cost of inputs in 2012. This research studied unprecedented reasons for this choice compared to what is present in the literature, to our knowledge, such as past events of the farms. Moreover, the present study explored the environmental and formational aspects linked to this IFF vision. The environmental aspect is of high importance at a time of increasing awareness of the impacts of agriculture and breeding on the environment. The formation aspect was studied to orientate university and other stakeholders of breeding improvement towards the domains needed by dairy producers. A comparison between the current farm and the IFF of the respondent was realised, and permitted the difference between the reality

and the aspiration of the producers to be studied. More specifically, the goals of this study are to describe the perception of dairy producers about their ideal future dairy farming systems, and to quantify the proportion of producers desiring different dairy systems. By gathering different kinds of information, of which some are novel or a rarely present in the literature, this study also analyses the relationships between the dairy farming system desired by the producers and the reason(s) for this choice, their considerations about the environment and their needs to reach their goal of formation. Finally, we have ended this study by measuring the proximity between their current farming system and their IFF.

Materials and methods

All editing and statistical analyses were carried out using SAS software (version 9.4., SAS Inst. Inc., Cary, NC, USA).

Survey and IFF typology

A total of 245 Walloon dairy producers answered a survey between November 2014 and January 2015, the period just before the quota removal (1st April 2015). Dairy producers answered questions at a time when they had this new perspective in mind. The sample set represented 6.1% of the dairy producers in Wallonia (about 4000 dairy producers in 2015 and 3500 in 2017 [2]). The density of dairy farms throughout Wallonia was well represented in the sample, with a higher answer rate in the provinces more populated with dairy farms. More answers were obtained in the east part of Belgium, where a higher density of dairy farms exists due to the grazing landscape that is particularly suitable for dairy production. Wallonia is a highly heterogeneous region with regard to soil and geological characteristics [15].

Dairy producers of the survey declared a mean of 79 cows and 86 hectares. Dairy production was the unique activity for 33% of them.

The entire survey was composed of 127 questions where the answers were decomposed into 498 qualitative and 44 quantitative variables. Concerning the perception of the IFF desired by the dairy producers to ensure revenue, 7 questions dealt with the following aspects: intensive *vs.* extensive production; specialised *vs.* diversified activity (or activities); farming strongly *vs.* weakly based on new technologies; farm managed by an independent farmer *vs*. a group of managers; family *vs*. employed workforce; providing production for local *vs*. global markets; and providing standard *vs*. differentiated quality production. The modality "No opinion" was

available for each IFF question. Counts were calculated for all modalities of these seven questions.

To study the relationships between all modalities derived from the seven questions asked, a multiple correspondence analysis (**MCA**) was carried out as the variables were qualitative. For MCA, the eigenvalue of the dimensions generated, named principal inertia, is a biased measure of the part of information presented by a dimension [16]. Corrected inertia rates were calculated, as described by Benzécri (1979) [17], to quantify the correct part of information of a dimension.

Classes were established to study the distribution of producers along the dimensions of MCA. The interval between the 1% percentile and the 99% percentile of each dimension was divided equally into five classes. Then, the individuals per class were counted.

To exclude a group of producers with particular characteristics if necessary, cluster analysis was used on the scores of the individuals on the dimension of MCA, using the PROC CLUSTER procedure with the WARD method option.

Characterisation of IFF choice

To describe the dairy producers in terms of their IFF, the scores on MCA dimensions were studied as a function of other variables extracted from the survey and distributed within several themes. These were the effect of past crises, problems encountered by the farmer, production factors, age of the farmer, breed of the cow, diversification of activities and alternative valorisation, regrouping between producers, consideration of mechanisation and robotisation on the farm, the reaction of the farmer to external factors, the considerations of farmers about environmental aspects, climatic hazard, ways to reach the ideal formation and field of formation. For qualitative variables, the

scores of MCA dimensions were modelled using these variables as a fixed effect in a generalised linear model. Least squares means were estimated for the two-by-two comparisons using the Tukey test. The level of significance of those differences was assessed based on the *P*-value of the test. For quantitative variables, Pearson correlation coefficients were calculated between the scores of MCA dimensions and these variables. Their corresponding *P*-values were estimated to observe if the correlation values were significantly different from 0.

To observe if dairy producers presented the farming characteristics considered as ideal at the moment of survey, absolute frequencies were calculated as a function of each ideal future farm characteristic and the corresponding characteristic for the current situation (Table 1). Moreover, the percentage of “unhappy” producers was calculated as the ratio between the producers not currently in the situation that they consider as ideal and the total number of producers.

Table 1. Absolute frequency of producers as a function of their answer to the ideal future farm characteristic and the corresponding characteristic for the current situation and percentage of "unhappy" producers (i.e., percentage of producers not currently in the situation that they consider as ideal)

[1] Frequency in grey box corresponds to producers not currently in the situation that they consider as ideal regarding this characteristic

		**Corresponding characteristic for the current situation**		**% of "unhappy" producers**
		>2 cows per hectare of grass	<2 cows per hectare of grass	
**Ideal future farm characteristic**	Intensive	38	66[1]	50%
	Extensive	22	51	
		Only dairy production activity	Presence of activities other than dairy production	
	Specialised	46	59	37%
	Diversified	23	93	
		Presence of milking robot or agricultural equipment for a better technicality	Absence of milking robot or agricultural equipment for a better technicality	
	Strongly based on new technologies	33	52	37%
	Weakly based on new technologies	16	85	
		>1 chief operating officer or associates	1 chief operating officer	
	Managed by a group of managers	20	25	42%
	Managed by an independent farmer	68	108	
		Presence of workers (i.e., external person to family working on the farm)	No workers	
	With family workforce	17	195	10%
	With employed workforce	7	6	
	Providing dairy production for local *vs*. global market	No corresponding characteristic		
	Providing standard *vs*. differentiated quality dairy production	No corresponding characteristic		

Results and discussion

Contrasted opinions of Walloon dairy farmers about the ideal future farm

As mentioned previously, the first aim of this study was to highlight the perceptions of Walloon dairy producers about their ideal farm, just before the end of the milk quota. This was done through the answers to 7 questions. Table 2 shows the frequency for each modality of those questions.

Table 2 Percentages of the seven questions about the ideal future farm

Question	Proposition	Percentage (%)
Intensive *vs*. extensive	Intensive	43
	Extensive	30
	No opinion	27
Specialised *vs*. diversified	Specialised	43
	Diversified	47
	No opinion	10
Strongly *vs*. weakly based on new technologies	Strongly	35
	Weakly	41
	No opinion	24
Managed by an independent farmer *vs*. a group of managers	Independent farmer	72
	Group of managers	18
	No opinion	10
Family *vs*. employed workforce	Family	87
	Employed	5
	No opinion	8
Providing dairy production for local *vs*. global market	Global	43
	Local	32
	No opinion	25
Providing standard *vs*. differentiated quality dairy production	Standard	38
	Differentiated quality	45
	No opinion	17

Contrasted opinions of dairy farmers were observed for almost all questions except for the type of management and the kind of workforce: 71.84% of the respondents wanted an independent farmer management, and 86.53% focused on a family workforce (Table 2). These results highlight a will in the southern part of Belgium to maintain the

traditional form of work organisation in the future, with family workforce and one director of operations. More globally in the world, dairy farms are still mostly owned and managed by a family structure, whatever the degree of development of the country [18, 19]. The choice of producers to work by themselves and not to deal with workers (i.e., an external person to the family employed on the farm) was noted in other studies. For example, in Spain Gonzalez and Gomez (2001) [20] observed, when asking 3,370 farmers for their definition of a farmer, that more than half of them chose labourer and 12% chose businessman.

From Table 2, it is remarkable to note that the highest percentages of abstention were observed for the questions about intensive *vs*. extensive, strongly *vs*. weakly based on new technologies, and providing DP for local *vs*. global markets. These results showed that a quite significant proportion of the respondents did not take a position on these directions for the evolution of dairy farms.

Dualisation of ideal future farm aspirations

To study the relationships between the answers given by the respondents to all questions about IFF, a MCA was performed as the related variables were qualitative (Table 2). The percentage of principal inertia of the dimensions 1 and 2 of MCA were 16.75% and 12.38%, respectively (Fig 1). The value of corrected inertia for the two first dimensions reached 72.7% and 21.5% respectively, gathering almost 95% of the information.

Fig 1. Representation of the modalities in the Multiple Correspondence Analysis first factorial plan. Values of principal inertia reached 16.75% and 12.38%. Values of corrected inertia reached 72.7% and 21.5%.

The first dimension permitted differentiation between the producers who did not give their opinion concerning characteristics of IFF and the producers who did (Fig 1). This represented 15% and 85% of the dataset respectively. The “no opinion” producers group (N = 38) were removed from the analysis to avoid potential bias coming from farmers who did not have a clear vision of their IFF.

The second dimension of the MCA was the most interesting for highlighting the wishes of dairy farmers about their IFF, for those who took position on this question. This axis showed a gradation of question modalities and proximity between several characteristics. This dimension led to the identification of two extreme tendencies (Fig 1); the modalities of familial workforce, independent farmer management and management by a group of farmers were near to zero on this axis (Fig 1). This means that the small proportion of producers supporting group management was distributed between the two extreme tendencies observed. The position of the modalities of familial workforce and independent farmer at the middle of the second dimension illustrated the fact that these modalities were chosen by producers from the two tendencies identified. The small proportion of producers choosing an employed workforce was positioned on the top of the second dimension (Fig 1).

The first tendency, related to high scores on the second MCA dimension, corresponds to IFF with the following characteristics: global market, standard milk, intensive system, employed workforce, specialised and strongly based on new technologies. Other authors have observed the same relations. From a trial of 458 French dairy farms, Hostiou *et al.* (2015) [21] highlighted a profile of farmers which simultaneously gathered high equipment, intensification and workers. From a trial of 3,370 producers of all sectors in Spain, Gonzalez and Gomez Benito (2001) [20] collated the characteristics of large holdings, market-orientated farming and management of workers. Cournut *et*

al. (2010) [22] highlighted different ways of evolving dairy farming in France, characterised by workers, mechanisation and high equipment. This tendency in dairy farming systems is explained by the evolution of the dairy system [4]. The increased competition in the dairy market caused by the creation of the open European market as well as the wish of consumers to have structures that gather all the food supplies in one place (i.e., a supermarket) led to the concentration of dairy processing in some big firms [9]. These firms were better placed to develop because they could control their collection costs, benefit from scale economies and were able to deliver to supermarkets with regularity in quantity and with a standard quality [7]. This state and the world market have conditioned a milk price for the producers. Increasing the production, thanks to more cows or higher productivity, is a possible way to stay profitable, considering the undergone milk price [3, 9]. To achieve profitability, an elevated production of milk per cow and an increase of cows on the farm are reached [9]. Moreover, this increase in milk production at farm level was also forced by the orientated production Common Agricultural Policy (**CAP**) primes, although CAP has limited help to the dairy sector. Therefore, all of these characteristics intensify the dairy farming system. Intensification was defined by Garcia-Martinez *et al.* (2009) [23] as the maximisation of the rarest factor, traditionally the agricultural area. The increase in DP per unit of agricultural area was possible thanks to intensive production of forage and purchase of inputs, produced where production costs were the lowest, to balance the ration, to increase the production per cow, or the number of cows reared on a hectare of agricultural area and therefore DP per unit of agricultural area at the level of the farm [7, 9]. This intensification led to more specialised farms with more dairy cows and their entire workforce directed to this specialisation [7]. The enlargement of farms required a higher work rate; this was surmounted thanks to equipment and new technologies

and thanks to more human workforce: collective organization, subcontracting to private firms and also employment of workers [7].

The second tendency, contradictory to the first tendency, was characterised by high negative scores on the second MCA dimension. This axis was represented by the following modalities: weakly based on new technologies, diversified, differentiated quality milk, local market and extensive system (Fig 1). This reflects another form of dairy farming. This form is favoured by a constant increase in input prices, combined with a growing demand of consumers to have high quality and local based products [7]. Dairy producers choose to work with greater self-sufficiency to be less dependent on the undergone input prices [7]. The “localisation” of the production demanded by consumers was executed thanks to this more local-produced forage and fewer inputs from outside [3]. This return to self-sufficiency led to more extensive farming [3]. The production induced was also often quality-differentiated and dedicated to local markets [7]. Cournut *et al.* (2012) [7] showed in their study that this kind of dairy farming is chosen by a minority of farms, which are still diversified.

This gradation with two kinds of models at the extremities of the second MCA dimension was also described in other studies [3, 4, 7, 9, 24-26]. They were named globalisation *vs*. territorialisation by Cournut *et al.* (2012) [7], or globalisation *vs*. localisation by Napoleone *et al.* (2014) [9]. Lebacq (2015) [4] identified a “dualisation of dairy farming systems between ‘a mainstream model’ focusing on an increasing farm size, production intensity and specialisation and alternative models involving initiatives deviating from this trend and constituting niche developments (niches = minor elements, hardly sustainable against the mainstream model)”.

Thanks to a survey answered by 180 producers of all sectors in 2007 in France, concerning the evolution of their farms and their aspirations, Dockes *et al.* (2007) [25]

also highlighted a major tendency towards the enlargement, professionalisation and specialisation of farms, but those authors also mentioned that other farms wanted to develop diversified structures, orientated towards the requests of society, processing and farm accommodation. The present study showed this dualisation but also quantified these two tendencies: 46% *vs*. 26% of producers having high positive and high negative scores respectively on the second dimension. Verhees *et al.* (2018) [13] quantified producers as a function of their strategies of development, but solely regarding specialisation *vs*. diversification of their activity, 54.3% *vs* 15.1% respectively.

Relationships between ideal future farms and reasons, environmental considerations and formations

To study the relationships between the different IFF and other interesting technico-economic information, the second dimension was considered as a gradient (**IFFg**) interpreted at the extremities as global-based intensive producers (GBI: high positive scores) and local-based extensive producers (LBE: high negative scores). The choice to work with a gradient rather than a clear separation of the two tendencies was motivated by the will to represent all the intermediaries of the IFF of dairy producers. The mean of the scores of the second MCA dimension was –0.012 with a SD of 0.053. Minimal and maximal values were –1.09 and 0.92, respectively.

Based on the interpretation of IFFg, a significant negative correlation indicates a higher relationship with the dairy producers desiring a LBE model. By opposition, a significant positive correlation means a higher link with the dairy producers desiring a GBI model. Tables 3, 5 and 6 give the results of generalised linear models where the qualitative variables were introduced separately as a fixed effect in the model. Significantly lower

estimates of IFFg for a specific modality of the considered qualitative variable depicts a tendency of producers desiring a LBE model to choose this modality, while significantly higher estimates of IFFg means a tendency of producers wanting a GBI model to choose this modality. The following paragraphs will summarise the potential reasons driving the choice of IFF made by the Walloon dairy farmers.

Reasons

Effect of past crisis on perceptions of the ideal future farm The producers that were impacted by past crises wished more for a LBE model (estimate = –0.17, Table 3). This could be related to the suffering involved in the crisis and the wish to apply solutions in order to not repeat this situation: revenue from diversified activities, other outlets for the milk production sold (i.e., local market characteristic) and/or self-sufficiency to be less dependent on purchased feed (i.e., extensive farm characteristic). This is in agreement with a past finding [27]. We observed a decrease in intensification in 2012 which was the year of a dairy economic crisis mainly related to an increase in the price of inputs.

Table 3 Value and level of significance of the difference in the ideal future farm gradient as a function of modalities of qualitative variables: reasons

Qualitative variable	Modality and estimate			*P*
**Past crisis**				
Presence of deep modifications after crisis	No<br>0.031		Yes<br>–0.17	0.025
**Workload**				
Degree of arduousness	Not arduous<br>$–0.11^{b}$	Arduous<br>$–0.092^{b}$	Highly arduous<br>0.15^{a}	0.0043
Member of an agricultural replacement service	Yes<br>0.058		No<br>–0.0801	0.059
Worker engagement: help for workload and administrative aspects	Already implemented<br>0.024^{ab}	To implement in the future<br>0.13^{a}	Not interested<br>$–0.040^{b}$	0.25
**Production factors**				
Milk production evolution for 5 years	Decrease<br>–0.21	Constant<br>–0.13	Increase<br>0.052	0.036

Agricultural area investment since 2009	No –0.14	Yes 0.14				0.0002
Investment (no agricultural area) since 2009	No –0.19	Yes 0.015				0.055
Agricultural area investment in 5 years	No –0.0909	Yes 0.046				0.073
Geographical situation	Brabant Walloon 0.11	Liège 0.12	Namur 0.055	Luxem-bourg –0.013	Hainaut –0.099	0.51
**Age**						
Age (years)	0-34 –0.086	35-44 0.0105	45-54 0.0049	55-64 0.0502		0.67
**Diversification and alternative valorisation**						
Presence of other animal production	Yes –0.093	No 0.0603				0.037
Dairy processing and direct sales: sector developed if supported	Yes –0.33	No 0.11				<0.001
Processing and direct sales (except dairy): sector developed if supported	Yes –0.39	No 0.013				0.0096
Horeca, tourism and teaching activity to develop even if sustained	Yes –0.18	No 0.018				0.055
Concern for diversification	Yes –0.23	No 0.17				<0.001
Alternative chain for milk production valorisation	Yes –0.49	No 0.036				<0.001
Alternative chain for “other than dairy” activity	Yes –0.56	No –0.0087				0.0017
Increase of “other than dairy” activity without investment	Yes –0.42	No –0.013				0.012
No activity to develop even if sustained	Yes 0.27	No –0.10				<0.001
Increase of added value in farms: advantage of diversification and transformation	Yes –0.097	No 0.11				0.0047
Link between producers and consumers: advantage of diversification and transformation	Yes –0.14	No 0.066				0.0064
Conservation of farms in the region: advantage of diversification and transformation	Yes –0.16	No 0.028				0.037
Financial, decisional and technical autonomy: advantage of diversification and transformation	Yes –0.27	No 0.030				0.005
Consumer loyalty: limit to diversification and transformation	Yes 0.17	No –0.047				0.028
Regulatory constraints (hygiene, etc.): limit to diversification and transformation	Yes –0.080	No 0.055				0.065
Size of investments: limit to transformation and diversification	Yes –0.14	No 0.0401				0.030
No constraints for transformation and diversification	Yes –0.093	No 0.042				0.052
No advantage of diversification and transformation	Yes 0.27	No –0.066				0.0006
**Breed**						
Composition of the herd	Single breed 0.18	Multi-breed –0.095				0.0005
	Pure-bred 0.0058	Dual purpose breed: –0.19				0.0023

**Regrouping**					
Advantage of fiscal and administrative aspects: advantage of grouping	Yes<br>–0.16	No<br>0.023			0.050
Development of a joint project: advantage of grouping	Yes<br>–0.15	No<br>0.020			0.072
Better marketing of the products: advantage of grouping	Yes<br>–0.38	No<br>0.0063			0.030
**Mechanisation and robotisation**					
Mechanisation, robotisation: help with workload and administrative aspects	Into effect<br>–0.031^{ab}	Not interested<br>–0.094^{b}	To activate<br>0.12^{a}		0.041
**Reaction to external factors**					
Arduousness of the economic uncertainty of input price	No<br>–0.19	Yes<br>0.0403			0.0089
Will of a dairy factory imposing production limits	Yes<br>–0.23^{c}	No<br>0.25^{a}	Not important<br>0.0088^{b}		<0.001
Evolution of milk production during crises (2009, 2012)	Decrease<br>–0.17^{b}	No variation<br>–0.092^{b}	Increase<br>0.21^{a}		0.0006
Ideal size of the dairy factory	Small<br>–0.52^{c}	Medium<br>–0.11^{b}	Large<br>0.42^{a}	Not important<br>–0.026^{b}	<0.001

Workload Workload seems to be less bearable for producers desiring a GBI model (estimate = 0.15, Table 3; $R_{\text{constraint workforce}}$ = 0.22, P = 0.002). Producers wishing for a GBI model were also nearly significantly more likely to be members of an agricultural replacement service (estimate = 0.058, Table 3) and showed a tendency to be more interested in employment of workers (estimate = 0.13, $P_{\text{worker engagement to implement } vs. \text{ not interested}}$= 0.11, Table 3). The choice of GBI model could be explained by this current workload, involving the need for an increase of revenue. So, the solution considered could be higher milk production and the breeding of more cows rather than diversification of activities and self-valorisation activity, the development of which requires a lot of time. Samson *et al.* (2016) [14] confirmed this in the Netherlands by highlighting a nearly significant effect of labour productivity on the DP increase strategy.

Production factors The size of agricultural area, the milk delivery quota, the number of cows and the percentage of corn silage currently observed in the farming system showed significant and positive correlations with IFFg (R = 0.15, 0.36, 0.18 and 0.24; P = 0.033, <0.001, 0.0099, 0.0002 respectively). So, dairy producers choose their IFF partly as a function of their current production factors. This is expected as a higher

number of hectares, cows and litres means a higher capacity of the dairy installation, of the material and so the possibility of a more preponderant dairy activity. The higher percentage of corn silage also reflected the possibility to seed corn silage, allowing the intensification of production as required within a GBI model. Similar relationships between characteristics of the farm and current or desired models of farming were observed by others. For Central and Eastern Europe, Verhees *et al.* (2018) [13] showed that land was the most important factor in developing a specific farming strategy. In France, Hostiou *et al.* (2015) [21] observed that intensified farms with higher technology equipment sometimes employed more workers, and were the farms with significantly higher agricultural area, percentage of corn silage, number of cows and milk quota. In the Netherlands, Samson *et al.* (2016) [14] showed that production intensity, number of cows, modernity of technology and availability of land were important factors in DP increase strategies.

In contrast, producers with lower production factors can consider more hardly enlargement and therefore think differently about the enhancement of their revenue: better valorisation of quality differentiated milk, other activities on the farm, self valorisation, the LBE model. Samson *et al.* (2016) [14] showed that lower stable capacity varies inversely to a DP increase strategy, which is rather a GBI tendency.

The findings of the current study, as confirmed by previous researchers, showed that producers work within a tightly constrained and regulated environment limiting their ability to determine the future of their farm according to their personal desires. This statement was also concluded by Mc Elwee *et al.* (2006) [28] and Methorst *et al.* (2017b) [29]. In the Netherlands, Keizer and Emvalomatis (2014) [30] and Groeneveld *et al.* (2016) [31] showed that bigger farms are more likely to increase than other farms.

However, based on the quite low values of the correlations obtained between the agricultural area and the number of cows, we can consider that this situation must be nuanced and that the IFF chosen also depends on the ways of thinking of the dairy producer, not taking into account the current situation of his farm. This statement is reinforced by the fact that the correlation of percentage of meadow with IFFg was not significantly different to 0 (R = –0.097, $P > 0.1$). Also, the impact of the provinces of the Walloon Region, which present different geographical and soil characteristics, on IFFg were not significantly different ($P = 0.51$, Table 3).

Moreover the significant relations between IFFg and milk production evolution for five years (Table 3; $R_{\text{quantity of milk variation}} = 0.30$, $P < 0.001$), investment for and in five years (Table 3) support the assumption that the IFF chosen depends greatly on the mentality of the producers.

In their study, Methorst *et al.* (2017a) [11] proved the heterogeneity of farm developments of producers facing the same socio-material context, showing the importance of the mentality of the producers in their decisions. Authors speak about shared values, norms, ways they see themselves or would like to be seen by producers, views, capacities and their perceptions of opportunities and any room for manoeuvre, skills, motives, entrepreneurship, goals and strategies [10, 11, 14, 29, 32] as factors which influence the farm development. Samson *et al.* (2016) [14] discussed experimental economics, which are economics where psychology and biology, which explain human behaviours, are added to better explain the development of enterprises. The consideration of more than just economic aspects permits them to reduce the error of their model for predicting DP increase strategies [14].

Age Age of the producer seems not to condition the desired IFF (Table 3). An IFF could be chosen because of either the new ideas of young producers or the experience

of older producers. If mentality seems to influence IFF choice, it is not linked to age. The two kinds of IFF could be an answer to both innovation and problems encountered during a long career. Samson *et al.* (2016) [14] also studied age as a reflection of the farmers' values, goals and strategies, and showed no relationship with DP increase, which is rather a GBI characteristic. On the contrary, on the basis of data from 11 countries of the European Union, Weltin *et al.* (2017) [12] observed an effect of age on the tendency towards diversification, which is rather a LBE tendency.

Diversification and alternative valorisation The results obtained in this study showed a link between the diversification mentality and the choice of LBE model. Significant negative estimates or correlations were observed for the following variables related to diversification: the presence of other animal production (estimate = –0.093, Table 3); the direct selling milk quota (R = –0.17, P = 0.016); dairy or no dairy processing and direct sales (estimates = –0.33 and –0.39, Table 3); the development of HORECA activities, tourism and teaching (estimate = –0.18, Table 3); the concern for diversification (estimate = –0.23, Table 3); alternative chain for milk and other than milk production valorisation (estimates = –0.49 and –0.56, Table 3) and the increase of "other than dairy" activity without investment (estimate = –0.42, Table 3). Conversely, producers desiring a GBI model were more likely to choose the item "no activity to develop if supported", suggesting the unique principal activity way of thinking of producers aiming for a GBI model (estimate = 0.27, Table 3). Samson *et al.* (2016) [14] confirmed this tendency and showed that the presence of diversified activities evolved inversely to the increase of milk production. In this study, we observed potential explanations to support to this fact. Producers wishing for a LBE model considered self-valorisation and diversification as solutions to the current situation to enhance revenue due to the creation of added value (estimate = –0.097, Table 3).

They thought that diversification and transformation allowed financial, decisional and technical autonomy (estimate = –0.27, Table 3) and were confident in consumer loyalty (estimate = –0.047, Table 3). They considered relations with consumers as an opportunity and not a threat, unlike producers desiring a GBI model (estimate = 0.17, Table 3). One reason GBI model producers gave against self-valorisation and diversification seemed to be the lack of trust in consumers and therefore the outlets. They frequently saw no advantage to self-valorisation and diversification (estimate = 0.27, Table 3). The relation to the consumer was also studied by Verhees *et al.* (2018) [13]. They observed that consumer orientation was more often declared as an opportunity to the profiles of producers considering strategies similar to LBE. The positive impact of diversified activities on autonomy was also shown by Bergevoet *et al.* (2004) [10]. They mentioned that proponents of the “extra source of income” model (closest to the LBE model) were more able to declare that they can increase the sales-price of their milk. Producers wishing for a LBE model were also likely to find no constraints to transformation and diversification (estimate = –0.093, Table 3). The only limits to diversification and transformation highlighted by producers wanting a LBE model were regulatory constraints (estimate = –0.080, Table 3) and the size of investments (estimate = –0.14, Table 3). As a consequence of these considerations, producers wanting a LBE model felt that they were more able to meet society’s expectations regarding local and artisanal products (R = –0.22, P = 0.0016) and the desire for a familial structure (R = –0.12; P = 0.084).

Breed to produce milk Producers wanting a LBE model are more open to breeding a dual-purpose herd (estimate = –0.19, Table 3), which permits them to diversify their production: milk and meat. Producers wishing for a GBI model target a single, more specialised breed (estimate = 0.18, Table 3) which could offer more homogeneous

management of the herd. The link between mentality, observed through the choice of breed(s), and the choice of IFF is once more highlighted.

Regrouping Producers tending towards the LBE model were more likely to promote regrouping for its advantages regarding fiscal and administrative aspects, the development of a joint project and the marketing of the products (estimates = –0.16; –0.15; –0.38, Table 3). The importance of mentality for the choice of IFF has been shown. A mentality of cooperation, as a solution to enhance their quality of life and revenue, tends to be shared between producers desiring a LBE model.

Mechanisation and robotisation A "pro-technology" mentality of the producers tending towards the GBI model was observed (estimate = 0.12, Table 3). It can be assumed that the solution considered by them is to keep the same activity or increase it with help from machines. In southern France, Dufour *et al.* (2007) [33] observed the propensity of farmers with workers, close to the GBI model, to prioritise investment in equipment. Verhees *et al.* (2018) [13] observed that better management, including new technologies, was more cited as an objective for strategy profiles of producers that were more similar to the GBI than LBE models.

Reaction to external factors Reactions of dairy producers to factors external to their decision-making power tend to be different as a function of their choice of IFF, showing once more a different mentality of the producers. Producers wanting a LBE model tend to show themselves to be more independent from the external economic actors: from the input producing companies (estimate = –0.19, Table 3) and from the market and the factories, rejecting contracts which would link them to it (R = –0.13, Table 4). When their opinion about dairy factories was surveyed, producers desiring a LBE model preferred small or medium units with production limits (estimates = –0.52; –0.11; –0.23, Table 3), as before, which means regulation of the dairy offerings on the market.

Producers wishing for a GBI model direct themselves to big units of processing without production limits (estimates = 0.42; 0.25, Table 3) and so more turned towards world markets. They recognise the freedom in regarding DP as an asset of quota removal (R = 0.23, Table 4).

Table 4 Correlations (R) between the ideal future farm gradient and quantitative variables

Quantitative variable	R	*P*
**Reaction to external factors**		
Contract means dairy production more integrated to dairy factories: level of agreement	–0.13	0.076
Quota removal means more flexibility concerning production: level of agreement	0.23	0.0014
**Considerations of the environmental aspects**		
Degree of the constraint: livestock manure application	0.16	0.022
Facility to answer to society's expectations: environmentally friendly agricultural practices	–0.15	0.027
Agricultural activity is important for rurality of villages: level of agreement	–0.23	0.0011
Agricultural activity is important for conservation of permanent grasslands: level of agreement	–0.27	<0.001
Agricultural activity is important for biodiversity: level of agreement	–0.18	0.0101
Agricultural activity is important for planting and maintenance of hedges: level of agreement	–0.28	<0.001
Importance of answering society's expectations for the revenue of the dairy producers: level of agreement	–0.11	0.11
Ease of answering society's expectations: landscape and territory maintenance: level of agreement	–0.19	0.0065
**Needs: formation method**		
*Frequency of calling replacement services for meeting and formations (N = 104)	0.21	0.066

*producers declaring no calling replacement services were removed from this analysis

The reaction regarding the quantity of production was not similar during a crisis, producers wanting a LBE model tended to maintain or decrease their production (estimates = –0.17; –0.092, Table 3), whereas producers desiring a GBI model tended to increase production (estimate = 0.21, Table 3). The latter wanted to keep revenues constant with more litres produced when the price decreased, while the others controlled or decreased production when the gross margin per litre decreased. It can

be due to a deliberate choice to decrease the milk production or the decision to decrease the variables costs causing a decrease ok milk production. These results can express a fear of producers tending toward the LBE model in considering world markets, contrary to producers tending towards the GBI model who have decided to work with this kind of market. Verhees *et al.* (2018) [13] observed that producers projecting strategies similar to the LBE model consider the market more as a threat than producers projecting strategies similar to the GBI model. Hansson *et al.* (2010) [34] and Weltin *et al.* (2017) [12] explained that this uncertainty and risk perception can explain the choice of diversification, which is a part of the strategy of the LBE model.

Couzy and Dockès (2008) [5] demonstrated different profiles of farmers and observed the entrepreneurship mentality of each one, which highlights similar tendencies to those presented here. Several profiles showed strong entrepreneurship but which was expressed differently to here. A category of farmers showed entrepreneurship by their wish for autonomy of decision in their management; they will keep a working approach close to the conventional one but with a modernist vision, always adapting to the market. They want to keep freedom in the classical framework. Another category of farmers showed entrepreneurship by their wish to develop an original idea, away from preexisting systems, a project in line with their conviction to be freer from the existing system.

Samson *et al.* (2016) [14] and Methorst *et al.* (2017a) [11] reported that decisions of producers cannot be reduced to only economic aspects: this includes policies and market conditions but also their way of thinking about them.

Consideration of environmental aspects

The environmental aspects related to the desired IFF is now studied as awareness of the environmental impact of breeding has become an important issue of our time.

Producers tending toward the GBI model seemed to work with a higher livestock manure application pressure (R = 0.16, Table 4) and therefore are already more to work in an intensified dairy system, which can impact the environment. Samson *et al.* (2016) [14] showed a tendency toward manure production surplus by producers with increasing DP, which is rather a GBI characteristic.

Results of practices that are in accordance with the environment: measurement of the grass height, forage mixture with leguminous plants, use of a field notebook (estimates = –0.27; –0.11; –0.074, Table 5) showed a stronger interest from producers wanting a LBE model.

Table 5 Value and level of significance for the difference in the ideal future farm gradient as a function of modalities of qualitative variables: environmental aspects

Qualitative variable	Modality and estimate		*P*
**Considerations of the environmental aspects**			
Measurement of the grass height: optimisation practice	Yes<br>–0.27	No<br>0.0083	0.059
Forage mixture with leguminous plants: optimisation practice	Yes<br>–0.11	No<br>0.083	0.0088
Field notebook: optimisation practice	Yes<br>–0.074	No<br>0.061	0.065
**Climatic hazard**			
Increase of concentrate distribution: strategy to confront climatic hazards	Yes<br>0.22	No<br>–0.036	0.036
Decrease of the herd: strategy to confront climatic hazards	Yes<br>–0.25	No<br>0.014	0.037
Food self-sufficiency: cause for maintaining constant or decreased milk production	Yes<br>–0.17	No<br>0.0073	0.14

Besides these, all the significant negative correlations between IFFg and the levels of agreement with an agricultural area are important for the rurality of villages (R = –0.23,

Table 4), for conservation of permanent grasslands (R = –0.27, Table 4), for biodiversity (R = –0.18, Table 4) and for hedges (R = –0.28, Table 4) showed the importance of the environment in the dairy activity of producers wanting a LBE model. It can be assumed that both LBE producers and GBI producers have concern for the environment but in a different way. These results showed that LBE producers are more willing to employ the benefits of ecosystem services, which is observable by these results. Moreover, they found it easy to realise environmentally friendly agricultural practices, as asked for by society (R = –0.15, Table 4) and which are important to answer to society's expectations to guarantee their revenue (R = –0.11, Table 4).

Bergevoet *et al.* (2004) [10] had a considerably more consistent opinion. The "extra-source of income" profile producers (showing similarities with the LBE model) were more likely to declare that in their decision-making they take the environment into consideration, even if it lowers profit. The "large and modern farm" profile producers do not mention their will to adopt these initiatives.

Climatic hazard Facing feed shortages due to unfavourable climatic conditions, producers tending toward GBI and LBE seem not to have the same way of thinking; GBI producers intend to buy high nutritional feed to balance the shortages (estimate = 0.22, Table 5) and LBE producers are going to decrease the number of cows (estimate = –0.25, Table 5) and ensure their feed autonomy (estimate = –0.17, Table 5).

Current situation vs. ideal future farm

The current situation of dairy producers was compared to their preferred IFF (Table 1). Except for the type of workforce, quite high percentages of "unhappy" producers were observed for the farm characteristics, between 37 to 50%. This suggested that not all

producers work as they would like to. The same comparison was not found in the literature, to our knowledge.

As dairy producers do not work in a way that they consider to be ideal, it is interesting to study the gaps to fill in order to reach their ideal system and so, amongst others, their needs. The study of the requirements to reach the IFF, including ways to meet these needs and the area of the needs, can inform the stakeholders of the dairy sector about what must be developed to evolve into IFF.

Needs

Paths to formation As way to improve their skills, producers wanting GBI tended to favour consultancy (estimate = 0.17, Table 6) and commercial companies (estimate = 0.16, Table 6) and not days of study on other farms (estimate = 0.082, Table 6), meanwhile producers wanting LBE supported this latter possibility (estimate = –0.088, Table 6), a network of pilot farms (estimate = –0.13, Table 6) and the associate, not market sector (estimate = –0.21, Table 6). Moreover, for help in technical choices, producers desiring LBE chose formation and study days (estimate = –0.15, Table 6) and producers' technical groups to implement in the future (estimate = –0.20, Table 6). The choices presented confirm the will for a non-market way to learn for producers wanting LBE, contrary to producers wishing for GBI.

As an information source, the agricultural press was commonly cited (N = 161, i.e., 78% of respondents), but producers desiring LBE tend to not want to inform themselves in this conventional way (estimate =–0.14, Table 6).

Producers wanting a GBI model tend to need more help to free them from their work in order to follow a formation (R = 0.21, Table 4)

Table 6 Value and level of significance of the difference in the ideal future farm gradient as a function of modalities of qualitative variables: formations

Qualitative variable	Modality and estimate			P
**Needs : ways' formation**				
*Consultancy company: SFI place	Yes<br>0.17		No:<br>–0.087	0.0017
*Study days on farm: SFI place	Yes<br>–0.088		No<br>0.082	0.026
*Network of pilot farms: SFI place	Yes<br>–0.13		No<br>0.056	0.025
*Associate, non-market sector SFI place development	Yes<br>–0.21		No<br>0.062	0.0023
*Commercial company: SFI place	Yes<br>0.16		No<br>–0.058	0.014
**Agricultural press: information source	Yes<br>0.025		No<br>–0.14	0.068
Formation and study day: help for technical choices	Already implemented<br>0.037[a]	To implement in the future<br>–0.15[b]	Not interested<br>0.035[a]	0.082
Producers technical groups: help for technical choices	Already implemented<br>0.020[a]	To implement in the future<br>–0.20[b]	Not interested<br>0.11[a]	0.0046
**Needs : domains' formation**				
*Finance and management: requested formation	Yes<br>0.066		No<br>–0.24	0.0007
*Processing and diversification: requested formation	Yes<br>–0.18		No<br>0.089	0.0008
*Plant selection: requested formation	Yes<br>0.083		No<br>–0.053	0.087
*Animal selection: requested formation	Yes<br>0.080		No<br>–0.082	0.034
*Animal feeding: requested formation	Yes<br>0.03		No<br>–0.14	0.073
*Administrative: requested formation	Yes<br>0.064		No<br>–0.11	0.026
*Legal framework: requested formation	Yes<br>0.14		No<br>–0.083	0.005
Request for advice: help for financial aspects	Already implemented<br>0.014[a]	To implement in the future<br>–0.15[b]	Not interested<br>0.049[a]	0.19

SFI = study, formation and information

*producers declaring no will of formation were removed from this analysis

** producers declaring no agricultural press as an information source were removed from this analysis

Formation domains The formation domains reflected the direction chosen by producers looking for LBE and the ways to reach it. They tend to want skills related to processing and diversification (estimate = –0.18, Table 6) and were likely to reject finance, management (estimate = –0.24, Table 6), administrative (estimate = –0.11, Table 6) and legal framework (estimate = –0.083, Table 6) skills. For financial aspects

producers wanting LBE tend to favour requests for advice from experts rather than self-formation (estimate = –0.15, $P_{\text{to implement } vs. \text{ not interested}}$ = 0.12, Table 6). They do not choose animal feeding (estimate = –0.14, Table 6) and selection formations (estimates = –0.053; –0.082, Table 6). This could suggest the will of the producers not to change their way of management and the level of quality of their herd but the method of valorisation of their production.

In contrast, producers desiring GBI tend to want to continue enhancement of their vegetal and animal production (estimates = 0.083; 0.08, Table 6), to become more efficient and enhance their revenue. Moreover they are more interested in legal aspects (estimate = 0.14, Table 6). Expansion and complexification of the GBI model of dairy farms wished for by these producers could be an explanation. Bergevoet *et al.* (2004) [10] also observed a will to be well informed about the legislation for the "modern and large farm" profile. This is not noted in their profile, which is close to the LBE model.

Two kinds of formation were identified and preferred by producers wanting LBE or GBI models. Bergevoet *et al.* (2014) [10] observed the will to innovate for the two profiles closest to LBE and GBI profiles of this study. Verhees *et al.* (2018) [13] observed that formation was the most important resource for dairy producers. The present research differentiated the formation desired as a function of IFF. Dufour *et al.* (2007) [33] defined, through a survey of 15 dairy farmers, three conceptions of the work: difficult, organisational and passionate. The passionate approach was accompanied by the desire for new knowledge which was, as observed here, either to learn about genetic selection or about processing and marketing of products.

Conclusions

In conclusion, the GBI tendency is two times more represented than the LBE tendency. Many reasons explain this choice of ideal farm. Past crises seem to cause farmers to desire the LBE model. A high workload seems to orientate respondents to the GBI model. The wish for the IFF is influenced by the current framework but is also a question of mentality. Production factors reached, breeds chosen for the herd, ways to react to factors external to the farm, consideration of diversification and alternative valorisation, regrouping, and mechanisation and robotisation describe the producers' mentality and showed different relations with the IFF chosen. Moreover LBE and GBI producers may both have concern for environment, but the approach to act for the environment by LBE producers, through concern for ecosystem services, is clearly highlighted in this study. These producers found it important to answer to society's expectations. Finally, as the current situation of farming is quite different to the ideal one, the needs for learning were studied and two types of customer appeared within dairy producers in relation to their formation. We conclude that two kinds of producers seem to appear, for different reasons, with different relations to the environment and asking for different formations.

Acknowledgments

I want to thank the organising committee of Carrefour des Productions animales for the supply of the data.

References

[1] European Economic Community. Treaty of Rome; 1957.

[2] STATBEL. Exploitations agricoles et horticoles-plus de chiffres ; 2019. [Cited 2019 Feb 14]. Available from: https://statbel.fgov.be/fr/themes/agriculture-peche/exploitations-agricoles-et-horticoles/plus

[3] Havet A, Cournut S, Correa P, Madelrieux S, Poccard R, Corniaux C, *et al.* Tandem changes in livestock farming and feed systems since 1960: a study of six dairy-producing regions. Fourrages. 2015;222:115–24. 12.

[4] Lebacq T. La durabilité des exploitations laitières en Wallonie - Analyse de la diversité et voies de transition. M.Sc. Thesis, UCLouvain. 2015. Available from: http://www.philagri.net/wp-content/uploads/2015/03/TL_redaction_these_20150302.pdf

[5] Couzy C, Dockes A-C. Are farmers businesspeople? Highlighting transformations in the profession of farmers in France. Int J Entrep Small Bus . 2008;6(3):407–20.

[6] Direction générale opérationnelle de l'agriculture. Evolution de l'économie agricole et horticole de la Wallonie 2013-2014 ; 2014. Service Public de Wallonie, Namur, Belgium.

[7] Cournut S, Rapey H, Nozières MO, Poccard-Chapuis, R., Corniaux C, Choisis JP, *et al.* Dynamics of livestock farming in extensive livestock farming territories : what processes are going on ? In: 10th European IFSA Symposium. Aarhus, Denmark; p. Unpaginated Ref. 26.

[8] Food and Agriculture Organization of the United Nations (FAO). Livestock's Long Shadow. Environmental issues and options. 2006; 1-380.

[9] Napoléone M, Boutonnet JP, Corniaux C, Alavoine-Mornas F, Barritaux V, Carvalho S, *et al.* Between local and global : which reconfiguration within Dairy Basins ? A comparative analysis from North & South case-studies. In: 10th European Social Science History Conference. Vienna, Austria; p. 1–12.

[10] Bergevoet RHM, Ondersteijn CJM, Saatkamp HW, Van Woerkum CMJ, Huirne RBM. Entrepreneurial behaviour of dutch dairy farmers under a milk quota system: Goals, objectives and attitudes. Agric Syst. 2004;80(1):1–21.

[11] Methorst RG, Verhees FJHM, Verstegen JAAM. Differences in farmers' perception of opportunities for farm development. NJAS - Wageningen J Life Sci. 2017;81:9–18. doi : 10.1016/j.njas.2017.02.001

[12] Weltin M, Zasada I, Franke C, Piorr A, Raggi M, Viaggi D. Analysing behavioural differences of farm households: An example of income diversification strategies based on European farm survey data. Land use policy. 2017;62:172–84. doi : 10.1016/j.landusepol.2016.11.041

[13] Verhees F, Malak-Rawlikowska A, Stalgiene A, Kuipers A, Klopčič M. Dairy farmers' business strategies in Central and Eastern Europe based on evidence from Lithuania, Poland and Slovenia. Ital J Anim Sci. 2018;17(3):755–66. doi : 10.1080/1828051X.2017.1422154

[14] Samson GS, Gardebroek C, Jongeneel RA. Explaining production expansion decisions of Dutch dairy farmers. NJAS - Wageningen J Life Sci . 2016;76:87–98. doi : 10.1016/j.njas.2015.11.007

[15] Service public de Wallonie. La Carte des Sols de Wallonie; 2016. [Cited 2019 July 15]. Available from http://geoportail.wallonie.be/catalogue/ce3b6602-1c52-483f-9133-770009cdd02b.html

[16] Palm R. L'analyse à correspondances multiples : principes et application. Gembloux : Notes de statistique et informatique ; 2007.

[17] Benzécri J P. Sur le calcul des taux d'inertie dans l'analyse d'un questionnaire. Cahiers de l'analyse des données. 1979;4, pp. 377-378.

[18] De Haan C. Module 4- Smallholder dairy production. In Agriculture Investment Sourcebook, 2011 ; pp.169-173. World Bank, Washington, United States of America.

[19] United States Department of Agriculture/Economic Research Service (USDA/ERS) The U.S. Dairy Industry, A Vital Contributor To Economic Development. 2014.

[20] González JJ, Gómez Benito C. Profession and identity. The case of family farming in Spain. Sociol Ruralis. 2001;41(3):343–57.

[21] Hostiou N, Chauvat S, Cournut S. Faire face à des questions de travail : les leviers mobilisés par des éleveurs laitiers. In: INRA, editor. L'agriculture en famille : travailler, réinventer, transmettre. Paris France; 2015. p. 125–43.

[22] Cournut S, Servière G, Hostiou N, Chauvat S, Dedieu B. L'organisation du travail en exploitations familiales d'élevage. Enseignements d'une analyse comparée conduite en France, en Amérique latine et au Vietnam. Cah Agric. 2010;19:338–47.

[23] García-Martínez A, Olaizola A, Bernués A. Trajectories of evolution and drivers of change in European mountain cattle farming systems. Animal. 2009; 3, 152–165.

[24] Mc Elwee G and Robson A.Diversifying the farm: Opportunities and barriers. Finnish Journal of Rural Research and Policy. 2005; 4, 84–96.

[25] Dockes A-C, Couzy C, Daridan D, Gallot S, Magdelaine P, Pilorgé E, *et al.* Transformation de l'agriculture et des métiers des agriculteurs: quelles conséquences pour les organismes de Développement ? In: Journées INRA-SFER de recherches en sciences sociales. p. 18.

[26] Lémery B. Les agriculteurs dans la fabrique d'une nouvelle agriculture. Sociol Trav. 2003;45(1):9–25.

[27] Dalcq A-C, Beckers Y, Wyzen B, Reding E, Delhez P and Soyeurt H. Behavior patterns to the intensification vary differently within dairy producers. Proceedings of the 69th EAAP meeting, 27-30 August 2018, Dubrovnik, Croatia, 2018:252.

[28] Mc Elwee G. The enterprising farmer: a review of entrepreneurship in agriculture. J R Agric Soc Engl. 2006;January:1–9.

[29] Methorst R, Roep D, Verstegen J, Wiskerke JSC. Three-fold embedding: Farm development in relation to its socio-material context. Sustain. 2017;9(10):1–19.

[30] Keizer TH, Emvalomatis G. Differences in TFP growth among groups of dairy farms in the Netherlands. NJAS - Wageningen J Life Sci. 2014;70:33–8. doi : 10.1016/j.njas.2014.03.001

[31] Groeneveld A, Peerlings J, Bakker M, Heijman W. The effect of milk quota abolishment on farm intensity: Shifts and stability. NJAS - Wageningen J Life Sci. 2016;77:25–37. doi : 10.1016/j.njas.2016.03.003

[32] Willock J, Deary IJ, Edwards-Jones G, Gibson GJ, McGregor MJ, Sutherland A, *et al.* The Role of Attitudes and Objectives in Farmer Decision Making: Business and Environmentally-Oriented Behaviour in Scotland. J Agric Econ. 1999;50(2):286–303. doi : 10.1111/j.1477-9552.1999.tb00814.x

[33] Dufour A, Hostiou N, Cournut S, Dedieu B. Work organization on the dairy farm : visions of labour , working units and type of management varied. In: Rencontre Recherche Ruminants. Paris; p. 385–8.

[34] Hansson H, Richard F, Olofsson C. Understanding the diversification and specialization of farm businesses. Agric Food Sci . 2010;19:269–83.

Figure

Click here to access/download;Figure;Ideal future dairy farms to ensure revenue figure.eps

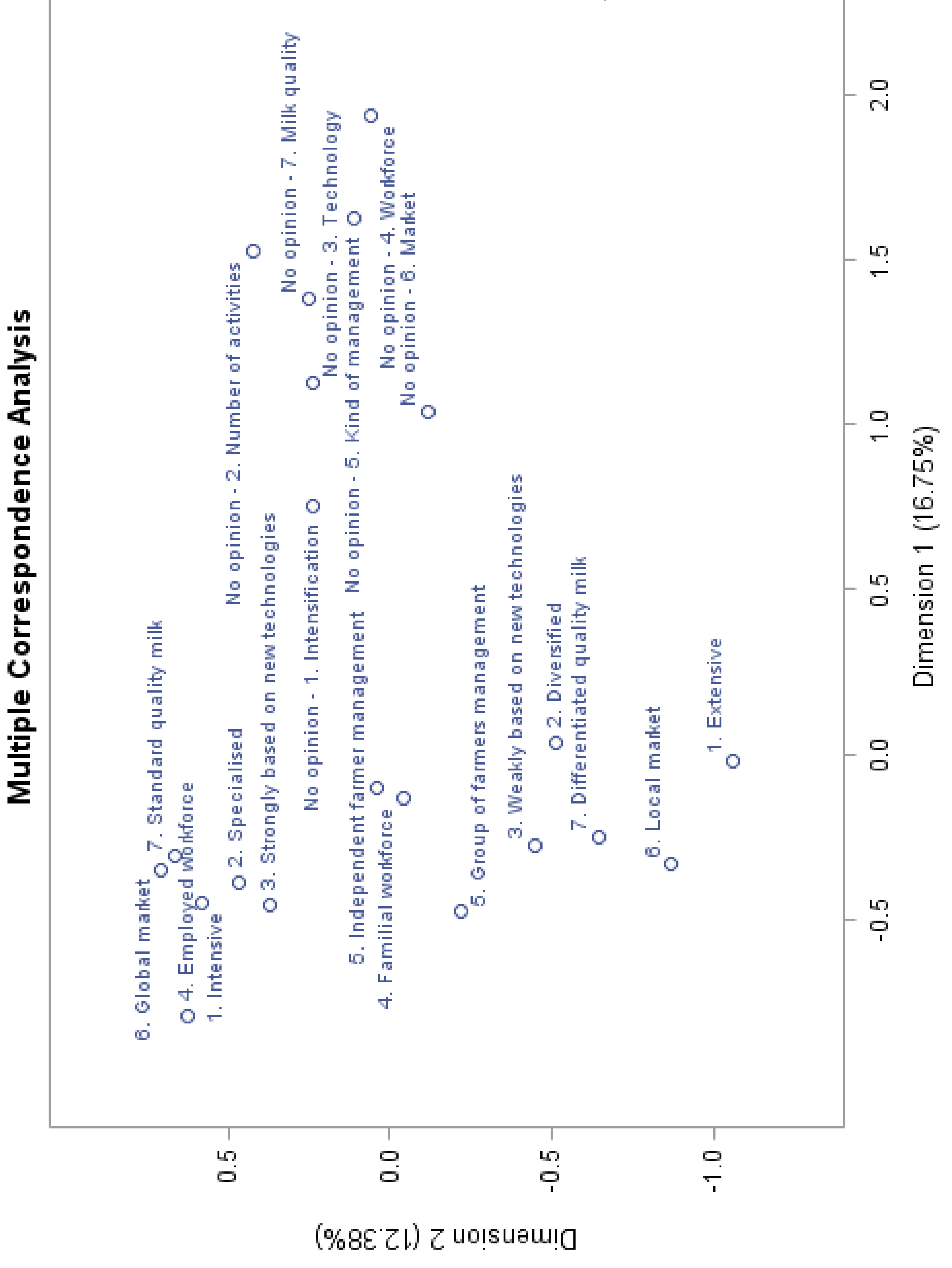

Other

Click here to access/download
Supporting Information
letter Ethics comittee.PNG
