# Supplementary material for: The Walloon farmers position differently their ideal dairy production system between a global-based intensive and a local-based extensive model of farm
Source: PLoS One. 2020 Dec 3;15(12):e0223346. doi: 10.1371/journal.pone.0223346 (PMC7714150; doi:10.1371/journal.pone.0223346)
Supplement: Attachment — Submitted filename: PONE-D-19-26278_R2.pdf [file pone.0223346.s005.pdf]

PLOS ONE

How do dairy farmers wish their future farm?

--Manuscript Draft--

**Manuscript Number:**	PONE-D-19-26278R2
**Article Type:**	Research Article
**Full Title:**	How do dairy farmers wish their future farm?
**Short Title:**	Ideal future dairy farms to ensure revenue
**Corresponding Author:**	Anne-Catherine Dalcq<br>Universite de Liege Gembloux Agro-Bio Tech<br>Gembloux, BELGIUM
**Keywords:**	Dairy farming systems; future; producers; reasons; environment; climatic hazard; formation
**Abstract:**	Dairy farming systems are evolving. This study presents dairy producers' perceptions of their ideal future farm (IFF) to ensure revenue, and attempts to determine the reasons for this choice, the environmental aspects related to this choice, the proximity between the current farm and the IFF and the requirements for reaching this IFF. Just before the end of the European milk quota, a total of 245 Walloon dairy producers answered a survey about the characteristics of their IFF and other socio-environmental-economic information. A multiple correspondence analysis (MCA) was carried out using seven characteristics of the IFF (intensive vs. extensive, specialised vs. diversified, strongly vs. weakly based on new technologies, managed by a group of managers vs. an independent farmer, employed vs. familial workforce, local vs. global market, standard vs. quality-differentiated production) to observe the relationships between them. Based on the main contributors to the second dimension of the MCA, this axis was defined as an IFF gradient between the local-based extensive (LBE) producers (26%) and the global-based intensive (GBI) producers (46%). The differences of IFF gradient between modalities of categorical variables were estimated using generalised linear models. Pearson correlations were calculated between the scores on the IFF gradient and quantitative variables. Finally, frequencies of IFF characteristics and the corresponding characteristic for the current situation were calculated to determine the percentages of "unhappy" producers. Some reasons for the choice of IFF by the producers have been highlighted in this study. Environmental initiatives were more valued by LBE than GBI producers. Low similarity was observed between the current farm situation of the respondents and their IFF choice. LBE and GBI producers differed significantly regarding domains of formation (technical and bureaucratic vs. transformation and diversification respectively) and paths of formation (non-market vs. market respectively). Two kinds of farming systems were considered by dairy producers and some socioeconomic and environmental components differed between them.
**Order of Authors:**	Anne-Catherine Dalcq
	Thomas Dogot
	Yves Beckers
	Yves Brostaux
	Eric Froidmont
	Frédéric Vanwindekens
	Hélène Soyeurt
**Response to Reviewers:**	All these elements are in the document : Response to reviewers.<br>Sincerely,<br>Anne-Catherine Dalcq<br>Dear Academic Editor, dear Reviewers,<br>First, I would like to thank you for the time spent to improve the understanding of this manuscript. This letter is organized in two parts. The first part deals with the main issue of the review (i.e., the method mobilized, which corresponds to the point raised by the

Powered by Editorial Manager® and ProduXion Manager® from Aries Systems Corporation

academic editor and one remark of the reviewer). The second part concerns the specific answers to the specific remarks formulated by the reviewers.
Sincerely,
Anne-Catherine Dalcq
1/ Methodology
Please read carefully the propositions of reviewer #2 especially about the recommendations you did not took into account in your revision. I am afraid the reviewer is right about the interest of LCA vs MCA, as we expect a characterization of different profiles of dairy farmers.
But my proposition is that instead of LCA, you use HCPC (Hierarchical Clustering on Principal Components) which a natural and standard extension of MCA (see Arguelles et al. 2014, Kassambara 2017 or the following technical report http://factominer.free.fr/more/HCPC_husson_josse.pdf).
H. Soyeurt :
Dear Editor,
Dear Reviewers,
Instead of my PhD student, Miss Anne-Catherine Dalcq, I would like to answer to the question related to the methodology used in this study. I am Professor Hélène Soyeurt and I teach courses related to Data Mining and Machine Learning at Gembloux Agro-Bio Tech (University of Liège). Therefore, I have an experience in the use of multivariate analysis. It is why I would like to answer by myself to the question related to the method used in this article.
Before starting an explanation, I would like to precise that we have not really understood the comments of the reviewer about Latent Class Analysis (LCA) because the objective was not to create groups of farmers. Indeed, and this is the innovative aspect of this paper, a gradient between two quite different models of farms was studied. The gradient is really important because it appears to us simplistic to classify farmers in only 2 groups, working with a gradient allowed us to nuance the position of the dairy producers and to analyze more precisely the link between this position and other characteristics. The use of the gradient allows studying the trend of a farmer. It is why in this paper, we always mention “tend towards” to make a reference to the position on the gradient and not a binary choice of a model.
As we did not want to create farmer’s clusters, LCA was not appropriate as well as HCPC. However, as asked by the second reviewer to prove the robustness of our approach, we have decided to show you the similarities and the extended work that it is possible to do using Multiple Correspondence Analysis (MCA) and LCA. Indeed, it is also possible to use LCA to create a gradient instead of using the clusters.
So, I would like to remind the methodology that we have proposed in this study (see the figure below, Fig 1)). Again, MCA was used to observe the relationships between the seven studied variables. Based on the interpretation of MCA dimensions, we have observed that the second dimension represented the positioning between the two models for a dairy farm. Therefore, the score for this dimension for a specific producer allows to know its perception of ideal future farm between the two extreme models. This gradient allows avoiding to limit the farm typology to 2 clusters. This is interesting because many farmers combined some approaches specific to one model of farms (no more intensification but extensification, local-based market) or another one (global-based market, continuously improvement of the productivity thanks to, notably, intensification, …). Therefore, wishing to split the dairy farmers into 2 groups is too limitative. Some farmers remain between these two models (Fig 2), some farmers are more convinced than others by a model. Farmers showing higher scores are really convinced by GBI model, farmers showing lower but positive scores choose GBI model but are not 100% in this way of farm development. Negatives scores express the position of farms in favor of LBE model, the lowest scores reflected pronounced adhesion to this model. It is why the gradient as proposed in this paper was useful and seem to us the most interesting tool to represent dairy producers.

Figure 1 Methodology

Figure 2 Distribution of the producers (the "with an opinion" ones) along the second dimension of the MCA
Similarly, we have done this job using LCA methodology as requested by the reviewer#2. LCA allows defining clusters from the dataset. In this case and to be in line with the study objective, we have decided to create 3 clusters. Moreover, based on the AIC and BIC values, it was also the best model (Fig 3). LCA allows us to create those 3

Powered by Editorial Manager® and ProduXion Manager® from Aries Systems Corporation

clusters. After the interpretation of defined clusters, it appeared that we have a cluster representing “No opinion” responders, one cluster representing “global-based intensive” (GBI) farmers and one cluster representing “local-based extensive” (LBE) farmers (Fig 4). Therefore, those clusters were similar to the ones obtained by combining MCA and Ward clustering (=HCPC method) as done to clean the dataset (i.e., extract responders with “no opinion” behavior). Again, we did not want to use those clusters to make our analysis. But, using this methodology, it is possible to obtain a probability to belong to a specific cluster. Therefore, we have decided to compare the gradient as defined in the current study to the probability to belong to the LBE or GBI clusters defined using the LCA method.
So, now, it is time to present you the results. I will not present you the results about MCA as those results are reported in the article. In this paragraph, we will focus on the LCA results. As we used categorical variables which are not ordinal, we have decided to use the polytomous latent class analysis. The variables used, called manifest variables, were the same than the one used for MCA. The modalities for each variable were recoded from 1 to 3:
Intensification: 1. Intensive, 2. Extensive, 3. No opinion
Number of activities: 1. Specialised, 2. Diversified, 3. No opinion
Technology: 1. Strongly based on new technologies, 2. Weakly based on new technologies, 3. No opinion
Workforce: 1. Familial workforce, 2. Employed workforce, 3. No opinion
Kind of management: 1. Group of farmers management, 2. Independent farmer management, 3. No opinion
Market: 1. Global market, 2. Local market, 3. No opinion
Milk quality: 1. Standard quality milk, 2. Differentiated quality milk, 3. No opinion

As this clustering is very sensitive to the prior values used to start the iteration, 10 repetitions were used to provide you the final results for all models. All calculations were done with R software and more specifically the package poLCA. First, we have run different LCA models using a different number of classes (clusters). We have tested models from 1 to 10 classes and then we have estimated the AIC and BIC criteria to observe which model allowed the best fitting. The results of AIC and BIC for all models are presented in the figure below (Fig 3). From this figure, we can conclude that the model allowing the creation of 3 classes is a good comprise between BIC and AIC (i.e., the lowest BIC and AIC values).

Figure 3 AIC & BIC criteria values
The figure below shows you the clusters defining by the model allowing a discrimination of the data into 3 clusters (Fig 4). This figure represents the probability to have a specific modality for each cluster. So, based on those results, we can conclude that the first clustering (class 1) is related to GBI dairy producers, the second cluster (class 2) is related to LBE dairy producers and the last cluster (class 3) is related to the dairy producers with “no opinion”.

Figure 4Description of the three clusters obtained by LCA method
Therefore, in order to clean the dataset (i.e., delete records from farmers having many “no opinion” views), we can use the clustering 3. This process is similar to the MCA + Ward clustering (HCPC) proposed in the manuscript. Again, we did not want to use clusters but we want to use a gradient. For MCA, this gradient was the second MCA dimension. In the context of LCA, this gradient can be derived from the probability to belong to class 1 (“GBI producer”) or class 2 (“LBE producer”). So, to show the robustness of the MCA approach used, we calculated the correlation between those probabilities and the score of the second MCA dimension (called gradient in the manuscript). The correlation between the probability to belong to class 1 and the gradient was equal to 0.83. The correlation between the probability to belong to class 2 and the intensification gradient was equal to -0.87. The correlation between the 2 probabilities was equal to -0.97.
From all of those results, you can see that the relationship is strong between MCA and LCA using an innovative approach focusing on the score/probability of an individual and not directly to a cluster. Using only the second dimension of MCA, we can reflect both clusters (class 1 and class 2) simultaneously. Indeed, the second dimension is a gradient “GBI-LBE” and included both. This is really interesting to observe the relationships between this gradient and other quantitative or qualitative variables as now the studied trait is quantitative. It allows to see if a modality of a categorical

Powered by Editorial Manager® and ProduXion Manager® from Aries Systems Corporation

variable is the choice of really convinced GBI dairy producers or dairy producers only sticking out of the GBI model.
I hope that this demonstration illustrates well the relevancy of the approach proposed in our paper. A sentence will be added in the materials and methods section to explain why LCA was not used. Moreover, some articles exist also in the literature to prove the mathematical relationships between MCA and LCA (e.g., Lautsch and Plichta, Psychology Science 2003:298-323 as well as Van der Heijden, et al., Sociological methodology 1999).
The fact that the two dimensions explained almost 95% of the variability and that all the modalities representing an opinion positioning themselves along the second dimension support us to take the second dimension as gradient, after deletion of the No-opinion producers which were discriminated by the first dimension.
Finally, I would like to acknowledge you for the great job done in the review of this paper. The manuscript was improved a lot.
Sincerely,
Prof Hélène Soyeurt
PS : The answers to all other comments were done by Anne-Catherine Dalcq.
2/ Others comments
Reviewer #2: In this revision, the authors have significantly improved the English language translation and clarified their research questions, but they have failed to address the shared concerns of reviewers regarding methods and clarity of writing. They have not adequately addressed the substance of the comments from reviewers. Regarding methods, the reviewers still do not offer a compelling and clear justification for why MCA is appropriate for their goals and they do not offer the latent class analysis that I suggested or the hierarchical clustering (related) suggested by reviewer 3 as alternatives or robustness checks. MCA, while performed adequately, is not well suited to the way they discuss their results. They continually refer to "types" of respondents, which is what latent class analysis is for. MCA is about identifying clustering of variables, not clusters of respondents. All of their interpretation of results is about clustering and patterns of PEOPLE, not variables. This indicates a significant misalignment between the method and the research goals.
A-C Dalcq: First, we would like to thank you for the deep reading done on this article and for your formulated comments. We missed your request to test the robustness of our method with other ones. We apologize for this mistake. You will find our work of comparison with LCA method in the first part of this letter. In the article, we speak about producers tending towards "GBI" or "LBE" models to express the results coming from the use of our gradient. Indeed, MCA is a method to identify relationships between variables but is also suitable to make groups of individuals thanks to the use of its extension HCPC, as proposed by the academic editor. A detailed information about the comparison of MCA and LCA is now given in the first part of this letter.

The authors do not adequately discuss the two dimensions of the MCA (figure 1) and the axes are not adequately labeled. It is not made clear why the second dimension is retained.
A-C Dalcq: We added some information. The updated explanations of the two dimensions are taken up just after (Lines 254-261/286-306/339-343). Do you need more information? If it is the case, could you guide us?
Remark : Lines specified throughout this letter are those of the revised manuscript with track changes.
The axes are labeled as the figure is provided by SAS 9.4. We precised its meaning thanks to the caption and the following interpretation.
Explanations of the two dimensions:
Lines 254-261: "The first dimension of MCA showed positive relationships with the modalities no opinion of each characteristic and negative relationships with all the modalities representing an opinion. Thus, the first dimension of the MCA allowed permitted differentiation between the producers who did not give their opinion concerning characteristics of IFF and the producers who did (Fig 1). Cluster analysis was used to isolate the group of producers with a lot of 'no opinion' answers to the seven questions: this formed the first separation of classes of the analysis, dividing the "no-opinion" producers (15%) from the others (85%)."
Lines 286-306: "The second dimension of the MCA showed positive relationships with some modalities of the IFF characteristic and negative relationships with their opposite. Thus, this dimension seems was the most interesting for highlighting to highlight the wishes of dairy farmers about their IFF, for those who took a position on this question.

Powered by Editorial Manager® and ProduXion Manager® from Aries Systems Corporation

More precisely, this axis showed a gradation of question modalities and proximity between several characteristics. This dimension led to the identification of two extreme tendencies (Fig 1); the modalities of familial workforce, independent farmer management and management by a group of farmers were near to zero on this axis (Fig 1). This means that the small proportion of producers supporting group management was distributed between the two extreme tendencies observed. The position of the modalities of familial workforce and independent farmer at the middle of the second dimension illustrated the fact that these modalities were chosen by producers from the two tendencies identified. The small proportion of producers choosing an employed workforce was positioned at the top of the second dimension (Fig 1)."
"The first tendency, related to high scores on the second MCA dimension, corresponds to IFF with the following characteristics: global market, standard milk, intensive system, employed workforce, specialised and strongly based on new technologies."
Lines 339-343: "The second tendency, contrary to the first tendency, was characterised by negative scores on the second MCA dimension. This axis was represented by the following modalities: weakly based on new technologies, diversified, differentiated quality milk, local market and extensive system (Fig 1). This reflects another form of dairy farming. »
Reason of the use of the second dimension:
Lines 379-384: "To study the relationships between the different IFF, the reasons for these and other interesting technico-economic information, the second dimension was considered as a gradient (IFFg) interpreted at the extremities as global-based intensive producers (GBI: high positive scores) and local-based extensive producers (LBE: high negative scores). The choice to work with a gradient rather than a clear separation of the two tendencies was motivated by the will to not put dairy producers into boxes pigeonholes"

While the authors have provided a link to the survey online, that link is only in French and requires registration with an email address before it can be viewed, so that is not adequate. The authors still have not addressed the real concern that I raised in my previous review: they need to be clear about how they measured their concepts (intensive/extensive, etc.), what specific survey questions were used, and how those variables were coded. A clear list of questions for each concept and the coding for each is needed. For instance, in Table 2, it is not clear what specific survey questions or variables represent these concepts and how those variables were actually coded.
A-C Dalcq: In the current version, we have added an annex (Appendix 1) with the translation of the questions mobilized in the paper. Given the length of the survey, we provided only the questions raised in the present paper. Some information are mentioned at lines 129-136 about the questions related to the ideal future farm characteristics (intensive vs. extensive,…), the way of measurement for our developed concepts. If you need more information, could you precise them to us explicitly ?
Lines 129-136: "The entire survey was composed of 127 questions where the answers were decomposed into 498 categorical and 44 quantitative variables. The question 'Without taking into account your current farm, what is, according to you, the ideal future farm to ensure a revenue?" was proposed to the producers and they must could choose between short propositions on seven items: 1) intensive or extensive production; 2) specialised or. diversified activity (or activities); 3) farming strongly or weakly based on new technologies; 4) farm managed by an independent farmer or a group of managers; 5) family or employed workforce; 6) providing production for local or global markets; 7)providing standard or differentiated quality production. The modality "no opinion" was available for each IFF question."

The authors still do not address response rate for the survey. They do now address representativeness of their respondents for this specific region, but they have not addressed the bigger questions of representativeness: how does this one region in one nation represent that nation, Europe, and/or agriculture broadly?
A-C Dalcq: The response rate of 6,1% was already precised in the past manuscript. You can find it at lines 200-201 of the current text.
Lines 200-201 : "The sample set of 245 producers represented 6.1% of the dairy producers in Wallonia (about 4,000 dairy producers in 2015 and 3,500 in 2017 (STATBEL, 2019))."
For the second part of your comment, do you want that we precise that the Walloon Region is one of the two regions of Belgium, which is one of the 27 members countries

Powered by Editorial Manager® and ProduXion Manager® from Aries Systems Corporation

of the European Union? If yes, the proposal could be : “The Walloon Region is one of the two regions of Belgium, which is one of the 27 members countries of the European Union”.
Or do you want that we precise the number of dairy producers in Belgium and in the European Union? The International Dairy Federation mentions 9,674 dairy farms in Belgium and 1,130,700 farms with a dairy activity in the European Union (Confédération Belge de l’Industrie Laitière, 2020). The number of Walloon dairy farms is obviously low amongst all of these countries. We do not know if all this information is relevant to be written in the manuscript.
Complete reference: Confédération Belge de l’Industrie Laitière. 2020. Rapport Annuel 2020.
The goal of this paper is to inform about the position of dairy producers of a region, which is moreover quite heterogeneous regarding the geopedologic conditions (lines 205-206), this one can represent the context and the resources of other producers in Europe. The goal was not to give a complete vision of all the European producers, which needs higher means.

In all tables the n, or respondent totals, should be clear.
A-C Dalcq: We added a sentence at lines 395-396 :“These analysis were conducted on the producers who have an opinion (N = 207).” And the N was precised and added in each table. Thanks for this remark which brings clarity throughout all the paper.

For Table 5 there are subscripts/footnotes that are never defined or labeled.
A-C Dalcq: The subscripts are now precised in each table. “Means with different letters are significantly different.”. Thanks for this remark.

For Tables 3 and 4, no significance tests are reported.
A-C Dalcq: Indeed, the goal was not to test the differences between the no-opinion producers and all the sample but to give an idea of the characteristics of the No-opinion producers. Reviewer#3 asked us to give all of this information also for producers with an opinion. We realized ANOVA tests between the no-opinion producers and the producers with an opinion (lines 280-284)

The interpretation of the MCA results is circular logic. They define the clusters based on variables such as the attitude toward technology and then present a finding that people who are in the “pro-technology” GBI cluster have more positive attitudes towards technology. Of course, that is how you defined the scale in the first place.
A-C Dalcq: The variable “technology” is “Mechanisation and robotisation : help for workload and administrative aspects “ and is present in the part “Reasons”. This result is presented to explain one reason of the producers tending towards “GBI-model” to tend to this model and one of its component, the technology. We have better precised our idea by adding the sentence: “We observed that the wish of technology of producers tending towards GBI model can be explained by the fact that they considered it as help for workload.” at lines 534-536.

Regarding writing, the presentations of results and its mixing with discussion of existing literature is still extremely unclear and difficult to follow. Both reviewer 1 and myself raised this critique: presenting your results intermingled with other literature is difficult to read and makes it unclear what your key findings are. This is not about the technical requirements of the journal. In its current presentation, readers cannot easily identify what your key findings are in each subsection and it is very difficult to read. For instance, in the section on pages 15-16, the authors spend substantially more time discussing other studies than they do their own results.
A-C Dalcq: As already mentioned and visible at lines 304-364 in the revised paper, the explanations based on the findings of other past studies help to explain the relationships observed between the modalities of the seven ideal future farm characteristics.

The introduction is improved, but still weak. The first paragraph is overly general and does nothing to build the focus of the paper. The authors also spend too much time asserting the contribution of their study before they have even reviewed the literature or told us what their analysis will be.
A-C Dalcq: What could be your expectations about the structure of this introduction? As the reviewer#3 did not make comments about the redaction of this part and without

Powered by Editorial Manager® and ProduXion Manager® from Aries Systems Corporation

deeper expectations from your, the structure of the introduction was not changed.

The attempts to incorporate new literature are cursory.
A-C Dalcq: The work of Mr Mooney was consulted and two references was added to the paper (lines 236-238, lines 577-580). Did you expect references to more elements of his work? Could you precise which ones? Moreover, we also investigated the phenomenon of bifurcation. Literature about bifurcation was mainly found for the organic activity. We consulted a Professor with skills in sociology, Prof Kevin Maréchal, of Gembloux Agro-Bio Tech-University of Liège (Belgium), who provided us also this literature reference. Do you have other literature to advice to me?

The paper is not appropriately written for a general audience. They assume too much prior knowledge from readers regarding methods both methods and the case.
A-C Dalcq: We have now added information about the choice of the method between MCA and LCA, and about the method WARD (regarding a following remark). We hope that it brings the missing information. If this information is not complete for you, could you precise us explicitly the requested information?
Lines 165-170: "This method was chosen instead of the creation of classes, possible with the Latent Class Analysis method or the Numerical Classification on the scores of MCA (Hierarchical Clustering on Principal Components). This choice was motivated by the wish to not put the producers in boxes but study their position on a gradient between potential extreme models identified along the dimension."
Lines 153-156 : "The WARD method is a hierarchical agglomerative method (Everitt et al., 2011). The principle of this kind of method is to put initially the n individuals in n groups and then to agglomerate the groups. The algorithm of WARD makes it in such a way that the gatherings induce the lowest decrease of R2 at each step."

Overall, the authors have inadequately addressed the careful feedback of the reviewers and made inadequate improvements. Throughout the response to reviewers they reject several important critiques with no justification of their rejection.
A-C Dalcq: We recognized that we do not explain the choice of the method and we do not test its robustness with statistic treatments. We apologized for that. We missed this request. We realized this analysis at this time.

A number of specific points are highlighted below:
Line 79- How was that ensured (respondent producers were asked not to take into account their current farm when considering their IFF)?
A-C Dalcq: It is now precised in the Materials and methods section (Lines 131-134). Thanks for your remark.
Lines 131-134 : The question 'Without taking into account your current farm, what is, according to you, the ideal future farm to ensure a revenue?" was proposed to the producers and they must choose between short propositions on seven items: 1) intensive or extensive production; 2)[…]"

Line 121- survey link not accessible without registering. Include in appendix?
A-C Dalcq: The appendix is realized and available in the new submission.

Line 146- What is WARD?
A-C Dalcq: We added sentences of explanation about the Ward method at lines 153-156. It is a hierarchical agglomerative method of Numerical classification.
Lines 153-156: "The WARD method is a hierarchical agglomerative method (19). The principle of this kind of method is to put initially the n individuals in n groups and then to agglomerate the groups. The algorithm of WARD makes it in such a way that the gatherings induce the lowest decrease of R2 at each step."

Line 145- What are "particular characteristics" beyond no-opinion profiles?
A-C Dalcq: We replaced particular by "some" (Lines 151). We hope it makes it clearer.

Line 157- What?
A-C Dalcq: This sentence is a part of the method. As explained and precised before, we realized a MCA on the seven ideal future farm questions. We observed that the

Powered by Editorial Manager® and ProduXion Manager® from Aries Systems Corporation

modalities “no-opinion” of the seven questions gathered and were positively related with the first dimension of the MCA. All the modalities reflecting an opinion were negatively related to the first dimension. Thus, the first dimension allowed to differentiate the producers with an opinion or not.
The modalities “intensive”, “global-market”, “specialized”, “standard quality milk”, “employed workforce” and “strongly based on new technologies” gathered and were positively related to the second dimension. The modalities “extensive”, “local-market”, “diversified”, “quality differentiated milk” and “lowly based on new technologies” gathered and were negatively related to the second dimension. The second dimension appeared to us as a gradient between the two extreme models of ideal future farm “Global-based intensive” and “Local-based extensive”.
The two dimensions explained almost 95% of the variability of the dataset. Therefore, the study of only these two dimensions appeared to us relevant. Then, we realized a numerical classification on the scores on the two dimensions of the MCA (statistic treatment equivalent to HCPC- Hierarchical Clustering on Principal Components). The first two groups created were the “no-opinion” producers and the producers “with an opinion”. This allowed us to exclude the “no-opinion” producers and to study the producers with an opinion thanks to the second dimension, this one had at its extremities the “Local-based extensive model” and the “Global-based intensive model”. But the producers with an opinion distributed themselves along this dimension. Thus, we decide to work with the second dimension, as a gradient of ideal future farm (Fig 5).

Then we want to study the relationships between this ideal future farm gradient and the other information present in the survey.
The relationships between the ideal future farm gradient and the categorical variables of the survey were studied thanks to generalized linear models.
The gradient was the y, the variable to explain. The modalities of the categorical variables were the fixed effect of the generalized linear model, the factors explaining.
y = effect + residual
Where y was a vector contained the score on the ideal future farm gradient (the second dimension of MCA); effect was the qualitative variables of the survey. In other words, the model was :
Ideal future farm gradient = categorical variable + e
To study the relationships between the gradient and the quantitative variables of the survey, correlation coefficients and their level of significance were calculated.

Line 160- What are the quantitative variables?
A-C Dalcq: Quantitative is a statistical term defining a continuous numerical variable. Do you want that we use the term « numerical » ? But this term is less precise as it does not reflect the continuous dimension of the variable.

Table 1- What are the ns? Maybe a total figure? Percentages?
A-C Dalcq: Absolute frequencies named counts. We precised at lines 186 and 194: “Absolute frequencies (counts)”.

How many questions in each dimension?
A-C Dalcq: One question. We have now mentioned that in the Materials and methods section at Lines 187-188: “and of the answer to the question which corresponds to this corresponding characteristic for the current situation”.

Line 180- specifics of response rate still missing.
A-C Dalcq: The response rate is 6,1% (Line 200). Which supplementary indication do you need? We gave you numbers of farms with a dairy activity in Belgium and in European Union in this letter. We give also more information about the conditions where the survey was communicated to the producers to give you an idea of the way of proceeding. More information is given at lines 120-126.
Lines 120-126: “We communicated with Walloon dairy producers about the goals of the survey and its access broadly via all communication ways towards them : specialised press, agricultural internet websites, Unions and also advertisements through the milk payment letter which is sent to all the Walloon dairy producers once a month. The survey written in French can be viewed at the following internet link:

Powered by Editorial Manager® and ProduXion Manager® from Aries Systems Corporation

https://www.gembloux.ulg.ac.be/enquete/index.php/219425?lang=fr and its English translation is viewable in the Appendix“

Table 2- list questions?
We have added the question in the table (Line 223).
Question
Without taking into account your current farm, what is, according to you, the ideal future farm to ensure a revenue?” PropositionPercentage (%)
Intensive vs. extensiveIntensive43
Extensive30
No opinion27
Specialised vs. diversifiedSpecialised43
Diversified47
No opinion10
Strongly vs. weakly based on new technologiesStrongly35
Weakly41
No opinion24
Managed by an independent farmer vs. a group of managersIndependent farmer72
Group of managers18
No opinion10
Family vs. employed workforceFamily87
Employed5
No opinion8
Providing dairy production for local vs. global marketGlobal43
Local32
No opinion25
Providing standard vs. differentiated quality dairy productionStandard38
Differentiated quality45
No opinion17

Totals
A-C Dalcq: It is now mentioned at line 223.

Figure 1 define dimensions. What are the percentages in the axes labels?
A-C Dalcq: The percentages in the axes labels are the inertia but these ones underestimated the part of information explained by the dimensions. The corrected inertia values were calculated. This is explained in the Materials and methods section at lines 142-146: “For a MCA, the eigenvalue of the dimensions generated, named principal inertia, is a biased measure of the amount of information presented by a dimension (Palm, 2007). Corrected inertia rates were calculated, as described by Benzécri (Benzécri, 1979), to quantify the correct proportion of information of a dimension.”
The corrected values of inertia are presented at lines 248-250. The nature of the values are precised in the caption of the figure (lines 251-253).
“The percentage of principal inertia of the dimensions 1 and 2 of MCA were 16.75% and 12.38%, respectively (Fig 1). The value of corrected inertia for the two first dimensions reached 72.7% and 21.5% respectively, gathering almost 95% of the information.
Fig 1. Representation of the modalities in the multiple correspondence analysis first factorial plan. Values of principal inertia reached 16.75% and 12.38%. Values of corrected inertia reached 72.7% and 21.5%.”
The correction of Benzécri is made following this calculation:
corrected inertia= $\left(\frac{s}{s-1}\right)^2 * (\mu_k - 1/s)^2$ with $\mu_k > 1/s$
s = number of categorical variables in the MCA
μ_k= eigenvalue of the dimension
(0.33508 and 0.2475, eigenvalues of respectively the first and second dimension of the present MCA)
The corrected inertia gives a better appreciation of the amount of information explained by each dimension (Benzécri, 1979), than the inertia automatically provided by the software.
We did not think necessary to precise this in the paper but we provide the reference where this calculation is presented.

Powered by Editorial Manager® and ProduXion Manager® from Aries Systems Corporation

Line 335- where is this figure reference from
A-C Dalcq : We did not provide a representation of the distribution of the producers along the second dimension, we did not think it was necessary as we provided the percentages and this figure would lengthen the paper. But following your request, we have now added this figure in the present manuscript (Lines 376-377).

Fig 2. Distribution of the producers along the second dimension (the dotted line represents the mean score on the second dimension of the producers)(N = 207)

Table 5-footnotes? Which is LBI and which GBI
A-C Dalcq : We were not sure about the goal of your question. We precised the function of the letters a and b. Concerning your request about the LBE and GBI, as explained with more details in the point 1/, the analysis is not done between groups LBE or GBI and the variables present in the survey but between an ideal future farm gradient and the variables present in the survey. The means presented in the tables 5, 7 and 8 are the mean value of this gradient for the different modalities of the categorical variables. We explained this at lines 390-395.
Lines 390-395: "Tables 5, 7 and 8 give the results of generalised linear models where the categorical variables were introduced separately as a fixed effect in the model. Significantly lower estimates of IFFg for a specific modality of the considered categorical variable depicts a tendency of producers desiring a LBE model to choose this modality, while significantly higher estimates of IFFg means a tendency of producers wanting a GBI model to choose this modality."
What test is used here?
A-C Dalcq: Generalised linear models were used to study the level of significance of the differences between the means values of the gradient of the modalities of categorical variables: y was the ideal future farm gradient and the effect included in the model was the categorical variable. We explained this part of the method in the Materials and methods section at lines 178-184.
Lines 178-184: "For categorical variables, the scores of MCA dimensions were modelled using these variables as a fixed effect in a generalised linear model. Least squares means were estimated for the two-by-two comparisons using the Tukey test. The level of significance of those differences was assessed based on the P-value of the test. For quantitative variables, Pearson correlation coefficients were calculated between the scores of MCA dimensions and these variables. Their corresponding P-values were estimated to observe if the correlation values were significantly different from 0."

Line 356 explain what they mean by introduced as fixed effect
A-C Dalcq: As explained in the point 1/ of this letter, the gradient was used as y (variable to be explained) of the generalized linear models and the fixed effect introduced in the generalized linear model was the categorical variables. See the previous answer related to the same topic for more details.

Line 620- What is SFI
A-C Dalcq : We precised this at line 661. "SFI = study, formation and information ». It is after the table. We have now added an asterisk to highlight the explanation of this abbreviation.

Reviewer #3: Thanks to the authors, ho made significant improvements to the paper. The full potential of the data is now revealed in the analysis. I particularly appreciate the improvements on the description of the « no-opinion » farmers, as suggested in my first review.
A-C Dalcq: Thank you for the interest given to this study.

Specific comments:
84: add references to « This change implied the disappearance of regulation of dairy supplies and caused volatility and decrease in the milk price »
A-C Dalcq: We added a reference and precised our purpose (Lines 83-84). Thanks for your remark.
Lines 83-84: "This change implied the disappearance of regulation of dairy supplies and was bringing uncertainty about the milk price (16). caused volatility and decrease in the milk price. "

Powered by Editorial Manager® and ProduXion Manager® from Aries Systems Corporation

Reference 16: Salou, T., H.M.G. van der Werf, F. Levert, A. Forslund, J. Hercule, and C. Le Mouël. 2017. Could EU dairy quota removal favour some dairy production systems over others? The case of French dairy production systems. Agric. Syst. 153:1–10. doi:10.1016/j.agsy.2017.01.004.

99-100: the question #2 is unclear. « What is the proportion of producers desiring the different IFF? » should be rephrased a bit maybe. The expression « the different IFF » will be vague for the readers. Is the IFF always different from the current farm ? And I guess « their IFF » is more thuitable thant « the IFF » as each respondent will provide a personal definition of their IFF.
A-C Dalcq: We understand your will to make this question clearer. We have replaced it by « How the dairy producers distribute themselves between IFF highlighted ?" (Lines 100-101, lines 365-366).

122: you do not answer to another reviewer's comment, who wanted to know the response rate to the interview. To how many farmers was this survey submitted ? e.g. number of farmers buying the « specialised press », number of advertisements sent with the milk payment letter etc.
A-C Dalcq: We precised in the text that all the Walloon dairy producers received this payment letter: +/- 4,000 producers. Therefore, the 245 respondents correspond to 6,1% of the population, i.e. the response rate. We added information at lines 120-126 & 200-201. We do not know if the information number of farmers buying the « specialised press », number of advertisements sent with the milk payment letter are necessary in this paper but we added information to give an idea of the conditions in which the survey was communicated.
Lines 120-126: "We communicated with Walloon dairy producers about the goals of the survey and its access broadly via all communication ways towards them : specialised press, agricultural internet websites, Unions and also advertisements through the milk payment letter which is sent to all the Walloon dairy producers once a month. The survey written in French can be viewed at the following internet link: https://www.gembloux.ulg.ac.be/enquete/index.php/219425?lang=fr and its English translation is viewable in the Appendix.
A total of 245 producers completed our survey between November 2014 and January 2015."
Lines 200-201: "The sample set of 245 producers represented 6.1% of the dairy producers in Wallonia (about 4,000 dairy producers in 2015 and 3,500 in 2017 (STATBEL, 2019)."

240:253 : the description of the no-opinion farmers adds value to the data analysis. However I am not sure that the last sentence is useful. This is your personal interpretation, but the data do not permit to reveal it.
A-C Dalcq: Indeed, this sentence was deleted (Line 279). Thanks for your remark.

Tables 3 and 4: you have two columns, which are « complete sample » and « no-opinion farmers ». A third column which reflects the sample excluding the no-opinion farmers will permit the reader to compare the no-opinion ones with the others.
A-C Dalcq: Indeed, this column was added (Lines 280, 283). And, as asked by reviewer#2, we realized (1) generalized linear models to compare the means of the quantitative variables between the no-opinion producers and producers with an opinion and (2) tests of proportion to compare the proportion of each modalities of the categorical variables between the no-opinion producers and producers with an opinion. We added description of these statistical treatments in the Materials and Methods section (Lines 157-160).

333 : as already said, I think this title is not well written and could be more explicit.
A-C Dalcq: It was changed at lines 365-366. See also the answer to your previous comment related to the same topic.

350 : pigeonholes. Could you be more precise?
A-C Dalcq: As it caused doubt in your comprehension of this idea, we replaced by boxes (Line 384).
Put people (here dairy producers) in boxes.
It was used to explain the fact to put a label of someone. But in this paper, we want to nuance the position of the producer regarding its ideal future farm.

Powered by Editorial Manager® and ProduXion Manager® from Aries Systems Corporation

Additional Information:	
**Question**	**Response**
**Financial Disclosure**<br><br>Enter a financial disclosure statement that describes the sources of funding for the work included in this submission. Review the submission guidelines for detailed requirements. View published research articles from *PLOS ONE* for specific examples.<br><br>This statement is required for submission and **will appear in the published article** if the submission is accepted. Please make sure it is accurate.<br><br>**Unfunded studies**<br>Enter: *The author(s) received no specific funding for this work.*<br><br>**Funded studies**<br>Enter a statement with the following details:<br>• Initials of the authors who received each award<br>• Grant numbers awarded to each author<br>• The full name of each funder<br>• URL of each funder website<br>• Did the sponsors or funders play any role in the study design, data collection and analysis, decision to publish, or preparation of the manuscript?<br>• **NO** - Include this sentence at the end of your statement: *The funders had no role in study design, data collection and analysis, decision to publish, or preparation of the manuscript.*<br>• **YES** - Specify the role(s) played.<br><br>* typeset	The author(s) received no specific funding for this work.
**Competing Interests**<br><br>Use the instructions below to enter a competing interest statement for this submission. On behalf of all authors, disclose any competing interests that could be perceived to bias this work—acknowledging all financial support and any other relevant financial or non-financial competing interests.	The authors have declared that no competing interests exist.

Powered by Editorial Manager® and ProduXion Manager® from Aries Systems Corporation

This statement **will appear in the published article** if the submission is accepted. Please make sure it is accurate. View published research articles from *PLOS ONE* for specific examples.<br><br>**NO authors have competing interests**<br><br>Enter: *The authors have declared that no competing interests exist.*<br><br>**Authors with competing interests**<br><br>Enter competing interest details beginning with this statement:<br><br>*I have read the journal's policy and the authors of this manuscript have the following competing interests: [insert competing interests here]*<br><br>* typeset	
**Ethics Statement**<br><br>Enter an ethics statement for this submission. This statement is required if the study involved:<br><br>• Human participants<br>• Human specimens or tissue<br>• Vertebrate animals or cephalopods<br>• Vertebrate embryos or tissues<br>• Field research<br><br>Write "N/A" if the submission does not require an ethics statement.<br><br>General guidance is provided below. Consult the submission guidelines for detailed instructions. **Make sure that all information entered here is included in the Methods section of the manuscript.**	The Board of Ethics and Scientific Integrity of the University of Liege waives the need for ethical approval. We communicated with Walloon dairy producers about the goals of the survey and its access via specialised press, and advertisements through the milk payment letter which is sent to all the Walloon dairy producers once a month. The survey can be viewed at the following internet link: https://www.gembloux.ulg.ac.be/enquete/index.php/219425?lang=fr"<br><br>I agree with this ethics statement.

**Format for specific study types**<br><br>**Human Subject Research (involving human participants and/or tissue)**<br>• Give the name of the institutional review board or ethics committee that approved the study<br>• Include the approval number and/or a statement indicating approval of this research<br>• Indicate the form of consent obtained (written/oral) or the reason that consent was not obtained (e.g. the data were analyzed anonymously)<br><br>**Animal Research (involving vertebrate animals, embryos or tissues)**<br>• Provide the name of the Institutional Animal Care and Use Committee (IACUC) or other relevant ethics board that reviewed the study protocol, and indicate whether they approved this research or granted a formal waiver of ethical approval<br>• Include an approval number if one was obtained<br>• If the study involved *non-human primates*, add *additional details* about animal welfare and steps taken to ameliorate suffering<br>• If anesthesia, euthanasia, or any kind of animal sacrifice is part of the study, include briefly which substances and/or methods were applied<br><br>**Field Research**<br><br>Include the following details if this study involves the collection of plant, animal, or other materials from a natural setting:<br>• Field permit number<br>• Name of the institution or relevant body that granted permission	
**Data Availability**<br><br>Authors are required to make all data underlying the findings described fully available, without restriction, and from the time of publication. PLOS allows rare exceptions to address legal and ethical concerns. See the PLOS Data Policy and FAQ for detailed information.	No - some restrictions will apply

Powered by Editorial Manager® and ProduXion Manager® from Aries Systems Corporation

A Data Availability Statement describing where the data can be found is required at submission. Your answers to this question constitute the Data Availability Statement and **will be published in the article**, if accepted.<br><br>**Important:** Stating 'data available on request from the author' is not sufficient. If your data are only available upon request, select 'No' for the first question and explain your exceptional situation in the text box.<br><br>Do the authors confirm that all data underlying the findings described in their manuscript are fully available without restriction?	
**Describe where the data may be found in full sentences. If you are copying our sample text, replace any instances of XXX with the appropriate details.**<br><br>• If the data are **held or will be held in a public repository**, include URLs, accession numbers or DOIs. If this information will only be available after acceptance, indicate this by ticking the box below. For example: *All XXX files are available from the XXX database (accession number(s) XXX, XXX.).*<br>• If the data are all contained **within the manuscript and/or Supporting Information files**, enter the following: *All relevant data are within the manuscript and its Supporting Information files.*<br>• If neither of these applies but you are able to provide **details of access elsewhere**, with or without limitations, please do so. For example:<br><br>*Data cannot be shared publicly because of [XXX]. Data are available from the XXX Institutional Data Access / Ethics Committee (contact via XXX) for researchers who meet the criteria for access to confidential data.*<br><br>*The data underlying the results presented in the study are available from (include the name of the third party*	Data were collected by the Committee of 'Carrefour des Productions Animales', a committee of stakeholders of animal sector in the Walloon Region of Belgium (research center, agricultural unions, university). The data are stored on the CAMI platform of Gembloux Agro-Bio Tech. A request can be made to the President of the platform Mr Gengler (Nicolas.Gengler@uliege.be) to get data.

Powered by Editorial Manager® and ProduXion Manager® from Aries Systems Corporation

and contact information or URL).<br>• This text is appropriate if the data are owned by a third party and authors do not have permission to share the data.<br><br>* typeset	
Additional data availability information:	

Powered by Editorial Manager® and ProduXion Manager® from Aries Systems Corporation

Manuscript Click here to access/download;Manuscript;Manuscript.docx

How do dairy farmers wish their future farm?

Anne-Catherine Dalcq[1]*, Thomas Dogot[1], Yves Beckers[1], Yves Brostaux[1], Eric Froidmont[2], Frédéric Vanwindekens[2], Hélène Soyeurt[1]

[1] University of Liège, Gembloux Agro-Bio Tech, TERRA Teaching and Research Centre, Passage des Déportés 2, 5030 Gembloux, Belgium

[2] Walloon Research Centre, Rue du Liroux, 8, 5030 Gembloux, Belgium

*Corresponding author

E-mail : anne-catherine.dalcq@uliege.be (ACD)

Ideal future dairy farms to ensure revenue

Abstract

Dairy farming systems are evolving. This study presents dairy producers' perceptions of their ideal future farm (**IFF**) to ensure revenue, and attempts to determine the reasons for this choice, the environmental aspects related to this choice, the proximity between the current farm and the IFF and the requirements for reaching this IFF. Just before the end of the European milk quota, a total of 245 Walloon dairy producers answered a survey about the characteristics of their IFF and other socio-environmental-economic information. A multiple correspondence analysis (**MCA**) was carried out using seven characteristics of the IFF (intensive *vs.* extensive, specialised *vs.* diversified, strongly *vs.* weakly based on new technologies, managed by a group of managers *vs.* an independent farmer, employed *vs.* familial workforce, local *vs.* global market, standard *vs.* quality-differentiated production) to observe the

relationships between them. Based on the main contributors to the second dimension of the MCA, this axis was defined as an IFF gradient between the local-based extensive (**LBE**) producers (26%) and the global-based intensive (**GBI**) producers (46%). The differences of IFF gradient between modalities of categorical variables were estimated using generalised linear models. Pearson correlations were calculated between the scores on the IFF gradient and quantitative variables. Finally, frequencies of IFF characteristics and the corresponding characteristic for the current situation were calculated to determine the percentages of “unhappy” producers. Some reasons for the choice of IFF by the producers have been highlighted in this study. Environmental initiatives were more valued by LBE than GBI producers. Low similarity was observed between the current farm situation of the respondents and their IFF choice. LBE and GBI producers differed significantly regarding domains of formation (technical and bureaucratic *vs.* transformation and diversification respectively) and paths of formation (non-market *vs.* market respectively). Two kinds of farming systems were considered by dairy producers and some socioeconomic and environmental components differed between them.

Introduction

Food is a basic need. Working to provide food for themselves and their family was the task of everyone at the dawn of humanity. The progressive organisation of society during the Neolithic period has led to the appearance of “producers” who are responsible for producing food for more than just themselves and their family (1,2). Since World War II, public policies have been set up to increase food production (3). These policies impacted the development of producers and their farms in the European Union. In the southern part of Belgium, the mean number of cows and the mean agricultural area per producer increased between 1980 and 2017 from 20 to 66 heads and from 25 to 71 hectares, respectively (4).

Producers are now facing great challenges to stay profitable. The price of the inputs (e.g. buildings, agricultural machinery, installations, feeding, veterinary care) of dairy production (**DP**) are increasing while the milk price shows great variability and its inflation is not similar to that observed for the inputs (5,6). Moreover, the European Union has decreased financial support to farmers (7). On 1st April 2015, the European Union removed the quota system which had managed the supply of DP (8). This led to greater milk price volatility. Additionally, sanitary crises such as mad cow disease (bovine spongiform encephalopathy (BSE)) and the dioxine crisis, among others, have shocked consumers and led to new rules and regulations at European level and to the creation of food security agencies in its countries. Moreover, these episodes modified consumers’ behaviours regarding their food purchases, they asked for more transparency and directed themselves towards organic food or local chains (9). Besides the economic view, the impacts of farming on the environment have been noted and policies have been set up in the Common Agricultural Policy to solve these problems (6,10).

In this context, the question often asked by dairy producers and stakeholders of the dairy sector is what the future of dairy farming entails, how to remain profitable and more generally sustainable. Several authors, such as Napoléone *et al.* (11), Havet *et al.* (5) and Lebacq (6), have studied the evolution of dairy farming and the present dairy systems, finding trends that exist in the sector. For instance, the project Mouve, funded by the French National Research Agency, studied the evolution of dairy farming systems in 6 dairy basins around the world. Their results gathered the publications of Napoléone *et al.* (11) and Havet *et al.* (5). Moreover, some other authors (e.g., Bergevoet *et al.* (12), Methorst *et al.* (13), Weltin *et al.* (14) and Verhees *et al.* (15) have studied the future paths of development considered by dairy producers. These studies were performed on the basis of data from 2001 to the beginning of 2013. They explored some reasons for these choices for the future.

This study is innovative as it asks what is the ideal future farm (**IFF**) perceived by the dairy farmers to ensure revenue. To our knowledge this question is not present in other studies. Moreover, respondent producers were asked not to take into account their current farm when considering their IFF. The data collection was conducted more recently, at the end of 2014 and the beginning of 2015. This was a particular context, just before the quota removal, when producers had this new perspective in mind. This change implied the disappearance of regulation of dairy supplies and was bringing uncertainty about the milk price (16). We have assumed that this change in their working framework impacted respondents reflections and led them to reconsider their strategies, taking into account this new reality. They had just faced two important milk crises associated with low milk price in 2009 and an increase of the cost of inputs in 2012. This research studied unprecedented reasons for the choice of IFF compared to what is present in the literature, to our knowledge, such as past events of the farms.

Moreover, the present study explored the environmental and training aspects linked to this IFF vision. The environmental aspect is of high importance at a time of increasing awareness of the impacts of agriculture and breeding on the environment such as carbon footprints, biodiversity, etc. The topic of trainings for dairy producers was studied to orientate universities and other stakeholders of breeding improvement towards the domains needed and desired by dairy producers. A comparison between the current farm and the IFF of the respondent was realised, and permitted the difference between the reality and the aspiration of the producers to be studied. More specifically, the goals of this study were to answer the following questions: (1) What is the perception of dairy producers of their IFF? (2) How do dairy producers distribute themselves between IFF highlighted? By gathering different kinds of information, of which some are novel or rarely present in the literature, this study also answered to the following questions: (3) How do farmers decide on their IFF? (4) How do environmental aspects factor into IFF decisions? (5) Which paths and themes for training do farmers want in order to reach their desired IFF? And, ultimately, (6) how do farmers' IFF compare to their current dairy farming systems?

Materials and methods

Survey and IFF typology

In 2014, moving towards the end of the quota, as stakeholders of the dairy sector (research centre, agricultural sciences faculty, breeding association, agricultural unions, etc.), we wanted to know how the dairy producers of the southern part of Belgium will react to this change. We created a survey using LimeSurvey software (version 3.15.1+181017, LimeSurvey GmbH, Hamburg, Germany), which provides an internet link to get access and to complete the survey. The survey was first pre-tested orally with two dairy producers to estimate its duration and its clarity. The Board of Ethics and Scientific Integrity of the University of Liege waives the need for ethical approval. We communicated with Walloon dairy producers about the goals of the survey and its access broadly via all communication ways towards them : specialised press, agricultural internet websites, Unions and also advertisements through the milk payment letter which is sent to all the Walloon dairy producers once a month. The survey written in French can be viewed at the following internet link: https://www.gembloux.ulg.ac.be/enquete/index.php/219425?lang=fr and its English translation is viewable in the Appendix.

A total of 245 producers completed our survey between November 2014 and January 2015.

The entire survey was composed of 127 questions where the answers were decomposed into 498 categorical and 44 quantitative variables.

The question 'Without taking into account your current farm, what is, according to you, the ideal future farm to ensure a revenue?" was proposed to the producers and they

must choose between short propositions on seven items: 1) intensive or extensive production; 2) specialised or. diversified activity (or activities); 3) farming strongly or weakly based on new technologies; 4) farm managed by an independent farmer or a group of managers; 5) family or employed workforce; 6) providing production for local or global markets; 7) providing standard or differentiated quality production. The modality “no opinion” was available for each IFF question. Counts were calculated for all modalities of these seven sub-questions.

The first step was to study if there were relationships between all modalities derived from the seven sub-questions asked. To achieve this objective, a multiple correspondence analysis (**MCA**) was carried out as the variables were categorical. For a MCA, the eigenvalue of the dimensions generated, named principal inertia, is a biased measure of the amount of information presented by a dimension (17). Corrected inertia rates were calculated, as described by Benzécri (18), to quantify the correct proportion of information of a dimension.

Classes were established to study the distribution of producers along the dimensions of the MCA. The interval between the 1% percentile and the 99% percentile of each dimension was divided equally into five classes. Then, the individuals per class were counted.

To exclude a group of producers with some characteristics if necessary, cluster analysis with the WARD method was used on the scores of the individuals on each dimension of the MCA. The WARD method is a hierarchical agglomerative method (19). The principle of this kind of method is to put initially the n individuals in n groups

and then to agglomerate the groups. The algorithm of WARD makes it in such a way that the gatherings induce the lowest decrease of R^2 at each step.

If a group of producers was excluded, its characteristics were previously studied against the remaining producers. The level of significance of the difference of the quantitative characteristics between the excluded and the remaining producers was studied thanks to general linear models. The level of significance of the difference of the proportions for each modalities of the qualitative characteristics between the excluded and the remaining producers was studied thanks to tests of proportions.

Characterisation of IFF choice

To describe the dairy producers in terms of their IFF, the scores on MCA dimensions were studied as a function of other variables extracted from the survey. This method was chosen instead of the creation of classes, possible with the Latent Class Analysis method or the Numerical Classification on the scores of MCA (Hierarchical Clustering on Principal Components). This choice was motivated by the wish to not put the producers in boxes but to study their position on a gradient between potential extreme models identified along the dimension.

The other variables extracted from the survey whose the relationships with the dimension are studied were distributed within several themes. These were the effect of past crises, problems encountered by the farmer, production factors, age of the farmer, breed of the cow, diversification of activities and alternative valorisation, regrouping between producers, consideration of mechanisation and robotisation on the farm, the reaction of the farmer to external factors, the considerations of farmers about environmental aspects, climatic hazards, ways to reach the ideal formation and field of formation. For categorical variables, the scores of MCA dimensions were modelled

using these variables as a fixed effect in a generalised linear model. Least squares means were estimated for the two-by-two comparisons using the Tukey test. The level of significance of those differences was assessed based on the *P*-value of the test. For quantitative variables, Pearson correlation coefficients were calculated between the scores of MCA dimensions and these variables. Their corresponding *P*-values were estimated to observe if the correlation values were significantly different from 0.

To observe if dairy producers presented the farming characteristics they considered to be ideal at the moment of survey, absolute frequencies (counts) were calculated as a function of each ideal future farm characteristic and of the answer to the question which corresponds to this characteristic for the current situation (Table 1). Moreover, the percentage of “unhappy” producers was calculated as the ratio between the producers not currently in the situation that they consider as ideal and the total number of producers.

All editing and statistical analyses were carried out using SAS software (version 9.4., SAS Inst. Inc., Cary, NC, USA).

Table 1. Absolute frequencies (counts) of producers as a function of their answer to the ideal future farm characteristic and the corresponding characteristic for the current situation and percentage of "unhappy" producers (*i.e.*, percentage of producers not currently in the situation that they consider to be ideal) (N = 245)

[1] Frequency in grey box corresponds to producers not currently in the situation that they consider as ideal regarding this characteristic

		**Corresponding characteristic for the current situation**		**% of "unhappy" producers**
		>2 cows per hectare of grass	<2 cows per hectare of grass	
**Ideal future farm characteristic**	Intensive	38	66[1]	50%
	Extensive	22	51	
		Only dairy production activity	Presence of activities other than dairy production	
	Specialised	46	59	37%
	Diversified	23	93	
		Presence of milking robot or agricultural equipment for a better technicality	Absence of milking robot or agricultural equipment for a better technicality	
	Strongly based on new technologies	33	52	37%
	Weakly based on new technologies	16	85	
		>1 chief operating officer or associates	1 chief operating officer	
	Managed by a group of managers	20	25	42%
	Managed by an independent farmer	68	108	
		Presence of workers (i.e., external person to family working on the farm)	No workers	
	With family workforce	17	195	10%
	With employed workforce	7	6	
	Providing dairy production for local *vs*. global market	No corresponding characteristic		
	Providing standard *vs*. differentiated quality dairy production	No corresponding characteristic		

Results and discussion

Data representativeness

The sample set of 245 producers represented 6.1% of the dairy producers in Wallonia (about 4,000 dairy producers in 2015 and 3,500 in 2017 (4)). The density of dairy farms throughout Wallonia was well represented in the sample, with a higher answer rate in the provinces more populated with dairy farms. More answers were obtained in the east part of Wallonia, where a higher density of dairy farms exists due to the grazing landscape that is particularly suitable for dairy production. Wallonia is a highly heterogeneous region with regard to soil and geological characteristics (20).

Dairy producers of the survey declared a mean of 79 cows and 86ha. Dairy production was their unique activity for 33% of them. The mean number of dairy cows per Walloon dairy farm was 52.9 cows in 2015 (21). No regional statistic exists on the mean agricultural area of all producers that perform a dairy activity. The mean agricultural area of specialised dairy farms and of all kind of farms taken together equated to 61.98 ha and 55.8 ha, respectively (21). So, the producers surveyed tended to have bigger farms regarding herd size and agricultural area than the average Walloon farm that have dairy activity.

What is the perception of dairy producers of their ideal future farm?

Univariate approach

As mentioned previously, the first aim of this study was to highlight the perceptions of Walloon dairy producers of their ideal farm, just before the end of the milk quota. This was done through the answers to 7 sub-questions. Table 2 shows the frequency for each modality of those questions.

Table 2 Percentages of responses to the seven questions about the ideal future farm (N = 245)

**Question**<br>Without taking into account your current farm, what is, according to you, the ideal future [illegible] to ensure a revenue?”	**Proposition**	**Percentage (%)**
Intensive *vs*. extensive	Intensive	43
	Extensive	30
	No opinion	27
Specialised *vs*. diversified	Specialised	43
	Diversified	47
	No opinion	10
Strongly *vs*. weakly based on new technologies	Strongly	35
	Weakly	41
	No opinion	24
Managed by an independent farmer *vs.* a group of managers	Independent farmer	72
	Group of managers	18
	No opinion	10
Family *vs*. employed workforce	Family	87
	Employed	5
	No opinion	8
Providing dairy production for local *vs*. global market	Global	43
	Local	32
	No opinion	25
Providing standard *vs*. differentiated quality dairy production	Standard	38
	Differentiated quality	45
	No opinion	17

Contrasting opinions of dairy farmers were observed for almost all questions except for the type of management and the kind of workforce: 71.84% of the respondents wanted an independent farmer management, and 86.53% focused on a family workforce (Table 2). These results highlight a will in the southern part of Belgium to maintain the traditional structure of work organisation in the future, with family workforce and one director of operations. More globally in the world, dairy farms are still mostly owned and managed by a family structure, whatever the degree of

development of the country (22,23). The choice of producers to work by themselves and not to deal with workers (i.e., an external person to the family employed on the farm) was noted in other studies. For example in Spain Gonzalez and Gomez (24) observed, when asking 3,370 farmers for their definition of a farmer, that more than half of them chose labourer and 12% chose businessman. In the USA in 1988, Mooney presented the fact that farmers had a particular status, being workers and employing other workers (25).

From Table 2, it is interesting to note that the highest percentages of abstention were observed for the questions about intensive *vs*. extensive, strongly *vs*. weakly based on new technologies, and providing DP for local *vs*. global markets. These results show that a quite significant proportion of the respondents did not take a position on these directions for the evolution of dairy farms.

Multivariate approach

To study the relationships between the answers given by the respondents to all questions about IFF, a MCA was performed as the related variables were categorical (Table 2). The percentage of principal inertia of the dimensions 1 and 2 of MCA were 16.75% and 12.38%, respectively (Fig 1). The value of corrected inertia for the two first dimensions reached 72.7% and 21.5% respectively, gathering almost 95% of the information.

Fig 1. Representation of the modalities in the multiple correspondence analysis first factorial plan. Values of principal inertia reached 16.75% and 12.38%. Values of corrected inertia reached 72.7% and 21.5%.

The first dimension of MCA showed positive relationships with the modalities no opinion of each characteristic and negative relationships with all the modalities with an

opinion. Thus, the first dimension of the MCA allowed differentiation between the producers who did not give their opinion concerning characteristics of IFF and the producers who did (Fig 1). Cluster analysis was used to isolate the group of producers with a lot of ‘no opinion’ answers to the seven questions: this formed the first separation of classes of the analysis, dividing the “no-opinion” producers (15%) from the others (85%). The no opinion producers cluster (N = 38) was removed from the analysis to avoid potential bias coming from farmers who did not have a clear vision of their IFF.

Detailed information about this group is available in Tables 3 and 4. They tended to be older farmers (45-54 years), who came from Liège, which is a historic dairy region (Table 3). Even if it is not significant (P = 0.27), they are less likely to have other animal production on the farm (Table 3). Percentages of grass and corn silage observed for this group highlighted a same way of feeding as the complete sample (Table 4). Even if these differences are not significantly different (P = 0.20, P = 0.59, P = 0.33), the more represented single breed and the lower number of cows but with the higher milk delivery quota of the no-opinion producers tended to express quite technical and high performing producers in this group. They were more, even if not significant, to plan not to keep constant their production (Table 3). Even if not significant (P = 0.32), they declared more investment in the next five years than the complete sample, but no difference of investment was observed since 2009. They seemed to be people who have been dairy producers for a long time. We could assume that their farms had good economic performances and did not lead them to think about evolution in response to a great change (*i.e.* the quota removal).

Table 3 Percentages of producers as a function of modalities of categorical variables for the no-opinion producers and the complete sample.

%	Complete sample N = 245	No-opinion producers N = 38	Producers with an opinion N = 207	*P* No-opinion *vs.* with opinion
**Age (years)**				
0-34	18	16	19	0.64
35-44	31	32	31	0.98
45-54	37	47	35	0.15
55-64	13	6	15	0.027
**Geographical situation**				
Walloon Brabant	6	2	6	0.24
Hainaut	32	34	31	0.74
Liège	34	45	32	0.14
Luxembourg	10	5	11	0.20
Namur	19	13	20	0.28
**Importance of dairy activity**				
Unique activity	33	34	32	0.83
Preponderant activity	65	61	65	0.59
Secondary activity	3	5	2	0.45
**Herd breed**				
Single breed	33	42	31	0.20
Multi-breed	67	58	69	0.20
**Other animal production**				
Yes	45	37	46	0.27
No	55	63	54	0.27
**Milk production evolution in the next 5 years**				
Decrease	2	0	2	0.043
Constant	54	63	52	0.20
Increase	38	32	40	0.33
Stop	6	5	6	0.79
**Agricultural area investment since 2009**				
Yes	46	47	46	0.88
No	54	53	54	0.88
**Agricultural area investment in the next five years**				
Yes	57	64	55	0.27
No	43	36	45	0.27

Table 4: Means of quantitative variables for no-opinion producers and the complete sample

	Complete sample N = 245	No-opinion producers N = 38	Producers with an opinion N = 207	*P* No-opinion *vs.* with opinion
Agricultural area (ha)	86	87	85	0.80
Percentage of corn silage	15	14	15	0.67
Percentage of grass	61	60	60	0.76
Milk delivery quota (l)	558743	632880	545133	0.33
Number of cows	79	73	81	0.59

The second dimension of the MCA showed positive relationships with some modalities of the IFF characteristic and negative relationships with their opposite. Thus, this dimension seems to highlight the wishes of dairy farmers about their IFF, for those who took a position on this question. More precisely, this axis showed a gradation of question modalities and proximity between several characteristics. The second dimension of the MCA was the most interesting for highlighting the wishes of dairy farmers about their IFF, for those who took a position on this question. This axis showed a gradation of question modalities and proximity between several characteristics. This dimension led to the identification of two extreme tendencies (Fig 1); the modalities of familial workforce, independent farmer management and management by a group of farmers were near to zero on this axis (Fig 1). This means that the small proportion of producers supporting group management was distributed between the two extreme tendencies observed. The position of the modalities of familial workforce and independent farmer at the middle of the second dimension illustrated the fact that these modalities were chosen by producers from the two tendencies identified. The small proportion of producers choosing an employed workforce was positioned at the top of the second dimension (Fig 1).

The first tendency, related to high scores on the second MCA dimension, corresponds to IFF with the following characteristics: global market, standard milk, intensive system, employed workforce, specialised and strongly based on new technologies. Other authors have observed the same relations. From a trial of 458 French dairy farms, Hostiou *et al.* (26) highlighted a profile of farmers which simultaneously gathered high equipment, intensification and workers. From a trial of 3,370 producers of all sectors in Spain, Gonzalez and Gomez Benito (24) collated the characteristics of large holdings, market-orientated farming and management of workers. Cournut *et al.* (27)

highlighted different ways of evolving dairy farming in France, characterised by workers, mechanisation and high equipment. This tendency in dairy farming systems is explained by the evolution of the dairy system (6). The increased competition in the dairy market caused by the creation of the open European market, as well as the wish of consumers to have structures that gather all the food supplies in one place (*i.e.* a supermarket) led to the concentration of dairy processing in a few big firms (11). These firms were better placed to develop because they could control their collection costs, benefit from scale economies and were able to deliver to supermarkets with regularity in quantity and with a standard quality (9). This state and the world market have conditioned milk prices for the producers. Increasing production, thanks to more cows or higher productivity, is a possible way to stay profitable, considering the undergone milk price (5,11). To achieve profitability, an elevated production of milk per cow and an increase of cows on the farm are reached (11). Moreover, this increase in milk production at farm level was also forced by the orientated production Common Agricultural Policy (**CAP**) primes, although CAP has limited help for the dairy sector. Therefore, all of these characteristics intensify the dairy farming system. Intensification was defined by Garcia-Martinez *et al.* (28) as the maximisation of the rarest factor, traditionally the agricultural area. The increase in DP per unit of agricultural area was possible thanks to intensive production of forage and purchase of inputs that are produced where production costs were the lowest, to balance the ration and to increase the production per cow, or the number of cows reared on a hectare of agricultural area and therefore DP per unit of agricultural area at the level of the farm (9,11). This intensification led to more specialised farms with more dairy cows and their entire workforce directed to this specialisation (9). The enlargement of farms required a higher work rate; this was surmounted thanks to equipment and new technologies and

to increased human workforce: collective organisation, subcontracting to private firms and employment of workers (9).

The second tendency, contrary to the first tendency, was characterised by high negative scores on the second MCA dimension. This axis was represented by the following modalities: weakly based on new technologies, diversified, differentiated quality milk, local market and extensive system (Fig 1). This reflects another form of dairy farming. This form is favoured by a constant increase in input prices, combined with a growing demand from consumers for high quality and local-based products (9). These dairy producers choose to work with greater self-sufficiency to be less dependent on the undergone input prices (9). The “localisation” of the production demanded by consumers was executed thanks to this more locally-produced forage and fewer inputs from outside (5). This return to self-sufficiency led to more extensive farming (5). The production induced was also often quality-differentiated and dedicated to local markets (9). Cournut *et al.* (9) showed in their study that this kind of dairy farming is chosen by a minority of farms, which are still diversified.

This gradation with two kinds of models at the extremities of the second MCA dimension was also described in other studies (5,6,9,11,29–31). They were named globalisation *vs*. territorialisation by Cournut *et al.* (9), or globalisation *vs*. localisation by Napoleone *et al.* (11). Lebacq (6) identified a “dualisation of dairy farming systems between ‘a mainstream model’ focusing on an increasing farm size, production intensity and specialisation and alternative models involving initiatives deviating from this trend and constituting niche developments (niches = minor elements, hardly sustainable against the mainstream model)”. Thanks to a survey answered by 180 producers of all sectors in 2007 in France, concerning the evolution of their farms and their aspirations, Dockès *et al.* (30) also highlighted a major tendency towards the

enlargement, professionalisation and specialisation of farms, but those authors also mentioned that other farms wanted to develop diversified structures, orientated towards the requests of society, processing and farm accommodation.

How do dairy producers distribute themselves between the ideal future farms highlighted?

The present study showed a bifurcation and quantified two ways: 46% *vs*. 26% of producers having high positive and high negative scores respectively on the second dimension (Fig 2). Verhees *et al.* (15) quantified producers as a function of their strategies of development, but solely regarding specialisation *vs*. diversification of their activity, 54.3% *vs* 15.1% respectively. The bifurcation phenomenon is also observed in the organic sector. Two models appeared: organic agriculture realised by historic actors and the other driven by the agribusiness to answer to a increasing organic demand (32–34).

Fig 2. Distribution of the producers along the second dimension (the dotted line represents the mean score on the second dimension of the producers)(N = 207)

How do farmers decide on their ideal future farm?

To study the relationships between the different IFF, the reasons for these and other interesting technico-economic information, the second dimension was considered as a gradient (**IFFg**) interpreted at the extremities as global-based intensive producers (GBI: high positive scores) and local-based extensive producers (LBE: high negative scores). The choice to work with a gradient rather than a clear separation of the two tendencies was motivated by the will to not put dairy producers into boxes. The mean of the scores of the second MCA dimension was –0.012 with a SD of 0.053. Minimal and maximal values were –1.09 and 0.92, respectively.

Based on the interpretation of IFFg, a significant negative correlation indicates a higher relationship with the dairy producers desiring a LBE model. By opposition, a significant positive correlation means a higher link with the dairy producers desiring a GBI model. Tables 5, 7 and 8 give the results of generalised linear models where the categorical variables were introduced separately as a fixed effect in the model. Significantly lower estimates of IFFg for a specific modality of the considered categorical variable depicts a tendency of producers desiring a LBE model to choose this modality, while significantly higher estimates of IFFg means a tendency of producers wanting a GBI model to choose this modality. These analyses were conducted on the producers who have an opinion (N = 207). The following paragraphs will summarise the potential reasons driving the choice of IFF made by the Walloon dairy farmers.

Effect of past crisis on perceptions of the ideal future farm The producers that were impacted by past crises wished more for a LBE model (estimate = –0.17, Table 5). This could be related to the suffering involved in the crisis and the wish to apply solutions in order to not repeat this situation: revenue from diversified activities, other outlets for the milk production sold (*i.e.* local market characteristic) and/or self-sufficiency to be less dependent on purchased feed (*i.e.* extensive farm characteristic). This is in agreement with a past finding (35). We observed a decrease in intensification in 2012 which was the year of a dairy economic crisis mainly related to an increase in the price of inputs.

Table 5 Value and level of significance of the difference in the ideal future farm gradient as a function of modalities of categorical variables: Reasons (N = 207)

Means with different letters are significantly different.

Categorical variable	Modality and estimate	*P*
**Past crisis**		

Presence of deep modifications after crisis	No<br>0.031		Yes<br>–0.17			0.025
**Workload**						
Degree of arduousness	Not arduous<br>–0.11[b]	Arduous<br>–0.092[b]	Highly arduous<br>0.15[a]			0.0043
Member of an agricultural replacement service	Yes<br>0.058		No<br>–0.0801			0.059
Worker engagement: help for workload and administrative aspects	Already implemented<br>0.024[ab]	To implement in the future<br>0.13[a]	Not interested<br>–0.040[b]			0.25
**Production factors**						
Milk production evolution for 5 years	Decrease<br>–0.21	Constant<br>–0.13	Increase<br>0.052			0.036
Agricultural area investment since 2009	No<br>–0.14		Yes<br>0.14			0.0002
Investment (no agricultural area) since 2009	No<br>–0.19		Yes<br>0.015			0.055
Agricultural area investment in 5 years	No<br>–0.0909		Yes<br>0.046			0.073
Geographical situation	Brabant Walloon<br>0.11	Liège<br>0.12	Namur<br>0.055	Luxem-bourg<br>–0.013	Hainaut<br>–0.099	0.51
**Age**						
Age (years)	0-34<br>–0.086	35-44<br>0.0105	45-54<br>0.0049	55-64<br>0.0502		0.67
**Diversification and alternative valorisation**						
Presence of other animal production	Yes<br>–0.093		No<br>0.0603			0.037
Dairy processing and direct sales: sector developed if supported	Yes<br>–0.33		No<br>0.11			<0.001
Processing and direct sales (except dairy): sector developed if supported	Yes<br>–0.39		No<br>0.013			0.0096
HORECA, tourism and teaching activity to develop even if sustained	Yes<br>–0.18		No<br>0.018			0.055
Concern for diversification	Yes<br>–0.23		No<br>0.17			<0.001
Alternative chain for milk production valorisation	Yes<br>–0.49		No<br>0.036			<0.001
Alternative chain for “other than dairy” activity	Yes<br>–0.56		No<br>–0.0087			0.0017
Increase of “other than dairy” activity without investment	Yes<br>–0.42		No<br>–0.013			0.012
No activity to develop even if sustained	Yes<br>0.27		No<br>–0.10			<0.001
Increase of added value in farms: advantage of diversification and transformation	Yes<br>–0.097		No<br>0.11			0.0047
Link between producers and consumers: advantage of diversification and transformation	Yes<br>–0.14		No<br>0.066			0.0064
Conservation of farms in the region: advantage of diversification and transformation	Yes<br>–0.16		No<br>0.028			0.037
Financial, decisional and technical autonomy: advantage of diversification and transformation	Yes<br>–0.27		No<br>0.030			0.005
Consumer loyalty: limit to diversification and transformation	Yes<br>0.17		No<br>–0.047			0.028

Regulatory constraints (hygiene, *etc.*): limit to diversification and transformation	Yes<br>–0.080	No<br>0.055			0.065
Size of investments: limit to transformation and diversification	Yes<br>–0.14	No<br>0.0401			0.030
No constraints for transformation and diversification	Yes<br>–0.093	No<br>0.042			0.052
No advantage of diversification and transformation	Yes<br>0.27	No<br>–0.066			0.0006
**Breed**					
Composition of the herd	Single breed<br>0.18	Multi-breed<br>–0.095			0.0005
	Pure-bred<br>0.0058	Dual purpose breed:<br>–0.19			0.0023
**Regrouping**					
Advantage of fiscal and administrative aspects: advantage of grouping	Yes<br>–0.16	No<br>0.023			0.050
Development of a joint project: advantage of grouping	Yes<br>–0.15	No<br>0.020			0.072
Better marketing of the products: advantage of grouping	Yes<br>–0.38	No<br>0.0063			0.030
**Mechanisation and robotisation**					
Mechanisation, robotisation: help with workload and administrative aspects	Into effect<br>–0.031[ab]	Not interested<br>–0.094[b]	To activate<br>0.12[a]		0.041
**Reaction to external factors**					
Arduousness of the economic uncertainty of input price	No<br>–0.19	Yes<br>0.0403			0.0089
Will of a dairy factory imposing production limits	Yes<br>–0.23[c]	No<br>0.25[a]	Not important<br>0.0088[b]		<0.001
Evolution of milk production during crises (2009, 2012)	Decrease<br>–0.17[b]	No variation<br>–0.092[b]	Increase<br>0.21[a]		0.0006
Ideal size of the dairy factory	Small<br>–0.52[c]	Medium<br>–0.11[b]	Large<br>0.42[a]	Not important<br>–0.026[b]	<0.001

Workload Workload seems to be less bearable for producers desiring a GBI model (estimate = 0.15, Table 5; $R_{\text{workforce constraint}}$ = 0.22, P = 0.002). Producers wishing for a GBI model were also more likely to be members of an agricultural replacement service (estimate = 0.058, Table 5) and showed a tendency to be more interested in employment of workers (estimate = 0.13, $P_{\text{worker engagement to implement } vs. \text{ not interested}}$= 0.11, Table 5). The choice of GBI model could be explained by this current workload, involving the need for an increase of revenue. So, the solution considered could be higher milk production and the breeding of more cows rather than diversification of activities and self-valorisation activity, the development of which requires a lot of time.

Samson *et al.* (36) confirmed this in the Netherlands by highlighting a nearly significant effect of labour productivity on the DP increase strategy.

Production factors The size of agricultural area, the milk delivery quota, the number of cows and the percentage of corn silage currently observed in the farming system showed significant and positive correlations with IFFg (R = 0.15, 0.36, 0.18 and 0.24; *P* = 0.033, <0.001, 0.0099, 0.0002 respectively). So, dairy producers choose their IFF partly as a function of their current production factors. This is expected as a higher number of hectares, cows and litres means a higher capacity of the dairy installation, of the material and so the possibility of a more preponderant dairy activity. The higher percentage of corn silage also reflected the possibility to seed corn silage, allowing the intensification of production as required within a GBI model. Similar relationships between characteristics of the farm and current or desired models of farming were observed by others. For Central and Eastern Europe, Verhees *et al.* (15) showed that land was the most important factor in developing a specific farming strategy. In France, Hostiou *et al.* (26) observed that intensified farms with higher technology equipment sometimes employed more workers, and were the farms with significantly higher agricultural area, percentage of corn silage, number of cows and milk quota. In the Netherlands, Samson *et al.* (36) showed that production intensity, number of cows, modernity of technology and availability of land were important factors in DP increase strategies.

In contrast, producers with lower production factors can consider rarely more enlargement and therefore think differently about the enhancement of their revenue: better valorisation of quality differentiated milk, other activities on the farm, self valorisation, the LBE model. Samson *et al.* (36) showed that lower stable capacity varies inversely to a DP increase strategy, which is rather a GBI tendency.

The findings of the current study, as confirmed by previous researchers, showed that producers work within a tightly constrained and regulated environment limiting their ability to determine the future of their farm according to their personal desires. This statement was also concluded by Mc Elwee *et al.* (37) and Methorst *et al.* (38). In the Netherlands, Keizer and Emvalomatis (39) and Groeneveld *et al.* (40) showed that bigger farms are more likely to increase than other farms.

However, based on the quite low values of the correlations obtained between the agricultural area and the number of cows, we can consider that this situation must be nuanced and that the IFF chosen also depends on the opinions of the dairy producer, not taking into account the current situation of his farm. This statement is reinforced by the fact that the correlation of percentage of meadow with IFFg was not significantly different to 0 ($R = -0.097$, $P > 0.1$). Also, the impact of the provinces of the Walloon Region, which present different geographical and soil characteristics, on IFFg were not significantly different ($P = 0.51$, Table 5).

Moreover the significant relations between IFFg and milk production evolution for five years (Table 5; $R_{\text{quantity of milk variation}} = 0.30$, $P < 0.001$), investment for and in five years (Table 5) support the assumption that the IFF chosen depends greatly on the mentality of the producers.

In their study, Methorst *et al.* (13) proved the heterogeneity of farm developments of producers facing the same socio-material context, showing the importance of the mentality of the producers in their decisions. Authors speak about shared values, norms, ways they see themselves or would like to be seen by producers, views, capacities and their perceptions of opportunities and any room for manoeuvre, skills, motives, entrepreneurship, goals and strategies (12,13,36,38,41) as factors which influence farm development. Samson *et al.* (36) discussed experimental economics,

which are economics where psychology and biology, which explain human behaviours, are added to better explain the development of enterprises. The consideration of more than just economic aspects permits them to reduce the error of their model for predicting DP increase strategies (36).

Age Age of the producer seems not to condition the desired IFF (Table 5). An IFF could be chosen because of either the new ideas of young producers or the experience of older producers. If mentality seems to influence IFF choice, it is not linked to age. The two kinds of IFF could be an answer to both innovation and problems encountered during a long career. Samson *et al.* (36) also studied age as a reflection of the farmers' values, goals and strategies, and showed no relationship with DP increase, which is rather a GBI characteristic. On the contrary, on the basis of data from 11 countries of the European Union, Weltin *et al.* (42) observed an effect of age on the tendency towards diversification, which is rather a LBE tendency.

Diversification and alternative valorisation The results obtained in this study showed a link between the diversification mentality and the choice of LBE model. Significant negative estimates or correlations were observed for the following variables related to diversification: the presence of other animal production (estimate = –0.093, Table 5); the direct selling milk quota (R = –0.17, P = 0.016); dairy or no dairy processing and direct sales (estimates = –0.33 and –0.39, Table 5); the development of HORECA activities, tourism and teaching (estimate = –0.18, Table 5); the concern for diversification (estimate = –0.23, Table 5); alternative chain for milk and other than milk production valorisation (estimates = –0.49 and –0.56, Table 5) and the increase of "other than dairy" activity without investment (estimate = –0.42, Table 5). Conversely, producers desiring a GBI model were more likely to choose the item "no activity to develop if supported", suggesting the unique principal activity way of thinking

of producers aiming for a GBI model (estimate = 0.27, Table 5). Samson *et al.* (36) confirmed this tendency and showed that the presence of diversified activities evolved inversely to the increase of milk production. In this study, we observed potential explanations to support to this fact. Producers wishing for a LBE model considered self-valorisation and diversification as solutions to the current situation to enhance revenue due to the creation of added value (estimate = –0.097, Table 5). They thought that diversification and transformation allowed financial, decisional and technical autonomy (estimate = –0.27, Table 5) and were confident in consumer loyalty (estimate = –0.047, Table 5). They considered relations with consumers as an opportunity and not a threat, unlike producers desiring a GBI model (estimate = 0.17, Table 5). One reason GBI model producers gave against self-valorisation and diversification seemed to be the lack of trust in consumers and therefore the outlets. They frequently saw no advantage to self-valorisation and diversification (estimate = 0.27, Table 5). The relation to the consumer was also studied by Verhees *et al.* (15). They observed that consumer orientation was more often declared as an opportunity by the profiles of producers considering strategies similar to LBE. The positive impact of diversified activities on autonomy was also shown by Bergevoet *et al.* (12). They mentioned that proponents of the “extra source of income” model (closest to the LBE model) were more able to declare that they can increase the sales-price of their milk. Producers wishing for a LBE model were also likely to find no constraints to transformation and diversification (estimate = –0.093, Table 5). The only limits to diversification and transformation highlighted by producers wanting a LBE model were regulatory constraints (estimate = –0.080, Table 5) and the size of investments (estimate = –0.14, Table 5). As a consequence of these considerations, producers wanting a LBE model felt that they were more able to meet society’s expectations regarding local and

artisanal products (R = –0.22, *P* = 0.0016) and the desire for a familial structure (R = –0.12; *P* = 0.084).

Breed to produce milk Producers wanting a LBE model are more open to breeding a dual-purpose herd (estimate = –0.19, Table 5), which permits them to diversify their production: milk and meat. Producers wishing for a GBI model target a single, more specialised breed (estimate = 0.18, Table 5) which could offer more homogeneous management of the herd. The link between mentality, observed through the choice of breed(s), and the choice of IFF is once more highlighted.

Regrouping Producers tending towards the LBE model were more likely to promote regrouping for its advantages regarding fiscal and administrative aspects, the development of a joint project and the marketing of the products (estimates = –0.16; –0.15; –0.38, Table 5). The importance of mentality for the choice of IFF has been shown. A mentality of cooperation, as a solution to enhance their quality of life and revenue, tends to be shared between producers desiring a LBE model.

Mechanisation and robotisation We observed that the wish of technology of producers tending towards GBI model can be explained by the fact that they considered it as help for workload (estimate = 0.12, Table 5). It can be assumed that the solution considered by them is to keep the same activity or increase it with help from machines. In southern France, Dufour *et al.* (43) observed the propensity of farmers with workers, close to the GBI model, to prioritise investment in equipment. Verhees *et al.* (15) observed that better management, including new technologies, was more cited as an objective for producers whose strategy profiles were more similar to the GBI than LBE models.

Reaction to external factors Reactions of dairy producers to factors external to their decision-making power tend to be different as a function of their choice of IFF, showing once more a different mentality of the producers. Producers wanting a LBE model tend to show themselves to be more independent from the external economic actors: from the input producing companies (estimate = –0.19, Table 5) and from the market and factories, rejecting contracts which would link them to it (R = –0.13, Table 6). When their opinion about dairy factories was surveyed, producers desiring a LBE model preferred small or medium units with production limits (estimates = –0.52; –0.11; –0.23, Table 5), as before, which means regulation of the dairy offerings on the market. Producers wishing for a GBI model direct themselves to big units of processing without production limits (estimates = 0.42; 0.25, Table 5) and so more turned towards world markets. They recognise the freedom in regarding DP as an asset of quota removal (R = 0.23, Table 6). The reaction regarding the quantity of production was not similar during a crisis, producers wanting a LBE model tended to maintain or decrease their production (estimates = –0.17; –0.092, Table 5), whereas producers desiring a GBI model tended to increase production (estimate = 0.21, Table 5). The latter wanted to keep revenues constant with more litres produced when the price decreased, while the others controlled or decreased production when the gross margin per litre decreased. This can be due to a deliberate choice to decrease milk production or a decision to decrease the variable costs causing a decrease in milk production. These results can express a fear of producers tending toward the LBE model in considering world markets, contrary to producers tending towards the GBI model who have decided to work with this kind of market. Verhees *et al.* (15) observed that producers projecting strategies similar to the LBE model consider the market more as a threat than producers projecting strategies similar to the GBI model. Hansson *et al.* (44) and Weltin

et al. (14) explained that this uncertainty and risk perception can explain the choice of diversification, which is a part of the strategy of the LBE model.

Couzy and Dockès (7) demonstrated different profiles of farmers and observed the entrepreneurship mentality of each one, which highlights similar tendencies to those presented here. Several profiles showed strong entrepreneurship but which was expressed differently to here. A category of farmers showed entrepreneurship by their wish for autonomy of decision in their management; they will keep a working approach close to the conventional one but with a modernist vision, always adapting to the market. They want to keep freedom in the classical framework. In 1988, Mooney described the split personality of producers: they are independent people, making their own decisions regarding their way of working and their investments but at the same time are people dependent on different processing actors and banks (45). Another category of farmers showed entrepreneurship by their wish to develop an original idea, away from preexisting systems, a project in line with their conviction to be freer from the existing system [5].

Samson *et al.* (36) and Methorst *et al.* (13) reported that decisions of producers cannot be reduced to only economic aspects: this includes policies and market conditions but also their way of thinking about them.

Table 6 Correlations (R) between the ideal future farm gradient and quantitative variables (N = 207)

*producers declaring no calling of replacement services were removed from this analysis

Quantitative variable	R	*P*
**Reaction to external factors**		
Contract means dairy production more integrated to dairy factories: level of agreement	–0.13	0.076
Quota removal means more flexibility concerning production: level of agreement	0.23	0.0014
**Considerations of the environmental aspects**		
Degree of the constraint: livestock manure application	0.16	0.022
Facility to answer to society's expectations: environmentally friendly agricultural practices	–0.15	0.027
Agricultural activity is important for rurality of villages: level of agreement	–0.23	0.0011
Agricultural activity is important for conservation of permanent grasslands: level of agreement	–0.27	<0.001
Agricultural activity is important for biodiversity: level of agreement	–0.18	0.0101
Agricultural activity is important for planting and maintenance of hedges: level of agreement	–0.28	<0.001
Importance of answering society's expectations for the revenue of the dairy producers: level of agreement	–0.11	0.11
Ease of answering society's expectations: landscape and territory maintenance: level of agreement	–0.19	0.0065
**Needs: formation method**		
*Frequency of calling replacement services for meeting and formations (N = 104)	0.21	0.066

How do environmental aspects factor into IFF decisions?

The environmental aspects related to the desired IFF were studied as awareness of the environmental impact of breeding has become an important issue of our time.

Producers tending toward the GBI model seemed to work with a higher livestock manure application pressure (R = 0.16, Table 6) and therefore are already more likely to work in an intensified dairy system, which can have a greater impact on the environment. Samson *et al.* (36) showed a tendency toward manure production surplus by producers with increasing DP, which is rather a GBI characteristic.

Results of practices that are in accordance with the environment: measurement of the grass height, forage mixture with leguminous plants, use of a field notebook (estimates = –0.27; –0.11; –0.074, Table 7) showed a stronger interest from producers wanting a LBE model.

Table 7 Value and level of significance for the difference in the ideal future farm gradient as a function of modalities of categorical variables: Environmental aspects (N = 207)

Means with different letters are significantly different.

Categorical variable	Modality and estimate		*P*
**Considerations of environmental aspects**			
Measurement of the grass height: optimisation practice	Yes<br>–0.27	No<br>0.0083	0.059
Forage mixture with leguminous plants: optimisation practice	Yes<br>–0.11	No<br>0.083	0.0088
Field notebook: optimisation practice	Yes<br>–0.074	No<br>0.061	0.065
**Climatic hazard**			
Increase of concentrate distribution: strategy to confront climatic hazards	Yes<br>0.22	No<br>–0.036	0.036
Decrease of the herd: strategy to confront climatic hazards	Yes<br>–0.25	No<br>0.014	0.037
Food self-sufficiency: cause for maintaining constant or decreased milk production	Yes<br>–0.17	No<br>0.0073	0.14

Besides these, all the significant negative correlations between IFFg and the levels of agreement with an agricultural area are important for the rurality of villages (R = –0.23, Table 6), for conservation of permanent grasslands (R = –0.27, Table 6), for biodiversity (R = –0.18, Table 6) and for hedges (R = –0.28, Table 6) showed the importance of the environment in the dairy activity of producers wanting a LBE model. It can be assumed that both LBE producers and GBI producers have concerns for the environment but in different ways. These results showed that LBE producers are more willing to employ the benefits of ecosystem services, which is observable in this database. Moreover, they found it easy to realise environmentally friendly agricultural practices, as asked for by society (R = –0.15, Table 6) and which are important to answer to society's expectations to guarantee their revenue (R = –0.11, Table 6).

Bergevoet *et al.* (12) had a considerably more consistent opinion. The "extra-source of income" profile producers (showing similarities with the LBE model) were more likely to declare that in their decision-making they take the environment into consideration,

even if it lowers profit. The “large and modern farm” profile producers do not mention their will to adopt these initiatives.

Climatic hazard Facing feed shortages due to unfavourable climatic conditions, producers tending toward GBI and LBE seem not to have the same way of thinking; GBI producers intend to buy high nutritional feed to balance shortages (estimate = 0.22, Table 7) and LBE producers are going to decrease the number of cows (estimate = –0.25, Table 7) and ensure their feed autonomy (estimate = –0.17, Table 7).

How do farmers' ideal future farm compare to their current farming systems?

The current situation of dairy producers was compared to their preferred IFF (Table 1). Except for the type of workforce, quite high percentages of “unhappy” producers were observed for the farm characteristics, between 37 to 50%. This suggested that not all producers work as they would like to. The same comparison was not found in the literature, to our knowledge.

As dairy producers do not work in a way that they consider to be ideal, it is interesting to study the gaps to fill in order to reach their ideal system and so, amongst others, their needs. The study of the requirements to reach the IFF, including ways to meet these needs and the area of the needs, can inform the stakeholders of the dairy sector about what must be developed to evolve into IFF.

Which paths and themes of training do dairy producers want in order to reach their desired ideal future farm?

Paths to formation As way to improve their skills, producers wanting GBI tended to favour consultancy (estimate = 0.17, Table 8) and commercial companies (estimate =

0.16, Table 8) and not days of study on other farms (estimate = 0.082, Table 8), meanwhile producers wanting LBE supported this latter possibility (estimate = –0.088, Table 8), a network of pilot farms (estimate = –0.13, Table 8) and the associate, non-market sector (estimate = –0.21, Table 8). Moreover, for help in technical choices, producers desiring LBE chose formation and study days (estimate = –0.15, Table 8) and producers' technical groups to implement in the future (estimate = –0.20, Table 8). The choices presented confirm the will for a non-market way to learn for producers wanting LBE, contrary to producers wishing for GBI.

As an information source, the agricultural press was commonly cited (N = 161, *i.e.* 78% of respondents), but producers desiring LBE tend to not want to inform themselves in this conventional way (estimate =–0.14, Table 8).

Producers wanting a GBI model tend to need more help to free them from their work in order to follow a formation (R = 0.21, Table 6)

Table 8 Value and level of significance of the difference in the ideal future farm gradient as a function of modalities of categorical variables: formations (N = 207)

Means with different letters are significantly different.

Categorical variable	Modality and estimate			P
**Needs: ways to learn formations**				
**Consultancy company: SFI* place	Yes<br>0.17		No:<br>–0.087	0.0017
**Study days on farm: SFI place	Yes<br>–0.088		No<br>0.082	0.026
**Network of pilot farms: SFI place	Yes<br>–0.13		No<br>0.056	0.025
**Associate, non-market sector SFI place development	Yes<br>–0.21		No<br>0.062	0.0023
**Commercial company: SFI place	Yes<br>0.16		No<br>–0.058	0.014
***Agricultural press: information source	Yes<br>0.025		No<br>–0.14	0.068
Formation and study day: help for technical choices	Already implemented<br>0.037[a]	To implement in the future<br>–0.15[b]	Not interested<br>0.035[a]	0.082

Producers technical groups: help for technical choices	Already implemented 0.020^{a}	To implement in the future $–0.20^{b}$	Not interested 0.11^{a}	0.0046
**Needs: domain formation**				
**Finance and management: requested formation	Yes 0.066		No –0.24	0.0007
**Processing and diversification: requested formation	Yes –0.18		No 0.089	0.0008
**Plant selection: requested formation	Yes 0.083		No –0.053	0.087
**Animal selection: requested formation	Yes 0.080		No –0.082	0.034
**Animal feeding: requested formation	Yes 0.03		No –0.14	0.073
**Administrative: requested formation	Yes 0.064		No –0.11	0.026
**Legal framework: requested formation	Yes 0.14		No –0.083	0.005
Request for advice: help for financial aspects	Already implemented 0.014^{a}	To implement in the future $–0.15^{b}$	Not interested 0.049^{a}	0.19

*SFI = study, formation and information

**producers declaring no will of formation were removed from this analysis

*** producers declaring no agricultural press as an information source were removed from this analysis

Formation domains The formation domains reflected the direction chosen by producers looking for LBE and the ways to reach it. They tend to want skills related to processing and diversification (estimate = –0.18, Table 8) and were likely to reject finance, management (estimate = –0.24, Table 8), administrative (estimate = –0.11, Table 8) and legal framework skills (estimate = –0.083, Table 8). For financial aspects producers wanting LBE tend to favour requests for advice from experts rather than self-formation (estimate = –0.15, $P_{\text{to implement } vs. \text{ not interested}}$ = 0.12, Table 8). They do not choose animal feeding (estimate = –0.14, Table 8) and selection formations (estimates = –0.053; –0.082, Table 8). This could suggest the will of the producers not to change their way of management and the level of quality of their herd but the method of valorisation of their production.

In contrast, producers desiring GBI tend to want to continue to enhance their vegetal and animal production (estimates = 0.083; 0.08, Table 8), to become more efficient

and enhance their revenue. Moreover they are more interested in legal aspects (estimate = 0.14, Table 8). Expansion and complexification of the GBI model of dairy farms wished for by these producers could be an explanation. Bergevoet *et al.* (12) also observed a will to be well informed about the legislation for the “modern and large farm” profile. This is not noted in their profile, which is close to the LBE model.

Two kinds of formation were identified and preferred by producers wanting LBE or GBI models. Bergevoet *et al.* (12) observed the will to innovate for the two profiles closest to LBE and GBI profiles of this study. Verhees *et al.* (15) observed that formation was the most important resource for dairy producers. The present research differentiated the formation desired as a function of IFF. Dufour *et al.* (43) defined, through a survey of 15 dairy farmers, three conceptions of the work: difficult, organisational and passionate. The passionate approach was accompanied by the desire for new knowledge which was, as observed here, either to learn about genetic selection or about processing and marketing of products.

Conclusions

In conclusion, the GBI tendency is two times more represented than the LBE tendency. Many reasons explain this choice of ideal farm. Past crises seem to cause farmers to desire the LBE model. A high workload seems to orientate respondents to the GBI model. The wish for the IFF is influenced by the current framework but is also a question of mentality. Production factors reached, breeds chosen for the herd, ways to react to factors external to the farm, consideration of diversification and alternative valorisation, regrouping and mechanisation and robotisation describe the producers' mentality and showed different relations with the IFF chosen. Moreover LBE and GBI producers may both have concern for the environment, but the approach to act for the environment by LBE producers, through concern for ecosystem services, is clearly highlighted in this study. These producers found it important to answer to society's expectations. Finally, as the current situation of farming is quite different to the ideal one, the learning needs were studied and two types of customer appeared in relation to their formation. We conclude that two kinds of dairy producers seem to appear, for different reasons, with different relations to the environment and asking for different formations.

Acknowledgments

I want to thank the organising committee of “Carrefour des Productions animales" for the supply of the data.

References

1. Mazoyer M, Roudart L. Histoire des agricultures du monde. Du néolithique à la crise contemporaine. Editions du Seuil; 1997. 1–705 p.

2. Lefèvre D. Des racines et des gènes. Une histoire mondiale de l'agriculture. Volume 1. Rue de l'Echiquier; 2018. 1–567 p.

3. European Economic Community. Treaty of Rome. 1957.

4. STATBEL. Exploitations agricoles et horticoles - plus de chiffres [Internet]. 2019. Available from: https://statbel.fgov.be/fr/themes/agriculture-peche/exploitations-agricoles-et-horticoles/plus

5. Havet A, Cournut S, Correa P, Madelrieux S, Poccard R, Corniaux C, et al. Tandem changes in livestock farming and feed systems since 1960: a study of six dairy-producing regions. Fourrages [Internet]. 2015;222:115–24. Available from: http://apps.webofknowledge.com/full_record.do?product=UA&search_mode=GeneralSearch&qid=3&SID=R2ZtQywoePOzfzM5uQW&page=16&doc=153&cacheurlFromRightClick=no

6. Lebacq T. La durabilité des exploitations laitières en Wallonie - Analyse de la diversité et voies de transition [Internet]. UCLouvain, Louvain-la-Neuve, Belgium; 2015. Available from: http://www.philagri.net/wp-content/uploads/2015/03/TL_redaction_these_20150302.pdf

7. Couzy C, Dockes A-C. Are farmers businesspeople? Highlighting transformations in the profession of farmers in France. Int J Entrep Small Bus [Internet]. 2008;6(3):407–20. Available from: http://www.inderscience.com/search/index.php?action=record&rec_id=19135&prevQuery=&ps=10&m=or

8. SPW Environnement Agriculture Ressources naturelles, Département de l'Etude du Milieu naturel, Direction de l'Analyse Économique et agricole. Agriculture Evolution de l'économie agricole et horticole de la Wallonie 2013-2014. 2013.

9. Cournut S, Rapey H, Nozières MO, Poccard-Chapuis, R., Corniaux C, Choisis JP, et al. Dynamics of livestock farming in extensive livestock farming territories : what processes are going on ? In: 10th European IFSA Symposium. Aarhus, Denmark; p. Unpaginated Ref. 26.

10. FAO. Livestock’s Long Shadow. Environ issues options. 2006;1.

11. Napoléone M, Boutonnet JP, Corniaux C, Alavoine-Mornas F, Barritaux V, Carvalho S, et al. Between local and global : which reconfiguration within Dairy Basins ? A comparative analysis from North & South case-studies. In: 10th European Social Science History Conference. Vienna, Austria; 2014. p. 1–12.

12. Bergevoet RHM, Ondersteijn CJM, Saatkamp HW, Van Woerkum CMJ, Huirne RBM. Entrepreneurial behaviour of dutch dairy farmers under a milk quota system: Goals, objectives and attitudes. Agric Syst. 2004;80(1):1–21.

13. Methorst RG (Ron., Roep D (Dirk), Verhees FJHM (Frans., Verstegen JAAM (Jos. Differences in farmers’ perception of opportunities for farm development. NJAS - Wageningen J Life Sci [Internet]. 2017;81:9–18. Available from: http://dx.doi.org/10.1016/j.njas.2017.02.001

14. Weltin M, Zasada I, Franke C, Piorr A, Raggi M, Viaggi D. Analysing behavioural differences of farm households: An example of income diversification strategies based on European farm survey data. Land use policy [Internet]. 2017;62:172–84. Available from: http://dx.doi.org/10.1016/j.landusepol.2016.11.041

15. Verhees F, Malak-Rawlikowska A, Stalgiene A, Kuipers A, Klopčič M. Dairy farmers’ business strategies in Central and Eastern Europe based on evidence from Lithuania, Poland and Slovenia. Ital J Anim Sci [Internet]. 2018;17(3):755–66. Available from: https://doi.org/10.1080/1828051X.2017.1422154

16. Salou T, van der Werf HMG, Levert F, Forslund A, Hercule J, Le Mouël C. Could EU dairy quota removal favour some dairy production systems over others? The case of French dairy production systems. Agric Syst [Internet]. 2017;153:1–10. Available from: http://dx.doi.org/10.1016/j.agsy.2017.01.004

17. Palm R. L’analyse à correspondances multiples : principes et application. Notes Stat d’informatique. 2007;

18. Benzécri JP. Sur le calcul des taux d’inertie dans l’analyse d’un questionnaire. Cah l’analyse des données. 1979;4:377–8.

19. Everitt BS, Landau S, Leese M, Stahl D. Cluster Analysis, 5th Edition. Shewhart WA, Wilks SS, editors. Wiley; 2011. 1–330 p.

20. Service Public de Wallonie. La Carte des Sols de Wallonie [Internet]. Available from: http://geoportail.wallonie.be/catalogue/ce3b6602-1c52-483f-9133-770009cdd02b.html

21. SPW Environnement Agriculture Ressources naturelles, Agricole, Département de l'Etude du Milieu naturel, Direction de l'Analyse Économique et agricole. Evolution de l'économie agricole et horticole de la Wallonie 2017. 2017.

22. De Haan C. Smallholder dairy production. In: Agriculture Investment Sourcebook. 2011. p. 169–73.

23. USDA/ERS. The U. S. Dairy Industry, A Vital Contributor To Economic Development. 2014.

24. González JJ, Gómez Benito C. Profession and identity. The case of family farming in Spain. Sociol Ruralis. 2001;41(3):343–57.

25. Mooney PH. My own boss? Class, Rationality, and the Family Farm. Boulder. 1988.

26. Hostiou N, Chauvat S, Cournut S. Faire face à des questions de travail : les leviers mobilisés par des éleveurs laitiers. In: INRA, editor. L'agriculture en famille : travailler, réinventer, transmettre [Internet]. Paris France; 2015. p. 125–43. Available from: http://open.edpsciences.net/index.php?option=com_content&view=article&id=290&Itemid=311#c008

27. Cournut S, Servière G, Hostiou N, Chauvat S, Dedieu B. L'organisation du travail en exploitations familiales d'élevage. Enseignements d'une analyse comparée conduite en France, en Amérique latine et au Vietnam. Cah Agric. 2010;19:338–47.

28. Garcia-Martinez A, Olaizola A, Bernués A. Trajectories of evolution and drivers of change in European mountain cattle farming systems. Animal. 2009;3:152–65.

29. Mc Elwee G, Robson A. Diversifying the farm: opportunities and barriers. Finnish J Rural Res Policy. 2005;4:84–96.

30. Dockes A-C, Couzy C, Daridan D, Gallot S, Magdelaine P, Pilorgé E, et al. Transformation de l'agriculture et des métiers des agriculteurs: quelles conséquences pour les organismes de

Développement ? In: Journées INRA-SFER de recherches en sciences sociales. 2007. p. 18.

31. Lémery B. Les agriculteurs dans la fabrique d'une nouvelle agriculture. Sociol Trav. 2003;45(1):9–25.

32. Constance DH, Choi JY, Lyke-Ho-Gland H. Conventionalization, bifurcation, and quality of life: certified and non-certified organic farmers in Texas. South Rural Sociol [Internet]. 2008;23(1):208–34. Available from: http://ag.auburn.edu/auxiliary/srsa/pages/Articles/SRS 2008 23 1 208-234.pdf

33. Stassart PM, Jamar D. Agriculture biologique et verrouillage des systèmes de connaissances Conventionalisation des filières agroalimentaire bio1. Innov Agron. 2009;2009(4):313–28.

34. Rosin C, Campbell H. Beyond bifurcation: Examining the conventions of organic agriculture in New Zealand. J Rural Stud [Internet]. 2009;25(1):35–47. Available from: http://dx.doi.org/10.1016/j.jrurstud.2008.05.002

35. Dalcq A-C, Beckers Y, Wyzen B, Reding E, Delhez P, Soyeurt H. Behavior patterns to the intensification vary differently within dairy producers. In: Proceedings of the 69th EAAP meeting. Dubrovnik; 2018.

36. Samson GS, Gardebroek C, Jongeneel RA. Explaining production expansion decisions of Dutch dairy farmers. NJAS - Wageningen J Life Sci [Internet]. 2016;76:87–98. Available from: http://dx.doi.org/10.1016/j.njas.2015.11.007

37. Mc Elwee G. The enterprising farmer: a review of entrepreneurship in agriculture. J R Agric Soc Engl [Internet]. 2006;January:1–9. Available from: https://www.academia.edu/2832784/The_enterprising_farmer_a_review_of_entrepreneurship_in_agriculture

38. Methorst R, Roep D, Verstegen J, Wiskerke JSC. Three-fold embedding: Farm development in relation to its socio-material context. Sustainability. 2017;9(10):1–19.

39. Keizer TH, Emvalomatis G. Differences in TFP growth among groups of dairy farms in the Netherlands. NJAS - Wageningen J Life Sci [Internet]. 2014;70:33–8. Available from: http://dx.doi.org/10.1016/j.njas.2014.03.001

40. Groeneveld A, Peerlings J, Bakker M, Heijman W. The effect of milk quota abolishment on farm intensity: Shifts and stability. NJAS - Wageningen J Life Sci [Internet]. 2016;77:25–37. Available from: http://dx.doi.org/10.1016/j.njas.2016.03.003

41. Willock J, Deary IJ, Edwards-Jones G, Gibson GJ, McGregor MJ, Sutherland A, et al. The Role of Attitudes and Objectives in Farmer Decision Making: Business and Environmentally-Oriented Behaviour in Scotland. J Agric Econ [Internet]. 1999;50(2):286–303. Available from: http://doi.wiley.com/10.1111/j.1477-9552.1999.tb00814.x

42. Zasada I, Piorr A. The role of local framework conditions for the adoption of rural development policy: An example of diversification, tourism development and village renewal in Brandenburg, Germany. Ecol Indic [Internet]. 2015;59:82–93. Available from: http://dx.doi.org/10.1016/j.ecolind.2015.02.002

43. Dufour A, Hostiou N, Cournut S, Dedieu B. Work organization on the dairy farm : visions of labour , working units and type of management varied. In: Rencontre Recherche Ruminants. Paris; p. 385–8.

44. Hansson H, Richard F, Olofsson C. Understanding the diversification and specialization of farm businesses. Agric Food Sci [Internet]. 2010;19:269–83. Available from: https://journal.fi/afs/article/view/5689

45. Müller B. My own boss? Strategies of resistance and accommodation of rural producers to neoliberal governance. Anthropologica [Internet]. 2010;52(2):233–6. Available from: http://ezproxy.library.uvic.ca/login?url=http://search.ebscohost.com/login.aspx?direct=true&db=psyh&AN=2010-25464-002&site=ehost-live&scope=site%5Cnbmuller@msh-paris.fr

Figure

Click here to access/download;Figure;Ideal future dairy farms to ensure revenue figure.eps

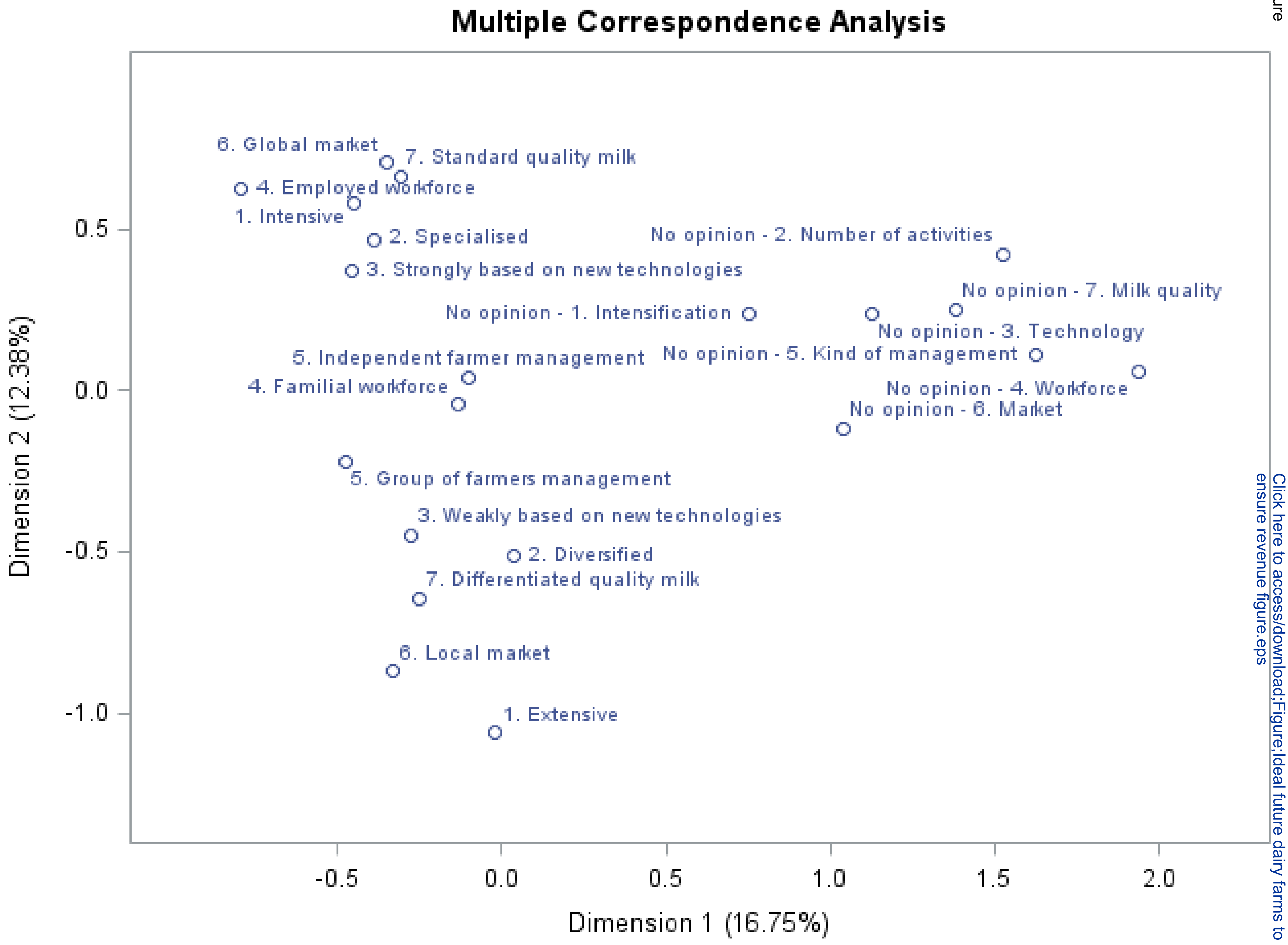

Figure 2

Click here to access/download;Figure;Ideal future dairy farms to ensure revenue AC Dalcq figure 2.eps

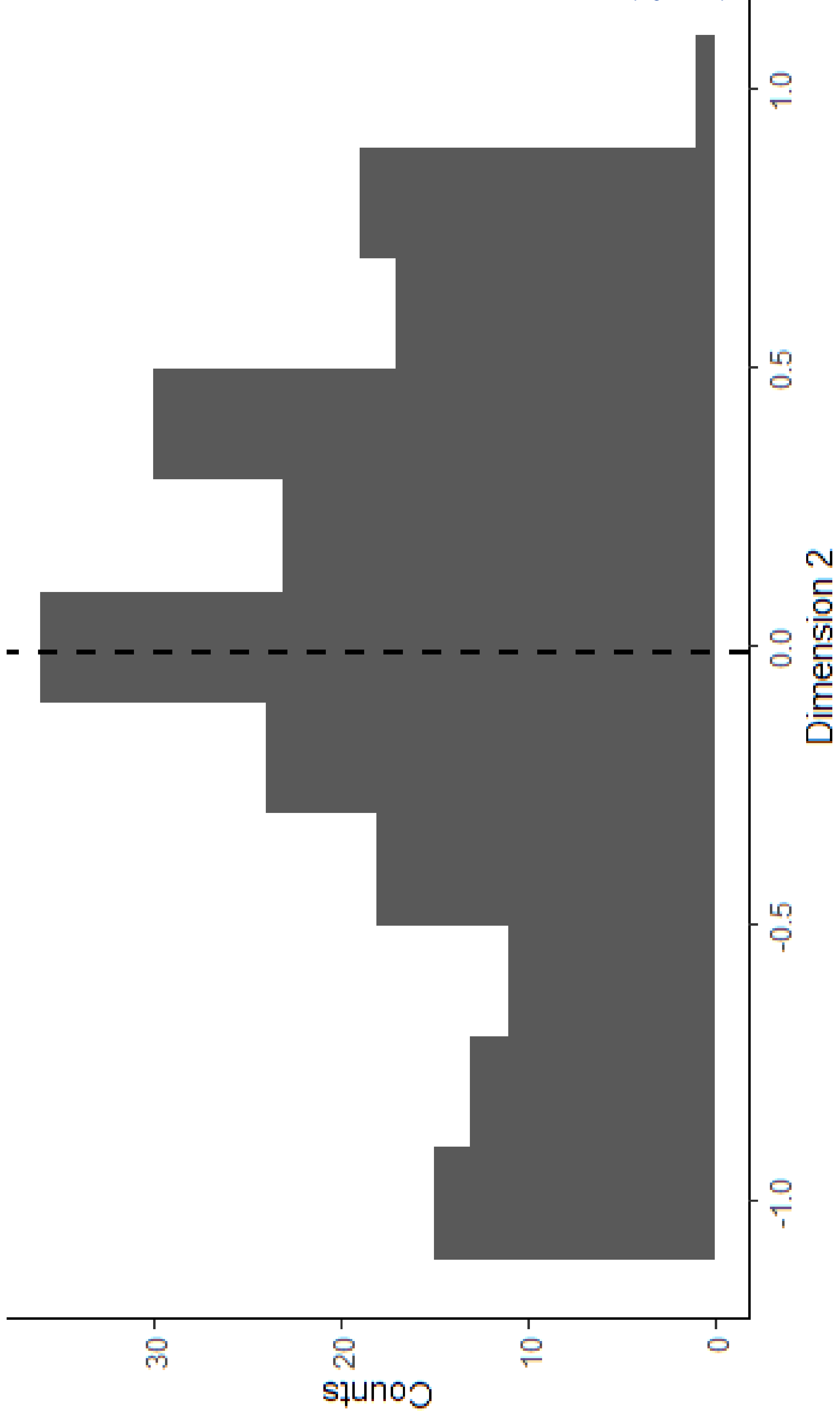

Other

Click here to access/download
Supporting Information
letter Ethics comittee.PNG

Click here to access/download
Supporting Information
S1 Appendix.docx

How do dairy farmers wish their future farm?

Anne-Catherine Dalcq[1]*, Thomas Dogot[1], Yves Beckers[1], Yves Brostaux[1], Eric Froidmont[2], Frédéric Vanwindekens[2], Hélène Soyeurt[1]

[1] University of Liège, Gembloux Agro-Bio Tech, TERRA Teaching and Research Centre, Passage des Déportés 2, 5030 Gembloux, Belgium

[2] Walloon Research Centre, Rue du Liroux, 8, 5030 Gembloux, Belgium

*Corresponding author

E-mail : anne-catherine.dalcq@uliege.be (ACD)

Ideal future dairy farms to ensure revenue

Abstract

Dairy farming systems are evolving. This study presents dairy producers' perceptions of their ideal future farm (**IFF**) to ensure revenue, and attempts to determine the reasons for this choice, the environmental aspects related to this choice, the proximity between the current farm and the IFF and the requirements for reaching this IFF. Just before the end of the European milk quota, a total of 245 Walloon dairy producers answered a survey about the characteristics of their IFF and other socio-environmental-economic information. A multiple correspondence analysis (**MCA**) was carried out using seven characteristics of the IFF (intensive *vs.* extensive, specialised *vs.* diversified, strongly *vs.* weakly based on new technologies, managed by a group of managers *vs.* an independent farmer, employed *vs.* familial workforce, local *vs.* global market, standard *vs.* quality-differentiated production) to observe the

relationships between them. Based on the main contributors to the second dimension of the MCA, this axis was defined as an IFF gradient between the local-based extensive (**LBE**) producers (26%) and the global-based intensive (**GBI**) producers (46%). The differences of IFF gradient between modalities of categorical variables were estimated using generalised linear models. Pearson correlations were calculated between the scores on the IFF gradient and quantitative variables. Finally, frequencies of IFF characteristics and the corresponding characteristic for the current situation were calculated to determine the percentages of "unhappy" producers. Some reasons for the choice of IFF by the producers have been highlighted in this study. Environmental initiatives were more valued by LBE than GBI producers. Low similarity was observed between the current farm situation of the respondents and their IFF choice. LBE and GBI producers differed significantly regarding domains of formation (technical and bureaucratic *vs.* transformation and diversification respectively) and paths of formation (non-market *vs.* market respectively). Two kinds of farming systems were considered by dairy producers and some socioeconomic and environmental components differed between them.

Introduction

Food is a basic need. Working to provide food for themselves and their family was the task of everyone at the dawn of humanity. The progressive organisation of society during the Neolithic period has led to the appearance of “producers” who are responsible for producing food for more than just themselves and their family (1,2). Since World War II, public policies have been set up to increase food production (3). These policies impacted the development of producers and their farms in the European Union. In the southern part of Belgium, the mean number of cows and the mean agricultural area per producer increased between 1980 and 2017 from 20 to 66 heads and from 25 to 71 hectares, respectively (4).

Producers are now facing great challenges to stay profitable. The price of the inputs (e.g. buildings, agricultural machinery, installations, feeding, veterinary care) of dairy production (**DP**) are increasing while the milk price shows great variability and its inflation is not similar to that observed for the inputs (5,6). Moreover, the European Union has decreased financial support to farmers (7). On 1st April 2015, the European Union removed the quota system which had managed the supply of DP (8). This led to greater milk price volatility. Additionally, sanitary crises such as mad cow disease (bovine spongiform encephalopathy (BSE)) and the dioxine crisis, among others, have shocked consumers and led to new rules and regulations at European level and to the creation of food security agencies in its countries. Moreover, these episodes modified consumers’ behaviours regarding their food purchases, they asked for more transparency and directed themselves towards organic food or local chains (9). Besides the economic view, the impacts of farming on the environment have been noted and policies have been set up in the Common Agricultural Policy to solve these problems (6,10).

In this context, the question often asked by dairy producers and stakeholders of the dairy sector is what the future of dairy farming entails, how to remain profitable and more generally sustainable. Several authors, such as Napoléone *et al.* (11), Havet *et al.* (5) and Lebacq (6), have studied the evolution of dairy farming and the present dairy systems, finding trends that exist in the sector. For instance, the project Mouve, funded by the French National Research Agency, studied the evolution of dairy farming systems in 6 dairy basins around the world. Their results gathered the publications of Napoléone *et al.* (11) and Havet *et al.* (5). Moreover, some other authors (e.g., Bergevoet *et al.* (12), Methorst *et al.* (13), Weltin *et al.* (14) and Verhees *et al.* (15) have studied the future paths of development considered by dairy producers. These studies were performed on the basis of data from 2001 to the beginning of 2013. They explored some reasons for these choices for the future.

This study is innovative as it asks what is the ideal future farm (**IFF**) perceived by the dairy farmers to ensure revenue. To our knowledge this question is not present in other studies. Moreover, respondent producers were asked not to take into account their current farm when considering their IFF. The data collection was conducted more recently, at the end of 2014 and the beginning of 2015. This was a particular context, just before the quota removal, when producers had this new perspective in mind. This change implied the disappearance of regulation of dairy supplies and was bringing uncertainty about the milk price. ~~caused volatility and decrease in the milk price~~ (16). We have assumed that this change in their working framework impacted respondents reflections and led them to reconsider their strategies, taking into account this new reality. They had just faced two important milk crises associated with low milk price in 2009 and an increase of the cost of inputs in 2012. This research studied unprecedented reasons for the choice of IFF compared to what is present in the

literature, to our knowledge, such as past events of the farms. Moreover, the present study explored the environmental and training aspects linked to this IFF vision. The environmental aspect is of high importance at a time of increasing awareness of the impacts of agriculture and breeding on the environment such as carbon footprints, biodiversity, etc. The topic of trainings for dairy producers was studied to orientate universities and other stakeholders of breeding improvement towards the domains needed and desired by dairy producers. A comparison between the current farm and the IFF of the respondent was realised, and permitted the difference between the reality and the aspiration of the producers to be studied. More specifically, the goals of this study were to answer the following questions: (1) What is the perception of dairy producers of their IFF? (2) How do dairy producers distribute themselves between IFF highlighted? ~~What is the proportion of producers desiring the different IFF?~~ By gathering different kinds of information, of which some are novel or rarely present in the literature, this study also answered to the following questions: (3) How do farmers decide on their IFF? (4) How do environmental aspects factor into IFF decisions? (5) Which paths and themes for training do farmers want in order to reach their desired IFF? And, ultimately, (6) how do farmers' IFF compare to their current dairy farming systems?

Materials and methods

Survey and IFF typology

In 2014, moving towards the end of the quota, as stakeholders of the dairy sector (research centre, agricultural sciences faculty, breeding association, agricultural unions, etc.), we wanted to know how the dairy producers of the southern part of Belgium will react to this change. We created a survey using LimeSurvey software (version 3.15.1+181017, LimeSurvey GmbH, Hamburg, Germany), which provides an internet link to get access and to complete the survey. The survey was first pre-tested orally with two dairy producers to estimate its duration and its clarity. The Board of Ethics and Scientific Integrity of the University of Liege waives the need for ethical approval. We communicated with Walloon dairy producers about the goals of the survey and its access broadly via all communication ways towards them : specialised press, agricultural internet websites, Unions and also advertisements through the milk payment letter which is sent to all the Walloon dairy producers once a month. The survey written in French can be viewed at the following internet link: https://www.gembloux.ulg.ac.be/enquete/index.php/219425?lang=fr and its English translation is viewable in the Appendix.

A total of 245 producers completed our survey between November 2014 and January 2015.

The entire survey was composed of 127 questions where the answers were decomposed into 498 categorical and 44 quantitative variables.

The question 'Without taking into account your current farm, what is, according to you, the ideal future farm to ensure a revenue?" was proposed to the producers and they

must ~~could~~ choose between short propositions on seven items: 1) intensive or extensive production; 2) specialised or. diversified activity (or activities); 3) farming strongly or weakly based on new technologies; 4) farm managed by an independent farmer or a group of managers; 5) family or employed workforce; 6) providing production for local or global markets; 7) providing standard or differentiated quality production. The modality “no opinion” was available for each IFF question. Counts were calculated for all modalities of these seven sub-questions.

The first step was to study if there were relationships between all modalities derived from the seven sub-questions asked. To achieve this objective, a multiple correspondence analysis (**MCA**) was carried out as the variables were categorical. For a MCA, the eigenvalue of the dimensions generated, named principal inertia, is a biased measure of the amount of information presented by a dimension (17). Corrected inertia rates were calculated, as described by Benzécri (18), to quantify the correct proportion of information of a dimension.

Classes were established to study the distribution of producers along the dimensions of the MCA. The interval between the 1% percentile and the 99% percentile of each dimension was divided equally into five classes. Then, the individuals per class were counted.

To exclude a group of producers with some ~~particular~~ characteristics if necessary, cluster analysis with the WARD method was used on the scores of the individuals on each dimension of the MCA. The WARD method is a hierarchical agglomerative method (19). The principle of this kind of method is to put initially the n individuals in n

groups and then to agglomerate the groups. The algorithm of WARD makes it in such a way that the gatherings induce the lowest decrease of R^2 at each step.

If a group of producers was excluded, its characteristics were previously studied against the remaining producers. The level of significance of the difference of the quantitative characteristics between the excluded and the remaining producers was studied thanks to general linear models. The level of significance of the difference of the proportions for each modalities of the qualitative characteristics between the excluded and the remaining producers was studied thanks to tests of proportions.

Characterisation of IFF choice

To describe the dairy producers in terms of their IFF, the scores on MCA dimensions were studied as a function of other variables extracted from the survey. This method was chosen instead of the creation of classes, possible with the Latent Class Analysis method or the Numerical Classification on the scores of MCA (Hierarchical Clustering on Principal Components). This choice was motivated by the wish to not put the producers in boxes but to study their position on a gradient between potential extreme models identified along the dimension.

The other variables extracted from the survey whose the relationships with the dimension are studied were distributed within several themes. These were the effect of past crises, problems encountered by the farmer, production factors, age of the farmer, breed of the cow, diversification of activities and alternative valorisation, regrouping between producers, consideration of mechanisation and robotisation on the farm, the reaction of the farmer to external factors, the considerations of farmers about environmental aspects, climatic hazards, ways to reach the ideal formation and field of formation. For categorical variables, the scores of MCA dimensions were modelled

using these variables as a fixed effect in a generalised linear model. Least squares means were estimated for the two-by-two comparisons using the Tukey test. The level of significance of those differences was assessed based on the *P*-value of the test. For quantitative variables, Pearson correlation coefficients were calculated between the scores of MCA dimensions and these variables. Their corresponding *P*-values were estimated to observe if the correlation values were significantly different from 0.

To observe if dairy producers presented the farming characteristics they considered to be ideal at the moment of survey, absolute frequencies (counts) were calculated as a function of each ideal future farm characteristic and of the answer to the question which corresponds to this ~~corresponding~~ characteristic for the current situation (Table 1). Moreover, the percentage of “unhappy” producers was calculated as the ratio between the producers not currently in the situation that they consider as ideal and the total number of producers.

All editing and statistical analyses were carried out using SAS software (version 9.4., SAS Inst. Inc., Cary, NC, USA).

Table 1. Absolute frequencies (counts) of producers as a function of their answer to the ideal future farm characteristic and the corresponding characteristic for the current situation and percentage of "unhappy" producers (*i.e.*, percentage of producers not currently in the situation that they consider to be ideal) (N = 245)

[1] Frequency in grey box corresponds to producers not currently in the situation that they consider as ideal regarding this characteristic

		**Corresponding characteristic for the current situation**		**% of "unhappy" producers**
		>2 cows per hectare of grass	<2 cows per hectare of grass	
**Ideal future farm characteristic**	Intensive	38	66[1]	50%
	Extensive	22	51	
		Only dairy production activity	Presence of activities other than dairy production	
	Specialised	46	59	37%
	Diversified	23	93	
		Presence of milking robot or agricultural equipment for a better technicality	Absence of milking robot or agricultural equipment for a better technicality	
	Strongly based on new technologies	33	52	37%
	Weakly based on new technologies	16	85	
		>1 chief operating officer or associates	1 chief operating officer	
	Managed by a group of managers	20	25	42%
	Managed by an independent farmer	68	108	
		Presence of workers (i.e., external person to family working on the farm)	No workers	
	With family workforce	17	195	10%
	With employed workforce	7	6	
	Providing dairy production for local *vs*. global market	No corresponding characteristic		
	Providing standard *vs*. differentiated quality dairy production	No corresponding characteristic		

Results and discussion

Data representativeness

The sample set of 245 producers represented 6.1% of the dairy producers in Wallonia (about 4,000 dairy producers in 2015 and 3,500 in 2017 (4)). The density of dairy farms throughout Wallonia was well represented in the sample, with a higher answer rate in the provinces more populated with dairy farms. More answers were obtained in the east part of Wallonia, where a higher density of dairy farms exists due to the grazing landscape that is particularly suitable for dairy production. Wallonia is a highly heterogeneous region with regard to soil and geological characteristics (20).

Dairy producers of the survey declared a mean of 79 cows and 86ha. Dairy production was their unique activity for 33% of them. The mean number of dairy cows per Walloon dairy farm was 52.9 cows in 2015 (21). No regional statistic exists on the mean agricultural area of all producers that perform a dairy activity. The mean agricultural area of specialised dairy farms and of all kind of farms taken together equated to 61.98 ha and 55.8 ha, respectively (21). So, the producers surveyed tended to have bigger farms regarding herd size and agricultural area than the average Walloon farm that have dairy activity.

What is the perception of dairy producers of their ideal future farm?

Univariate approach

As mentioned previously, the first aim of this study was to highlight the perceptions of Walloon dairy producers of their ideal farm, just before the end of the milk quota. This

was done through the answers to 7 sub-questions. Table 2 shows the frequency for each modality of those questions.

Table 2 Percentages of responses to the seven questions about the ideal future farm (N = 245)

**Question**<br>Without taking into account your current farm, what is, according to you, the ideal future farm to ensure a revenue?”	**Proposition**	**Percentage (%)**
Intensive *vs*. extensive	Intensive	43
	Extensive	30
	No opinion	27
Specialised *vs*. diversified	Specialised	43
	Diversified	47
	No opinion	10
Strongly *vs*. weakly based on new technologies	Strongly	35
	Weakly	41
	No opinion	24
Managed by an independent farmer *vs.* a group of managers	Independent farmer	72
	Group of managers	18
	No opinion	10
Family *vs*. employed workforce	Family	87
	Employed	5
	No opinion	8
Providing dairy production for local *vs*. global market	Global	43
	Local	32
	No opinion	25
Providing standard *vs*. differentiated quality dairy production	Standard	38
	Differentiated quality	45
	No opinion	17

Contrasting opinions of dairy farmers were observed for almost all questions except for the type of management and the kind of workforce: 71.84% of the respondents wanted an independent farmer management, and 86.53% focused on a family workforce (Table 2). These results highlight a will in the southern part of Belgium to maintain the traditional structure of work organisation in the future, with family workforce and one director of operations. More globally in the world, dairy farms are still mostly owned and managed by a family structure, whatever the degree of development of the country (22,23). The choice of producers to work by themselves and not to deal with workers (i.e., an external person to the family employed on the

farm) was noted in other studies. For example in Spain Gonzalez and Gomez (24) observed, when asking 3,370 farmers for their definition of a farmer, that more than half of them chose labourer and 12% chose businessman. In the USA in 1988, Mooney presented the fact that farmers had a particular status, being workers and employing other workers (25).

From Table 2, it is interesting to note that the highest percentages of abstention were observed for the questions about intensive *vs*. extensive, strongly *vs*. weakly based on new technologies, and providing DP for local *vs*. global markets. These results show that a quite significant proportion of the respondents did not take a position on these directions for the evolution of dairy farms.

Multivariate approach

To study the relationships between the answers given by the respondents to all questions about IFF, a MCA was performed as the related variables were categorical (Table 2). The percentage of principal inertia of the dimensions 1 and 2 of MCA were 16.75% and 12.38%, respectively (Fig 1). The value of corrected inertia for the two first dimensions reached 72.7% and 21.5% respectively, gathering almost 95% of the information.

Fig 1. Representation of the modalities in the multiple correspondence analysis first factorial plan. Values of principal inertia reached 16.75% and 12.38%. Values of corrected inertia reached 72.7% and 21.5%.

The first dimension of MCA showed positive relationships with the modalities no opinion of each characteristic and negative relationships with all the modalities with an opinion. Thus, the first dimension of the MCA allowed ~~permitted~~ differentiation between the producers who did not give their opinion concerning characteristics of IFF and the

producers who did (Fig 1). Cluster analysis was used to isolate the group of producers with a lot of ‘no opinion’ answers to the seven questions : this formed the first separation of classes of the analysis, dividing the “no-opinion” producers (15%) from the others (85%). The no opinion producers cluster (N = 38) was removed from the analysis to avoid potential bias coming from farmers who did not have a clear vision of their IFF.

Detailed information about this group is available in Tables 3 and 4. They tended to be older farmers (45-54 years), who came from Liège, which is a historic dairy region (Table 3). Even if it is not significant (*P* = 0.27), they are less likely to have other animal production on the farm (Table 3). Percentages of grass and corn silage observed for this group highlighted a same way ~~means~~ of feeding as the complete sample (Table 4). Even if these differences are not significantly different (*P* = 0.20, *P* = 0.59, *P* = 0.33), the more represented single breed and the lower number of cows but with the higher milk delivery quota of the no-opinion producers tended to express quite technical and high performing producers in this ~~the no-opinion~~ group. They were more ~~less~~, even if not significant, to plan ~~not projecting~~ to keep constant ~~increase~~ their production (Table 3). Even if not significant (*P* = 0.32), they declared more investment in the next five years than the complete sample, but ~~even if~~ no difference of investment was observed since 2009. They seemed to be people who have been dairy producers for a long time. We could assume that their farms had good economic performances and did not lead them to think about evolution in response to a great change (*i.e.* the quota removal). ~~Maybe they could also be ‘asleep’ in their old dairy traditions.~~

Table 3 Percentages of producers as a function of modalities of categorical variables for the no-opinion producers and the complete sample.

%	Complete sample N = 245	No-opinion producers N = 38	Producers with an opinion N = 207	*P* No-opinion *vs.* with opinion
**Age (years)**				
0-34	18	16	19	0.64
35-44	31	32	31	0.98
45-54	37	47	35	0.15
55-64	13	6	15	0.027
**Geographical situation**				
Walloon Brabant	6	2	6	0.24
Hainaut	32	34	31	0.74
Liège	34	45	32	0.14
Luxembourg	10	5	11	0.20
Namur	19	13	20	0.28
**Importance of dairy activity**				
Unique activity	33	34	32	0.83
Preponderant activity	65	61	65	0.59
Secondary activity	3	5	2	0.45
**Herd breed**				
Single breed	33	42	31	0.20
Multi-breed	67	58	69	0.20
**Other animal production**				
Yes	45	37	46	0.27
No	55	63	54	0.27
**Milk production evolution ~~for~~ in the next 5 years**				
Decrease	2	0	2	0.043
Constant	54	63	52	0.20
Increase	38	32	40	0.33
Stop	6	5	6	0.79
**Agricultural area investment since 2009**				
Yes	46	47	46	0.88
No	54	53	54	0.88
**Agricultural area investment in the next five years**				
Yes	57	64	55	0.27
No	43	36	45	0.27

Table 4: Means of quantitative variables for no-opinion producers and the complete sample

	Complete sample N = 245	No-opinion producers N = 38	Producers with an opinion N = 207	*P* No-opinion *vs.* with opinion
Agricultural area (ha)	86	87	85	0.80
Percentage of corn silage	15	14	15	0.67
Percentage of grass	61	60	60	0.76
Milk delivery quota (l)	558743	632880	545133	0.33
Number of cows	79	73	81	0.59

The second dimension of the MCA showed positive relationships with some modalities of the IFF characteristic and negative relationships with their opposite. Thus, this dimension seems ~~was the most interesting for highlighting~~ to highlight the wishes of dairy farmers about their IFF, for those who took a position on this question. More precisely, this axis showed a gradation of question modalities and proximity between several characteristics. The second dimension of the MCA was the most interesting for highlighting the wishes of dairy farmers about their IFF, for those who took a position on this question. This axis showed a gradation of question modalities and proximity between several characteristics. This dimension led to the identification of two extreme tendencies (Fig 1); the modalities of familial workforce, independent farmer management and management by a group of farmers were near to zero on this axis (Fig 1). This means that the small proportion of producers supporting group management was distributed between the two extreme tendencies observed. The position of the modalities of familial workforce and independent farmer at the middle of the second dimension illustrated the fact that these modalities were chosen by producers from the two tendencies identified. The small proportion of producers choosing an employed workforce was positioned at the top of the second dimension (Fig 1).

The first tendency, related to high scores on the second MCA dimension, corresponds to IFF with the following characteristics: global market, standard milk, intensive system, employed workforce, specialised and strongly based on new technologies. Other authors have observed the same relations. From a trial of 458 French dairy farms, Hostiou *et al.* (26) highlighted a profile of farmers which simultaneously gathered high equipment, intensification and workers. From a trial of 3,370 producers of all sectors in Spain, Gonzalez and Gomez Benito (24) collated the characteristics of large

holdings, market-orientated farming and management of workers. Cournut *et al.* (27) highlighted different ways of evolving dairy farming in France, characterised by workers, mechanisation and high equipment. This tendency in dairy farming systems is explained by the evolution of the dairy system (6). The increased competition in the dairy market caused by the creation of the open European market, as well as the wish of consumers to have structures that gather all the food supplies in one place (*i.e.* a supermarket) led to the concentration of dairy processing in a few big firms (11). These firms were better placed to develop because they could control their collection costs, benefit from scale economies and were able to deliver to supermarkets with regularity in quantity and with a standard quality (9). This state and the world market have conditioned milk prices for the producers. Increasing production, thanks to more cows or higher productivity, is a possible way to stay profitable, considering the undergone milk price (5,11). To achieve profitability, an elevated production of milk per cow and an increase of cows on the farm are reached (11). Moreover, this increase in milk production at farm level was also forced by the orientated production Common Agricultural Policy (**CAP**) primes, although CAP has limited help for the dairy sector. Therefore, all of these characteristics intensify the dairy farming system. Intensification was defined by Garcia-Martinez *et al.* (28) as the maximisation of the rarest factor, traditionally the agricultural area. The increase in DP per unit of agricultural area was possible thanks to intensive production of forage and purchase of inputs that are produced where production costs were the lowest, to balance the ration and to increase the production per cow, or the number of cows reared on a hectare of agricultural area and therefore DP per unit of agricultural area at the level of the farm (9,11). This intensification led to more specialised farms with more dairy cows and their entire workforce directed to this specialisation (9). The enlargement of farms required a

higher work rate; this was surmounted thanks to equipment and new technologies and to increased human workforce: collective organisation, subcontracting to private firms and employment of workers (9).

The second tendency, contrary to the first tendency, was characterised by high negative scores on the second MCA dimension. This axis was represented by the following modalities: weakly based on new technologies, diversified, differentiated quality milk, local market and extensive system (Fig 1). This reflects another form of dairy farming. This form is favoured by a constant increase in input prices, combined with a growing demand from consumers for high quality and local-based products (9). These dairy producers choose to work with greater self-sufficiency to be less dependent on the undergone input prices (9). The “localisation” of the production demanded by consumers was executed thanks to this more locally-produced forage and fewer inputs from outside (5). This return to self-sufficiency led to more extensive farming (5). The production induced was also often quality-differentiated and dedicated to local markets (9). Cournut *et al.* (9) showed in their study that this kind of dairy farming is chosen by a minority of farms, which are still diversified.

This gradation with two kinds of models at the extremities of the second MCA dimension was also described in other studies (5,6,9,11,29–31). They were named globalisation *vs*. territorialisation by Cournut *et al.* (9), or globalisation *vs*. localisation by Napoleone *et al.* (11). Lebacq (6) identified a “dualisation of dairy farming systems between ‘a mainstream model’ focusing on an increasing farm size, production intensity and specialisation and alternative models involving initiatives deviating from this trend and constituting niche developments (niches = minor elements, hardly sustainable against the mainstream model)”. Thanks to a survey answered by 180 producers of all sectors in 2007 in France, concerning the evolution of their farms and

their aspirations, Dockès *et al.* (30) also highlighted a major tendency towards the enlargement, professionalisation and specialisation of farms, but those authors also mentioned that other farms wanted to develop diversified structures, orientated towards the requests of society, processing and farm accommodation.

How do dairy producers distribute themselves between the ideal future farms highlighted? ~~What is the proportion of producers desiring a different ideal future farm?~~

The present study showed a bifurcation and quantified two ways: 46% *vs*. 26% of producers having high positive and high negative scores respectively on the second dimension (Fig 2). Verhees *et al.* (15) quantified producers as a function of their strategies of development, but solely regarding specialisation *vs*. diversification of their activity, 54.3% *vs* 15.1% respectively. The bifurcation phenomenon is also observed in the organic sector. Two models appeared: organic agriculture realised by historic actors and the other driven by the agribusiness to answer to a increasing organic demand (32–34).

Fig 2. Distribution of the producers along the second dimension (the dotted line represents the mean score on the second dimension of the producers)(N = 207)

How do farmers decide on their ideal future farm?

To study the relationships between the different IFF, the reasons for these and other interesting technico-economic information, the second dimension was considered as a gradient (**IFFg**) interpreted at the extremities as global-based intensive producers (GBI: high positive scores) and local-based extensive producers (LBE: high negative scores). The choice to work with a gradient rather than a clear separation of the two

tendencies was motivated by the will to not put dairy producers into boxes ~~pigeonholes.~~ The mean of the scores of the second MCA dimension was –0.012 with a SD of 0.053. Minimal and maximal values were –1.09 and 0.92, respectively.

Based on the interpretation of IFFg, a significant negative correlation indicates a higher relationship with the dairy producers desiring a LBE model. By opposition, a significant positive correlation means a higher link with the dairy producers desiring a GBI model. Tables 5, 7 and 8 give the results of generalised linear models where the categorical variables were introduced separately as a fixed effect in the model. Significantly lower estimates of IFFg for a specific modality of the considered categorical variable depicts a tendency of producers desiring a LBE model to choose this modality, while significantly higher estimates of IFFg means a tendency of producers wanting a GBI model to choose this modality. These analyses were conducted on the producers who have an opinion (N = 207). The following paragraphs will summarise the potential reasons driving the choice of IFF made by the Walloon dairy farmers.

Effect of past crisis on perceptions of the ideal future farm The producers that were impacted by past crises wished more for a LBE model (estimate = –0.17, Table 5). This could be related to the suffering involved in the crisis and the wish to apply solutions in order to not repeat this situation: revenue from diversified activities, other outlets for the milk production sold (*i.e.* local market characteristic) and/or self-sufficiency to be less dependent on purchased feed (*i.e.* extensive farm characteristic). This is in agreement with a past finding (35). We observed a decrease in intensification in 2012 which was the year of a dairy economic crisis mainly related to an increase in the price of inputs.

Table 5 Value and level of significance of the difference in the ideal future farm gradient as a function of modalities of categorical variables: Reasons (N = 207)

Means with different letters are significantly different.

Categorical variable	Modality and estimate					*P*
**Past crisis**						
Presence of deep modifications after crisis	No<br>0.031	Yes<br>–0.17				0.025
**Workload**						
Degree of arduousness	Not arduous<br>–0.11^{b}	Arduous<br>–0.092^{b}	Highly arduous<br>0.15^{a}			0.0043
Member of an agricultural replacement service	Yes<br>0.058	No<br>–0.0801				0.059
Worker engagement: help for workload and administrative aspects	Already implemented<br>0.024^{ab}	To implement in the future<br>0.13^{a}	Not interested<br>–0.040^{b}			0.25
**Production factors**						
Milk production evolution for 5 years	Decrease<br>–0.21	Constant<br>–0.13	Increase<br>0.052			0.036
Agricultural area investment since 2009	No<br>–0.14	Yes<br>0.14				0.0002
Investment (no agricultural area) since 2009	No<br>–0.19	Yes<br>0.015				0.055
Agricultural area investment in 5 years	No<br>–0.0909	Yes<br>0.046				0.073
Geographical situation	Brabant Walloon<br>0.11	Liège<br>0.12	Namur<br>0.055	Luxem-bourg<br>–0.013	Hainaut<br>–0.099	0.51
**Age**						
Age (years)	0-34<br>–0.086	35-44<br>0.0105	45-54<br>0.0049	55-64<br>0.0502		0.67
**Diversification and alternative valorisation**						
Presence of other animal production	Yes<br>–0.093	No<br>0.0603				0.037
Dairy processing and direct sales: sector developed if supported	Yes<br>–0.33	No<br>0.11				<0.001
Processing and direct sales (except dairy): sector developed if supported	Yes<br>–0.39	No<br>0.013				0.0096
HORECA, tourism and teaching activity to develop even if sustained	Yes<br>–0.18	No<br>0.018				0.055
Concern for diversification	Yes<br>–0.23	No<br>0.17				<0.001
Alternative chain for milk production valorisation	Yes<br>–0.49	No<br>0.036				<0.001
Alternative chain for “other than dairy” activity	Yes<br>–0.56	No<br>–0.0087				0.0017
Increase of “other than dairy” activity without investment	Yes<br>–0.42	No<br>–0.013				0.012
No activity to develop even if sustained	Yes<br>0.27	No<br>–0.10				<0.001
Increase of added value in farms: advantage of diversification and transformation	Yes<br>–0.097	No<br>0.11				0.0047
Link between producers and consumers: advantage of diversification and transformation	Yes<br>–0.14	No<br>0.066				0.0064
Conservation of farms in the region: advantage of diversification and transformation	Yes<br>–0.16	No<br>0.028				0.037

Financial, decisional and technical autonomy: advantage of diversification and transformation	Yes –0.27	No 0.030			0.005
Consumer loyalty: limit to diversification and transformation	Yes 0.17	No –0.047			0.028
Regulatory constraints (hygiene, *etc.*): limit to diversification and transformation	Yes –0.080	No 0.055			0.065
Size of investments: limit to transformation and diversification	Yes –0.14	No 0.0401			0.030
No constraints for transformation and diversification	Yes –0.093	No 0.042			0.052
No advantage of diversification and transformation	Yes 0.27	No –0.066			0.0006
**Breed**					
Composition of the herd	Single breed 0.18	Multi-breed –0.095			0.0005
	Pure-bred 0.0058	Dual purpose breed: –0.19			0.0023
**Regrouping**					
Advantage of fiscal and administrative aspects: advantage of grouping	Yes –0.16	No 0.023			0.050
Development of a joint project: advantage of grouping	Yes –0.15	No 0.020			0.072
Better marketing of the products: advantage of grouping	Yes –0.38	No 0.0063			0.030
**Mechanisation and robotisation**					
Mechanisation, robotisation: help with workload and administrative aspects	Into effect -0.031^{ab}	Not interested -0.094^{b}	To activate 0.12^{a}		0.041
**Reaction to external factors**					
Arduousness of the economic uncertainty of input price	No –0.19	Yes 0.0403			0.0089
Will of a dairy factory imposing production limits	Yes -0.23^{c}	No 0.25^{a}	Not important 0.0088^{b}		<0.001
Evolution of milk production during crises (2009, 2012)	Decrease -0.17^{b}	No variation -0.092^{b}	Increase 0.21^{a}		0.0006
Ideal size of the dairy factory	Small -0.52^{c}	Medium -0.11^{b}	Large 0.42^{a}	Not important -0.026^{b}	<0.001

Workload Workload seems to be less bearable for producers desiring a GBI model (estimate = 0.15, Table 5; R $_{\text{workforce constraint}}$ = 0.22, *P* = 0.002). Producers wishing for a GBI model were also more likely to be members of an agricultural replacement service (estimate = 0.058, Table 5) and showed a tendency to be more interested in employment of workers (estimate = 0.13, *P* $_{\text{worker engagement to implement } vs. \text{ not interested}}$= 0.11, Table 5). The choice of GBI model could be explained by this current workload, involving the need for an increase of revenue. So, the solution considered could be higher milk production and the breeding of more cows rather than diversification of

activities and self-valorisation activity, the development of which requires a lot of time. Samson *et al.* (36) confirmed this in the Netherlands by highlighting a nearly significant effect of labour productivity on the DP increase strategy.

Production factors The size of agricultural area, the milk delivery quota, the number of cows and the percentage of corn silage currently observed in the farming system showed significant and positive correlations with IFFg (R = 0.15, 0.36, 0.18 and 0.24; P = 0.033, <0.001, 0.0099, 0.0002 respectively). So, dairy producers choose their IFF partly as a function of their current production factors. This is expected as a higher number of hectares, cows and litres means a higher capacity of the dairy installation, of the material and so the possibility of a more preponderant dairy activity. The higher percentage of corn silage also reflected the possibility to seed corn silage, allowing the intensification of production as required within a GBI model. Similar relationships between characteristics of the farm and current or desired models of farming were observed by others. For Central and Eastern Europe, Verhees *et al.* (15) showed that land was the most important factor in developing a specific farming strategy. In France, Hostiou *et al.* (26) observed that intensified farms with higher technology equipment sometimes employed more workers, and were the farms with significantly higher agricultural area, percentage of corn silage, number of cows and milk quota. In the Netherlands, Samson *et al.* (36) showed that production intensity, number of cows, modernity of technology and availability of land were important factors in DP increase strategies.

In contrast, producers with lower production factors can consider rarely more enlargement and therefore think differently about the enhancement of their revenue: better valorisation of quality differentiated milk, other activities on the farm, self

valorisation, the LBE model. Samson *et al.* (36) showed that lower stable capacity varies inversely to a DP increase strategy, which is rather a GBI tendency.

The findings of the current study, as confirmed by previous researchers, showed that producers work within a tightly constrained and regulated environment limiting their ability to determine the future of their farm according to their personal desires. This statement was also concluded by Mc Elwee *et al.* (37) and Methorst *et al.* (38). In the Netherlands, Keizer and Emvalomatis (39) and Groeneveld *et al.* (40) showed that bigger farms are more likely to increase than other farms.

However, based on the quite low values of the correlations obtained between the agricultural area and the number of cows, we can consider that this situation must be nuanced and that the IFF chosen also depends on the opinions of the dairy producer, not taking into account the current situation of his farm. This statement is reinforced by the fact that the correlation of percentage of meadow with IFFg was not significantly different to 0 ($R = -0.097$, $P > 0.1$). Also, the impact of the provinces of the Walloon Region, which present different geographical and soil characteristics, on IFFg were not significantly different ($P = 0.51$, Table 5).

Moreover the significant relations between IFFg and milk production evolution for five years (Table 5; $R_{\text{quantity of milk variation}} = 0.30$, $P < 0.001$), investment for and in five years (Table 5) support the assumption that the IFF chosen depends greatly on the mentality of the producers.

In their study, Methorst *et al.* (13) proved the heterogeneity of farm developments of producers facing the same socio-material context, showing the importance of the mentality of the producers in their decisions. Authors speak about shared values, norms, ways they see themselves or would like to be seen by producers, views,

capacities and their perceptions of opportunities and any room for manoeuvre, skills, motives, entrepreneurship, goals and strategies (12,13,36,38,41) as factors which influence farm development. Samson *et al.* (36) discussed experimental economics, which are economics where psychology and biology, which explain human behaviours, are added to better explain the development of enterprises. The consideration of more than just economic aspects permits them to reduce the error of their model for predicting DP increase strategies (36).

Age Age of the producer seems not to condition the desired IFF (Table 5). An IFF could be chosen because of either the new ideas of young producers or the experience of older producers. If mentality seems to influence IFF choice, it is not linked to age. The two kinds of IFF could be an answer to both innovation and problems encountered during a long career. Samson *et al.* (36) also studied age as a reflection of the farmers' values, goals and strategies, and showed no relationship with DP increase, which is rather a GBI characteristic. On the contrary, on the basis of data from 11 countries of the European Union, Weltin *et al.* (42) observed an effect of age on the tendency towards diversification, which is rather a LBE tendency.

Diversification and alternative valorisation The results obtained in this study showed a link between the diversification mentality and the choice of LBE model. Significant negative estimates or correlations were observed for the following variables related to diversification: the presence of other animal production (estimate = –0.093, Table 5); the direct selling milk quota (R = –0.17, P = 0.016); dairy or no dairy processing and direct sales (estimates = –0.33 and –0.39, Table 5); the development of HORECA activities, tourism and teaching (estimate = –0.18, Table 5); the concern for diversification (estimate = –0.23, Table 5); alternative chain for milk and other than milk production valorisation (estimates = –0.49 and –0.56, Table 5) and the increase

of “other than dairy” activity without investment (estimate = –0.42, Table 5). Conversely, producers desiring a GBI model were more likely to choose the item “no activity to develop if supported”, suggesting the unique principal activity way of thinking of producers aiming for a GBI model (estimate = 0.27, Table 5). Samson *et al.* (36) confirmed this tendency and showed that the presence of diversified activities evolved inversely to the increase of milk production. In this study, we observed potential explanations to support to this fact. Producers wishing for a LBE model considered self-valorisation and diversification as solutions to the current situation to enhance revenue due to the creation of added value (estimate = –0.097, Table 5). They thought that diversification and transformation allowed financial, decisional and technical autonomy (estimate = –0.27, Table 5) and were confident in consumer loyalty (estimate = –0.047, Table 5). They considered relations with consumers as an opportunity and not a threat, unlike producers desiring a GBI model (estimate = 0.17, Table 5). One reason GBI model producers gave against self-valorisation and diversification seemed to be the lack of trust in consumers and therefore the outlets. They frequently saw no advantage to self-valorisation and diversification (estimate = 0.27, Table 5). The relation to the consumer was also studied by Verhees *et al.* (15). They observed that consumer orientation was more often declared as an opportunity by the profiles of producers considering strategies similar to LBE. The positive impact of diversified activities on autonomy was also shown by Bergevoet *et al.* (12). They mentioned that proponents of the “extra source of income” model (closest to the LBE model) were more able to declare that they can increase the sales-price of their milk. Producers wishing for a LBE model were also likely to find no constraints to transformation and diversification (estimate = –0.093, Table 5). The only limits to diversification and transformation highlighted by producers wanting a LBE model were regulatory

constraints (estimate = –0.080, Table 5) and the size of investments (estimate = –0.14, Table 5). As a consequence of these considerations, producers wanting a LBE model felt that they were more able to meet society's expectations regarding local and artisanal products (R = –0.22, *P* = 0.0016) and the desire for a familial structure (R = –0.12; *P* = 0.084).

Breed to produce milk Producers wanting a LBE model are more open to breeding a dual-purpose herd (estimate = –0.19, Table 5), which permits them to diversify their production: milk and meat. Producers wishing for a GBI model target a single, more specialised breed (estimate = 0.18, Table 5) which could offer more homogeneous management of the herd. The link between mentality, observed through the choice of breed(s), and the choice of IFF is once more highlighted.

Regrouping Producers tending towards the LBE model were more likely to promote regrouping for its advantages regarding fiscal and administrative aspects, the development of a joint project and the marketing of the products (estimates = –0.16; –0.15; –0.38, Table 5). The importance of mentality for the choice of IFF has been shown. A mentality of cooperation, as a solution to enhance their quality of life and revenue, tends to be shared between producers desiring a LBE model.

Mechanisation and robotisation We observed that the wish of technology of producers tending towards GBI model can be explained by the fact that they considered it as help for workload. ~~A "pro-technology" mentality of the producers tending towards the GBI model was observed~~ (estimate = 0.12, Table 5). It can be assumed that the solution considered by them is to keep the same activity or increase it with help from machines. In southern France, Dufour *et al.* (43) observed the propensity of farmers with workers, close to the GBI model, to prioritise investment in equipment. Verhees *et al.* (15) observed that better management, including new

technologies, was more cited as an objective for producers whose strategy profiles were more similar to the GBI than LBE models.

Reaction to external factors Reactions of dairy producers to factors external to their decision-making power tend to be different as a function of their choice of IFF, showing once more a different mentality of the producers. Producers wanting a LBE model tend to show themselves to be more independent from the external economic actors: from the input producing companies (estimate = –0.19, Table 5) and from the market and factories, rejecting contracts which would link them to it (R = –0.13, Table 6). When their opinion about dairy factories was surveyed, producers desiring a LBE model preferred small or medium units with production limits (estimates = –0.52; –0.11; –0.23, Table 5), as before, which means regulation of the dairy offerings on the market. Producers wishing for a GBI model direct themselves to big units of processing without production limits (estimates = 0.42; 0.25, Table 5) and so more turned towards world markets. They recognise the freedom in regarding DP as an asset of quota removal (R = 0.23, Table 6). The reaction regarding the quantity of production was not similar during a crisis, producers wanting a LBE model tended to maintain or decrease their production (estimates = –0.17; –0.092, Table 5), whereas producers desiring a GBI model tended to increase production (estimate = 0.21, Table 5). The latter wanted to keep revenues constant with more litres produced when the price decreased, while the others controlled or decreased production when the gross margin per litre decreased. This can be due to a deliberate choice to decrease milk production or a decision to decrease the variable costs causing a decrease in milk production. These results can express a fear of producers tending toward the LBE model in considering world markets, contrary to producers tending towards the GBI model who have decided to work with this kind of market. Verhees *et al.* (15) observed that producers projecting

strategies similar to the LBE model consider the market more as a threat than producers projecting strategies similar to the GBI model. Hansson *et al.* (44) and Weltin *et al.* (14) explained that this uncertainty and risk perception can explain the choice of diversification, which is a part of the strategy of the LBE model.

Couzy and Dockès (7) demonstrated different profiles of farmers and observed the entrepreneurship mentality of each one, which highlights similar tendencies to those presented here. Several profiles showed strong entrepreneurship but which was expressed differently to here. A category of farmers showed entrepreneurship by their wish for autonomy of decision in their management; they will keep a working approach close to the conventional one but with a modernist vision, always adapting to the market. They want to keep freedom in the classical framework. In 1988, Mooney described the split personality of producers: they are independent people, making their own decisions regarding their way of working and their investments but at the same time are people dependent on different processing actors and banks (45). Another category of farmers showed entrepreneurship by their wish to develop an original idea, away from preexisting systems, a project in line with their conviction to be freer from the existing system [5].

Samson *et al.* (36) and Methorst *et al.* (13) reported that decisions of producers cannot be reduced to only economic aspects: this includes policies and market conditions but also their way of thinking about them.

Table 6 Correlations (R) between the ideal future farm gradient and quantitative variables (N = 207)

*producers declaring no calling of replacement services were removed from this analysis

How do environmental aspects factor into IFF decisions?

The environmental aspects related to the desired IFF were studied as awareness of the environmental impact of breeding has become an important issue of our time.

Producers tending toward the GBI model seemed to work with a higher livestock manure application pressure (R = 0.16, Table 6) and therefore are already more likely to work in an intensified dairy system, which can have a greater impact on the environment. Samson *et al.* (36) showed a tendency toward manure production surplus by producers with increasing DP, which is rather a GBI characteristic.

Results of practices that are in accordance with the environment: measurement of the grass height, forage mixture with leguminous plants, use of a field notebook (estimates = –0.27; –0.11; –0.074, Table 7) showed a stronger interest from producers wanting a LBE model.

Quantitative variable	R	*P*
**Reaction to external factors**		
Contract means dairy production more integrated to dairy factories: level of agreement	–0.13	0.076
Quota removal means more flexibility concerning production: level of agreement	0.23	0.0014
**Considerations of the environmental aspects**		
Degree of the constraint: livestock manure application	0.16	0.022
Facility to answer to society’s expectations: environmentally friendly agricultural practices	–0.15	0.027
Agricultural activity is important for rurality of villages: level of agreement	–0.23	0.0011
Agricultural activity is important for conservation of permanent grasslands: level of agreement	–0.27	<0.001
Agricultural activity is important for biodiversity: level of agreement	–0.18	0.0101
Agricultural activity is important for planting and maintenance of hedges: level of agreement	–0.28	<0.001
Importance of answering society’s expectations for the revenue of the dairy producers: level of agreement	–0.11	0.11
Ease of answering society’s expectations: landscape and territory maintenance: level of agreement	–0.19	0.0065
**Needs: formation method**		
*Frequency of calling replacement services for meeting and formations (N = 104)	0.21	0.066

Table 7 Value and level of significance for the difference in the ideal future farm gradient as a function of modalities of categorical variables: Environmental aspects (N = 207)

Means with different letters are significantly different.

Categorical variable	Modality and estimate		*P*
**Considerations of environmental aspects**			
Measurement of the grass height: optimisation practice	Yes<br>–0.27	No<br>0.0083	0.059
Forage mixture with leguminous plants: optimisation practice	Yes<br>–0.11	No<br>0.083	0.0088
Field notebook: optimisation practice	Yes<br>–0.074	No<br>0.061	0.065
**Climatic hazard**			
Increase of concentrate distribution: strategy to confront climatic hazards	Yes<br>0.22	No<br>–0.036	0.036
Decrease of the herd: strategy to confront climatic hazards	Yes<br>–0.25	No<br>0.014	0.037
Food self-sufficiency: cause for maintaining constant or decreased milk production	Yes<br>–0.17	No<br>0.0073	0.14

Besides these, all the significant negative correlations between IFFg and the levels of agreement with an agricultural area are important for the rurality of villages (R = –0.23, Table 6), for conservation of permanent grasslands (R = –0.27, Table 6), for biodiversity (R = –0.18, Table 6) and for hedges (R = –0.28, Table 6) showed the importance of the environment in the dairy activity of producers wanting a LBE model. It can be assumed that both LBE producers and GBI producers have concerns for the environment but in different ways. These results showed that LBE producers are more willing to employ the benefits of ecosystem services, which is observable in this database. Moreover, they found it easy to realise environmentally friendly agricultural practices, as asked for by society (R = –0.15, Table 6) and which are important to answer to society's expectations to guarantee their revenue (R = –0.11, Table 6).

Bergevoet *et al.* (12) had a considerably more consistent opinion. The "extra-source of income" profile producers (showing similarities with the LBE model) were more likely to declare that in their decision-making they take the environment into consideration, even if it lowers profit. The "large and modern farm" profile producers do not mention their will to adopt these initiatives.

Climatic hazard Facing feed shortages due to unfavourable climatic conditions, producers tending toward GBI and LBE seem not to have the same way of thinking; GBI producers intend to buy high nutritional feed to balance shortages (estimate = 0.22, Table 7) and LBE producers are going to decrease the number of cows (estimate = –0.25, Table 7) and ensure their feed autonomy (estimate = –0.17, Table 7).

How do farmers' ideal future farm compare to their current farming systems?

The current situation of dairy producers was compared to their preferred IFF (Table 1). Except for the type of workforce, quite high percentages of “unhappy” producers were observed for the farm characteristics, between 37 to 50%. This suggested that not all producers work as they would like to. The same comparison was not found in the literature, to our knowledge.

As dairy producers do not work in a way that they consider to be ideal, it is interesting to study the gaps to fill in order to reach their ideal system and so, amongst others, their needs. The study of the requirements to reach the IFF, including ways to meet these needs and the area of the needs, can inform the stakeholders of the dairy sector about what must be developed to evolve into IFF.

Which paths and themes of training do dairy producers want in order to reach their desired ideal future farm?

Paths to formation As way to improve their skills, producers wanting GBI tended to favour consultancy (estimate = 0.17, Table 8) and commercial companies (estimate = 0.16, Table 8) and not days of study on other farms (estimate = 0.082, Table 8), meanwhile producers wanting LBE supported this latter possibility (estimate = –0.088,

Table 8), a network of pilot farms (estimate = –0.13, Table 8) and the associate, non-market sector (estimate = –0.21, Table 8). Moreover, for help in technical choices, producers desiring LBE chose formation and study days (estimate = –0.15, Table 8) and producers' technical groups to implement in the future (estimate = –0.20, Table 8). The choices presented confirm the will for a non-market way to learn for producers wanting LBE, contrary to producers wishing for GBI.

As an information source, the agricultural press was commonly cited (N = 161, *i.e.* 78% of respondents), but producers desiring LBE tend to not want to inform themselves in this conventional way (estimate =–0.14, Table 8).

Producers wanting a GBI model tend to need more help to free them from their work in order to follow a formation (R = 0.21, Table 6)

Table 8 Value and level of significance of the difference in the ideal future farm gradient as a function of modalities of categorical variables: formations (N = 207)

Means with different letters are significantly different.

Categorical variable	Modality and estimate			P
**Needs: ways to learn formations**				
**Consultancy company: SFI* place	Yes<br>0.17		No:<br>–0.087	0.0017
**Study days on farm: SFI place	Yes<br>–0.088		No<br>0.082	0.026
**Network of pilot farms: SFI place	Yes<br>–0.13		No<br>0.056	0.025
**Associate, non-market sector SFI place development	Yes<br>–0.21		No<br>0.062	0.0023
**Commercial company: SFI place	Yes<br>0.16		No<br>–0.058	0.014
***Agricultural press: information source	Yes<br>0.025		No<br>–0.14	0.068
Formation and study day: help for technical choices	Already implemented<br>0.037[a]	To implement in the future<br>–0.15[b]	Not interested<br>0.035[a]	0.082
Producers technical groups: help for technical choices	Already implemented<br>0.020[a]	To implement in the future<br>–0.20[b]	Not interested<br>0.11[a]	0.0046
**Needs: domain formation**				
**Finance and management: requested formation	Yes<br>0.066		No<br>–0.24	0.0007

**Processing and diversification: requested formation	Yes<br>–0.18		No<br>0.089	0.0008
**Plant selection: requested formation	Yes<br>0.083		No<br>–0.053	0.087
**Animal selection: requested formation	Yes<br>0.080		No<br>–0.082	0.034
**Animal feeding: requested formation	Yes<br>0.03		No<br>–0.14	0.073
**Administrative: requested formation	Yes<br>0.064		No<br>–0.11	0.026
**Legal framework: requested formation	Yes<br>0.14		No<br>–0.083	0.005
Request for advice: help for financial aspects	Already implemented<br>0.014[a]	To implement in the future<br>–0.15[b]	Not interested<br>0.049[a]	0.19

*SFI = study, formation and information

**producers declaring no will of formation were removed from this analysis

*** producers declaring no agricultural press as an information source were removed from this analysis

Formation domains The formation domains reflected the direction chosen by producers looking for LBE and the ways to reach it. They tend to want skills related to processing and diversification (estimate = –0.18, Table 8) and were likely to reject finance, management (estimate = –0.24, Table 8), administrative (estimate = –0.11, Table 8) and legal framework skills (estimate = –0.083, Table 8). For financial aspects producers wanting LBE tend to favour requests for advice from experts rather than self-formation (estimate = –0.15, $P_{\text{to implement } vs. \text{ not interested}}$ = 0.12, Table 8). They do not choose animal feeding (estimate = –0.14, Table 8) and selection formations (estimates = –0.053; –0.082, Table 8). This could suggest the will of the producers not to change their way of management and the level of quality of their herd but the method of valorisation of their production.

In contrast, producers desiring GBI tend to want to continue to enhance their vegetal and animal production (estimates = 0.083; 0.08, Table 8), to become more efficient and enhance their revenue. Moreover they are more interested in legal aspects (estimate = 0.14, Table 8). Expansion and complexification of the GBI model of dairy

farms wished for by these producers could be an explanation. Bergevoet *et al.* (12) also observed a will to be well informed about the legislation for the “modern and large farm” profile. This is not noted in their profile, which is close to the LBE model.

Two kinds of formation were identified and preferred by producers wanting LBE or GBI models. Bergevoet *et al.* (12) observed the will to innovate for the two profiles closest to LBE and GBI profiles of this study. Verhees *et al.* (15) observed that formation was the most important resource for dairy producers. The present research differentiated the formation desired as a function of IFF. Dufour *et al.* (43) defined, through a survey of 15 dairy farmers, three conceptions of the work: difficult, organisational and passionate. The passionate approach was accompanied by the desire for new knowledge which was, as observed here, either to learn about genetic selection or about processing and marketing of products.

Conclusions

In conclusion, the GBI tendency is two times more represented than the LBE tendency. Many reasons explain this choice of ideal farm. Past crises seem to cause farmers to desire the LBE model. A high workload seems to orientate respondents to the GBI model. The wish for the IFF is influenced by the current framework but is also a question of mentality. Production factors reached, breeds chosen for the herd, ways to react to factors external to the farm, consideration of diversification and alternative valorisation, regrouping and mechanisation and robotisation describe the producers' mentality and showed different relations with the IFF chosen. Moreover LBE and GBI producers may both have concern for the environment, but the approach to act for the environment by LBE producers, through concern for ecosystem services, is clearly highlighted in this study. These producers found it important to answer to society's expectations. Finally, as the current situation of farming is quite different to the ideal one, the learning needs were studied and two types of customer appeared in relation to their formation. We conclude that two kinds of dairy producers seem to appear, for different reasons, with different relations to the environment and asking for different formations.

Acknowledgments

I want to thank the organising committee of "Carrefour des Productions animales" for the supply of the data.

References

1. Mazoyer M, Roudart L. Histoire des agricultures du monde. Du néolithique à la crise contemporaine. Editions du Seuil; 1997. 1–705 p.

2. Lefèvre D. Des racines et des gènes. Une histoire mondiale de l'agriculture. Volume 1. Rue de l'Echiquier; 2018. 1–567 p.

3. European Economic Community. Treaty of Rome. 1957.

4. STATBEL. Exploitations agricoles et horticoles - plus de chiffres [Internet]. 2019. Available from: https://statbel.fgov.be/fr/themes/agriculture-peche/exploitations-agricoles-et-horticoles/plus

5. Havet A, Cournut S, Correa P, Madelrieux S, Poccard R, Corniaux C, et al. Tandem changes in livestock farming and feed systems since 1960: a study of six dairy-producing regions. Fourrages [Internet]. 2015;222:115–24. Available from: http://apps.webofknowledge.com/full_record.do?product=UA&search_mode=GeneralSearch&qid=3&SID=R2ZtQywoePOzfzM5uQW&page=16&doc=153&cacheurlFromRightClick=no

6. Lebacq T. La durabilité des exploitations laitières en Wallonie - Analyse de la diversité et voies de transition [Internet]. UCLouvain, Louvain-la-Neuve, Belgium; 2015. Available from: http://www.philagri.net/wp-content/uploads/2015/03/TL_redaction_these_20150302.pdf

7. Couzy C, Dockes A-C. Are farmers businesspeople? Highlighting transformations in the profession of farmers in France. Int J Entrep Small Bus [Internet]. 2008;6(3):407–20. Available from: http://www.inderscience.com/search/index.php?action=record&rec_id=19135&prevQuery=&ps=10&m=or

8. SPW Environnement Agriculture Ressources naturelles, Département de l'Etude du Milieu naturel, Direction de l'Analyse Économique et agricole. Agriculture Evolution de l'économie agricole et horticole de la Wallonie 2013-2014. 2013.

9. Cournut S, Rapey H, Nozières MO, Poccard-Chapuis, R., Corniaux C, Choisis JP, et al. Dynamics of livestock farming in extensive livestock farming territories : what processes are going on ? In: 10th European IFSA Symposium. Aarhus, Denmark; p. Unpaginated Ref. 26.

10. FAO. Livestock’s Long Shadow. Environ issues options. 2006;1.

11. Napoléone M, Boutonnet JP, Corniaux C, Alavoine-Mornas F, Barritaux V, Carvalho S, et al. Between local and global : which reconfiguration within Dairy Basins ? A comparative analysis from North & South case-studies. In: 10th European Social Science History Conference. Vienna, Austria; 2014. p. 1–12.

12. Bergevoet RHM, Ondersteijn CJM, Saatkamp HW, Van Woerkum CMJ, Huirne RBM. Entrepreneurial behaviour of dutch dairy farmers under a milk quota system: Goals, objectives and attitudes. Agric Syst. 2004;80(1):1–21.

13. Methorst RG (Ron., Roep D (Dirk), Verhees FJHM (Frans., Verstegen JAAM (Jos. Differences in farmers’ perception of opportunities for farm development. NJAS - Wageningen J Life Sci [Internet]. 2017;81:9–18. Available from: http://dx.doi.org/10.1016/j.njas.2017.02.001

14. Weltin M, Zasada I, Franke C, Piorr A, Raggi M, Viaggi D. Analysing behavioural differences of farm households: An example of income diversification strategies based on European farm survey data. Land use policy [Internet]. 2017;62:172–84. Available from: http://dx.doi.org/10.1016/j.landusepol.2016.11.041

15. Verhees F, Malak-Rawlikowska A, Stalgiene A, Kuipers A, Klopčič M. Dairy farmers’ business strategies in Central and Eastern Europe based on evidence from Lithuania, Poland and Slovenia. Ital J Anim Sci [Internet]. 2018;17(3):755–66. Available from: https://doi.org/10.1080/1828051X.2017.1422154

16. Salou T, van der Werf HMG, Levert F, Forslund A, Hercule J, Le Mouël C. Could EU dairy quota removal favour some dairy production systems over others? The case of French dairy production systems. Agric Syst [Internet]. 2017;153:1–10. Available from: http://dx.doi.org/10.1016/j.agsy.2017.01.004

17. Palm R. L’analyse à correspondances multiples : principes et application. Notes Stat d’informatique. 2007;

18. Benzécri JP. Sur le calcul des taux d’inertie dans l’analyse d’un questionnaire. Cah l’analyse des données. 1979;4:377–8.

19. Everitt BS, Landau S, Leese M, Stahl D. Cluster Analysis, 5th Edition. Shewhart WA, Wilks SS, editors. Wiley; 2011. 1–330 p.

20. Service Public de Wallonie. La Carte des Sols de Wallonie [Internet]. Available from: http://geoportail.wallonie.be/catalogue/ce3b6602-1c52-483f-9133-770009cdd02b.html

21. SPW Environnement Agriculture Ressources naturelles, Agricole, Département de l'Etude du Milieu naturel, Direction de l'Analyse Économique et agricole. Evolution de l'économie agricole et horticole de la Wallonie 2017. 2017.

22. De Haan C. Smallholder dairy production. In: Agriculture Investment Sourcebook. 2011. p. 169–73.

23. USDA/ERS. The U. S. Dairy Industry, A Vital Contributor To Economic Development. 2014.

24. González JJ, Gómez Benito C. Profession and identity. The case of family farming in Spain. Sociol Ruralis. 2001;41(3):343–57.

25. Mooney PH. My own boss? Class, Rationality, and the Family Farm. Boulder. 1988.

26. Hostiou N, Chauvat S, Cournut S. Faire face à des questions de travail : les leviers mobilisés par des éleveurs laitiers. In: INRA, editor. L'agriculture en famille : travailler, réinventer, transmettre [Internet]. Paris France; 2015. p. 125–43. Available from: http://open.edpsciences.net/index.php?option=com_content&view=article&id=290&Itemid=311#c008

27. Cournut S, Servière G, Hostiou N, Chauvat S, Dedieu B. L'organisation du travail en exploitations familiales d'élevage. Enseignements d'une analyse comparée conduite en France, en Amérique latine et au Vietnam. Cah Agric. 2010;19:338–47.

28. Garcia-Martinez A, Olaizola A, Bernués A. Trajectories of evolution and drivers of change in European mountain cattle farming systems. Animal. 2009;3:152–65.

29. Mc Elwee G, Robson A. Diversifying the farm: opportunities and barriers. Finnish J Rural Res Policy. 2005;4:84–96.

30. Dockes A-C, Couzy C, Daridan D, Gallot S, Magdelaine P, Pilorgé E, et al. Transformation de l'agriculture et des métiers des agriculteurs: quelles conséquences pour les organismes de

Développement ? In: Journées INRA-SFER de recherches en sciences sociales. 2007. p. 18.

31. Lémery B. Les agriculteurs dans la fabrique d'une nouvelle agriculture. Sociol Trav. 2003;45(1):9–25.

32. Constance DH, Choi JY, Lyke-Ho-Gland H. Conventionalization, bifurcation, and quality of life: certified and non-certified organic farmers in Texas. South Rural Sociol [Internet]. 2008;23(1):208–34. Available from: http://ag.auburn.edu/auxiliary/srsa/pages/Articles/SRS 2008 23 1 208-234.pdf

33. Stassart PM, Jamar D. Agriculture biologique et verrouillage des systèmes de connaissances Conventionalisation des filières agroalimentaire bio1. Innov Agron. 2009;2009(4):313–28.

34. Rosin C, Campbell H. Beyond bifurcation: Examining the conventions of organic agriculture in New Zealand. J Rural Stud [Internet]. 2009;25(1):35–47. Available from: http://dx.doi.org/10.1016/j.jrurstud.2008.05.002

35. Dalcq A-C, Beckers Y, Wyzen B, Reding E, Delhez P, Soyeurt H. Behavior patterns to the intensification vary differently within dairy producers. In: Proceedings of the 69th EAAP meeting. Dubrovnik; 2018.

36. Samson GS, Gardebroek C, Jongeneel RA. Explaining production expansion decisions of Dutch dairy farmers. NJAS - Wageningen J Life Sci [Internet]. 2016;76:87–98. Available from: http://dx.doi.org/10.1016/j.njas.2015.11.007

37. Mc Elwee G. The enterprising farmer: a review of entrepreneurship in agriculture. J R Agric Soc Engl [Internet]. 2006;January:1–9. Available from: https://www.academia.edu/2832784/The_enterprising_farmer_a_review_of_entrepreneurship_in_agriculture

38. Methorst R, Roep D, Verstegen J, Wiskerke JSC. Three-fold embedding: Farm development in relation to its socio-material context. Sustainability. 2017;9(10):1–19.

39. Keizer TH, Emvalomatis G. Differences in TFP growth among groups of dairy farms in the Netherlands. NJAS - Wageningen J Life Sci [Internet]. 2014;70:33–8. Available from: http://dx.doi.org/10.1016/j.njas.2014.03.001

40. Groeneveld A, Peerlings J, Bakker M, Heijman W. The effect of milk quota abolishment on farm intensity: Shifts and stability. NJAS - Wageningen J Life Sci [Internet]. 2016;77:25–37. Available from: http://dx.doi.org/10.1016/j.njas.2016.03.003

41. Willock J, Deary IJ, Edwards-Jones G, Gibson GJ, McGregor MJ, Sutherland A, et al. The Role of Attitudes and Objectives in Farmer Decision Making: Business and Environmentally-Oriented Behaviour in Scotland. J Agric Econ [Internet]. 1999;50(2):286–303. Available from: http://doi.wiley.com/10.1111/j.1477-9552.1999.tb00814.x

42. Zasada I, Piorr A. The role of local framework conditions for the adoption of rural development policy: An example of diversification, tourism development and village renewal in Brandenburg, Germany. Ecol Indic [Internet]. 2015;59:82–93. Available from: http://dx.doi.org/10.1016/j.ecolind.2015.02.002

43. Dufour A, Hostiou N, Cournut S, Dedieu B. Work organization on the dairy farm : visions of labour , working units and type of management varied. In: Rencontre Recherche Ruminants. Paris; p. 385–8.

44. Hansson H, Richard F, Olofsson C. Understanding the diversification and specialization of farm businesses. Agric Food Sci [Internet]. 2010;19:269–83. Available from: https://journal.fi/afs/article/view/5689

45. Müller B. My own boss? Strategies of resistance and accommodation of rural producers to neoliberal governance. Anthropologica [Internet]. 2010;52(2):233–6. Available from: http://ezproxy.library.uvic.ca/login?url=http://search.ebscohost.com/login.aspx?direct=true&db=psyh&AN=2010-25464-002&site=ehost-live&scope=site%5Cnbmuller@msh-paris.fr

Dear Academic Editor, dear Reviewers,

First, I would like to thank you for the time spent to improve the understanding of this manuscript. This letter is organized in two parts. The first part deals with the main issue of the review (i.e., the method mobilized, which corresponds to the point raised by the academic editor and one remark of the reviewer). The second part concerns the specific answers to the specific remarks formulated by the reviewers.

Sincerely,

Anne-Catherine Dalcq

1/ Methodology

Please read carefully the propositions of reviewer #2 especially about the recommendations you did not took into account in your revision. I am afraid the reviewer is right about the interest of LCA vs MCA, as we expect a characterization of different profiles of dairy farmers.

But my proposition is that instead of LCA, you use HCPC (Hierarchical Clustering on Principal Components) which a natural and standard extension of MCA (see Arguelles et al. 2014, Kassambara 2017 or the following technical report http://factominer.free.fr/more/HCPC_husson_josse.pdf).

H. Soyeurt :

Dear Editor,

Dear Reviewers,

Instead of my PhD student, Miss Anne-Catherine Dalcq, I would like to answer to the question related to the methodology used in this study. I am Professor Hélène Soyeurt and I teach courses related to Data Mining and Machine Learning at Gembloux Agro-Bio Tech (University of Liège). Therefore, I have an experience in the use of multivariate analysis. It is why I would like to answer by myself to the question related to the method used in this article.

Before starting an explanation, I would like to precise that we have not really understood the comments of the reviewer about Latent Class Analysis (LCA) because the objective was not to create groups of farmers. Indeed, and this is the innovative aspect of this paper, a gradient between two quite different models of farms was studied. The gradient is really important because it appears to us simplistic to classify farmers in only 2 groups, working with a gradient allowed us to nuance the position of the dairy producers and to analyze more precisely the link between this position and other characteristics. The use of the gradient allows studying the trend of a farmer. It is why in this paper, we always mention "tend towards" to make a reference to the position on the gradient and not a binary choice of a model.

As we did not want to create farmer's clusters, LCA was not appropriate as well as HCPC. However, as asked by the second reviewer to prove the robustness of our approach, we have decided to show you the similarities and the extended work that it is possible to do using Multiple Correspondence Analysis (MCA) and LCA. Indeed, it is also possible to use LCA to create a gradient instead of using the clusters.

So, I would like to remind the methodology that we have proposed in this study (see the figure below, Fig 1)). Again, MCA was used to observe the relationships between the seven studied variables. Based

on the interpretation of MCA dimensions, we have observed that the second dimension represented the positioning between the two models for a dairy farm. Therefore, the score for this dimension for a specific producer allows to know its perception of ideal future farm between the two extreme models. This gradient allows avoiding to limit the farm typology to 2 clusters. This is interesting because many farmers combined some approaches specific to one model of farms (no more intensification but extensification, local-based market) or another one (global-based market, continuously improvement of the productivity thanks to, notably, intensification, …). Therefore, wishing to split the dairy farmers into 2 groups is too limitative. Some farmers remain between these two models (Fig 2), some farmers are more convinced than others by a model. Farmers showing higher scores are really convinced by GBI model, farmers showing lower but positive scores choose GBI model but are not 100% in this way of farm development. Negatives scores express the position of farms in favor of LBE model, the lowest scores reflected pronounced adhesion to this model. It is why the gradient as proposed in this paper was useful and seem to us the most interesting tool to represent dairy producers.

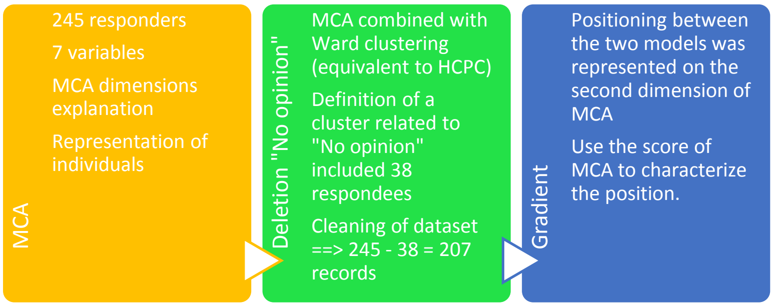

Figure 1 Methodology

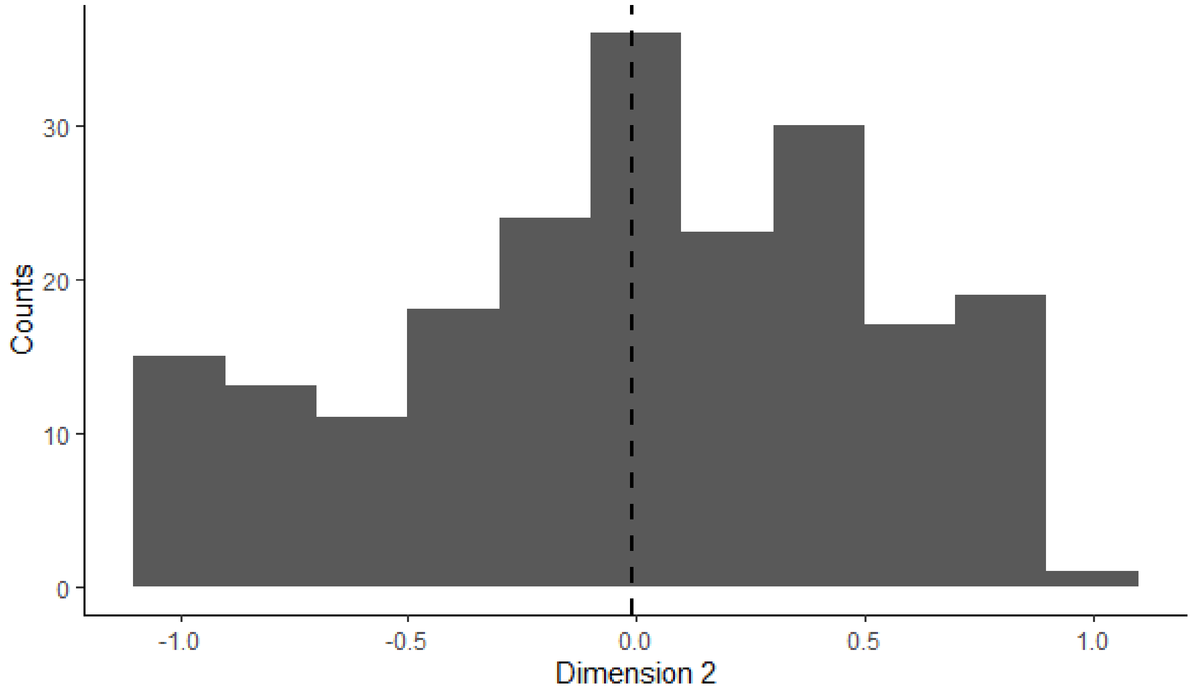

Figure 2 Distribution of the producers (the "with an opinion" ones) along the second dimension of the MCA

Similarly, we have done this job using LCA methodology as requested by the reviewer#2. LCA allows defining clusters from the dataset. In this case and to be in line with the study objective, we have decided to create 3 clusters. Moreover, based on the AIC and BIC values, it was also the best model (Fig 3). LCA allows us to create those 3 clusters. After the interpretation of defined clusters, it appeared that we have a cluster representing “No opinion” responders, one cluster representing “global-based intensive” (**GBI**) farmers and one cluster representing “local-based extensive” (**LBE**) farmers (Fig 4). Therefore, those clusters were similar to the ones obtained by combining MCA and Ward clustering (=HCPC method) as done to clean the dataset (i.e., extract responders with “no opinion” behavior). Again, we did not want to use those clusters to make our analysis. But, using this methodology, it is possible to obtain a probability to belong to a specific cluster. Therefore, we have decided to compare the gradient as defined in the current study to the probability to belong to the LBE or GBI clusters defined using the LCA method.

So, now, it is time to present you the results. I will not present you the results about MCA as those results are reported in the article. In this paragraph, we will focus on the LCA results. As we used categorical variables which are not ordinal, we have decided to use the polytomous latent class analysis. The variables used, called manifest variables, were the same than the one used for MCA. The modalities for each variable were recoded from 1 to 3:

- Intensification: 1. Intensive, 2. Extensive, 3. No opinion
- Number of activities: 1. Specialised, 2. Diversified, 3. No opinion
- Technology: 1. Strongly based on new technologies, 2. Weakly based on new technologies, 3. No opinion
- Workforce: 1. Familial workforce, 2. Employed workforce, 3. No opinion
- Kind of management: 1. Group of farmers management, 2. Independent farmer management, 3. No opinion
- Market: 1. Global market, 2. Local market, 3. No opinion
- Milk quality: 1. Standard quality milk, 2. Differentiated quality milk, 3. No opinion

As this clustering is very sensitive to the prior values used to start the iteration, 10 repetitions were used to provide you the final results for all models. All calculations were done with R software and more specifically the package poLCA. First, we have run different LCA models using a different number of classes (clusters). We have tested models from 1 to 10 classes and then we have estimated the AIC and BIC criteria to observe which model allowed the best fitting. The results of AIC and BIC for all models are presented in the figure below (Fig 3). From this figure, we can conclude that the model allowing the creation of 3 classes is a good comprise between BIC and AIC (i.e., the lowest BIC and AIC values).

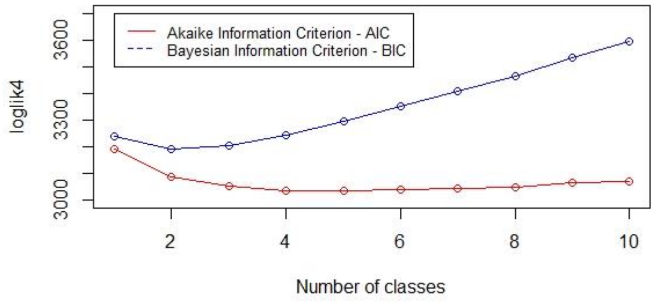

Figure 3 AIC & BIC criteria values

The figure below shows you the clusters defining by the model allowing a discrimination of the data into 3 clusters (Fig 4). This figure represents the probability to have a specific modality for each cluster. So, based on those results, we can conclude that the first clustering (class 1) is related to GBI dairy producers, the second cluster (class 2) is related to LBE dairy producers and the last cluster (class 3) is related to the dairy producers with “no opinion”.

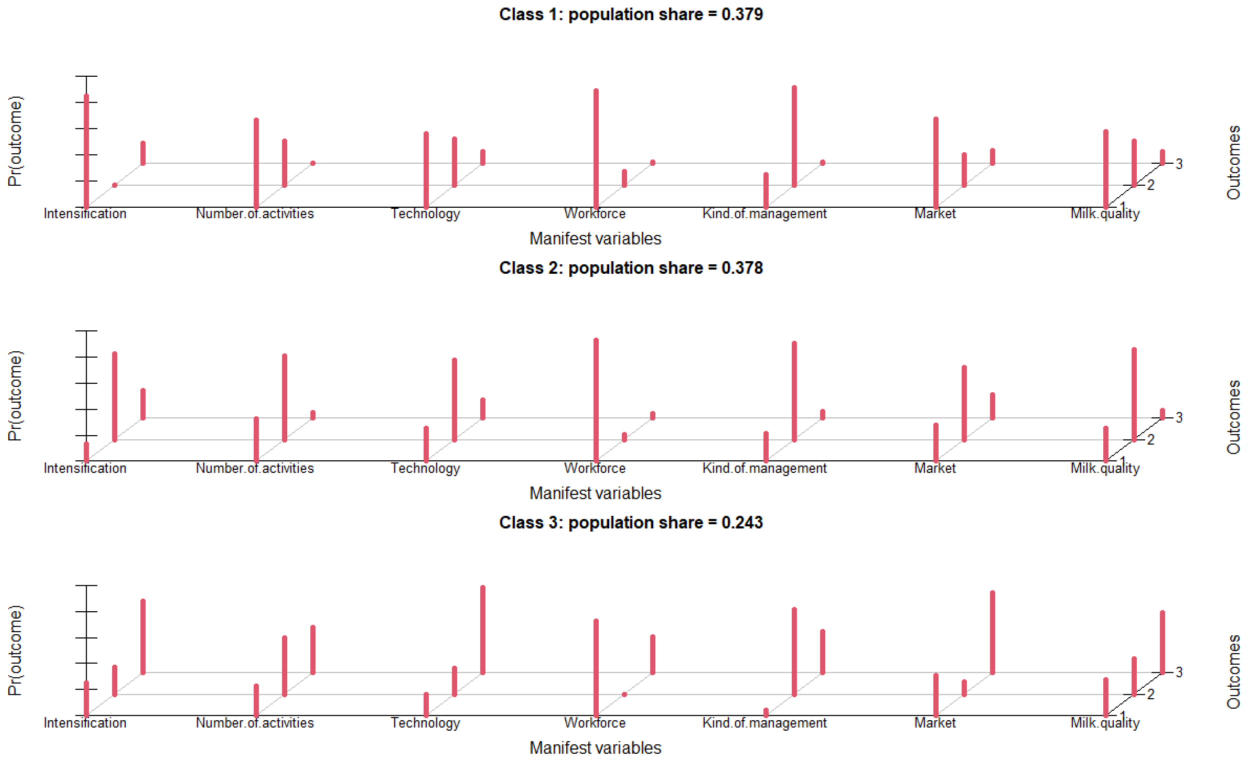

Figure 4Description of the three clusters obtained by LCA method

Therefore, in order to clean the dataset (i.e., delete records from farmers having many “no opinion” views), we can use the clustering 3. This process is similar to the MCA + Ward clustering (HCPC) proposed in the manuscript. Again, we did not want to use clusters but we want to use a gradient. For MCA, this gradient was the second MCA dimension. In the context of LCA, this gradient can be derived from the probability to belong to class 1 (“GBI producer”) or class 2 (“LBE producer”). So, to show the robustness of the MCA approach used, we calculated the correlation between those probabilities and the score of the second MCA dimension (called gradient in the manuscript). The correlation between the probability to belong to class 1 and the gradient was equal to 0.83. The correlation between the probability to belong to class 2 and the intensification gradient was equal to -0.87. The correlation between the 2 probabilities was equal to -0.97.

From all of those results, you can see that the relationship is strong between MCA and LCA using an innovative approach focusing on the score/probability of an individual and not directly to a cluster. Using only the second dimension of MCA, we can reflect both clusters (class 1 and class 2) simultaneously. Indeed, the second dimension is a gradient “GBI-LBE” and included both. This is really interesting to observe the relationships between this gradient and other quantitative or qualitative variables as now the studied trait is quantitative. It allows to see if a modality of a categorical variable is the choice of really convinced GBI dairy producers or dairy producers only sticking out of the GBI model.

I hope that this demonstration illustrates well the relevancy of the approach proposed in our paper. A sentence will be added in the materials and methods section to explain why LCA was not used. Moreover, some articles exist also in the literature to prove the mathematical relationships between MCA and LCA (e.g., Lautsch and Plichta, Psychology Science 2003:298-323 as well as Van der Heijden, et al., Sociological methodology 1999).

The fact that the two dimensions explained almost 95% of the variability and that all the modalities representing an opinion positioning themselves along the second dimension support us to take the second dimension as gradient, after deletion of the No-opinion producers which were discriminated by the first dimension.

Finally, I would like to acknowledge you for the great job done in the review of this paper. The manuscript was improved a lot.

Sincerely,

Prof Hélène Soyeurt

PS : The answers to all other comments were done by Anne-Catherine Dalcq.

2/ Others comments

Reviewer #2: In this revision, the authors have significantly improved the English language translation and clarified their research questions, but they have failed to address the shared concerns of reviewers regarding methods and clarity of writing. They have not adequately addressed the substance of the comments from reviewers.

Regarding methods, the reviewers still do not offer a compelling and clear justification for why MCA is appropriate for their goals and they do not offer the latent class analysis that I suggested or the hierarchical clustering (related) suggested by reviewer 3 as alternatives or robustness checks. MCA, while performed adequately, is not well suited to the way they discuss their results. They continually refer to "types" of respondents, which is what latent class analysis is for. MCA is about identifying clustering of variables, not clusters of respondents. All of their interpretation of results is about clustering and patterns of PEOPLE, not variables. This indicates a significant misalignment between the method and the research goals.

A-C Dalcq: First, we would like to thank you for the deep reading done on this article and for your formulated comments. We missed your request to test the robustness of our method with other ones. We apologize for this mistake. You will find our work of comparison with LCA method in the first part of this letter. In the article, we speak about producers tending towards "GBI" or "LBE" models to express the results coming from the use of our gradient. Indeed, MCA is a method to identify relationships between variables but is also suitable to make groups of individuals thanks to the use of its extension HCPC, as proposed by the academic editor. A detailed information about the comparison of MCA and LCA is now given in the first part of this letter.

The authors do not adequately discuss the two dimensions of the MCA (figure 1) and the axes are not adequately labeled. It is not made clear why the second dimension is retained.

A-C Dalcq: We added some information. The updated explanations of the two dimensions are taken up just after (Lines 254-261/286-306/339-343). Do you need more information? If it is the case, could you guide us?

Remark : Lines specified throughout this letter are those of the revised manuscript with track changes.

The axes are labeled as the figure is provided by SAS 9.4. We precised its meaning thanks to the caption and the following interpretation.

Explanations of the two dimensions:

Lines 254-261: "The first dimension of MCA showed positive relationships with the modalities no opinion of each characteristic and negative relationships with all the modalities representing an opinion. Thus, the first dimension of the MCA allowed ~~permitted~~ differentiation between the producers who did not give their opinion concerning characteristics of IFF and the producers who did (Fig 1). Cluster analysis was used to isolate the group of producers with a lot of 'no opinion' answers to the seven questions: this formed the first separation of classes of the analysis, dividing the "no-opinion" producers (15%) from the others (85%)."

Lines 286-306: "The second dimension of the MCA showed positive relationships with some modalities of the IFF characteristic and negative relationships with their opposite. Thus, this dimension seems ~~was the most interesting for highlighting~~ to highlight the wishes of dairy farmers about their IFF, for those who took a position on this question. More precisely, this axis showed a gradation of question modalities and proximity between several characteristics. This dimension led to the identification of two extreme tendencies (Fig 1); the modalities of familial workforce, independent farmer management and management by a group of farmers were near to zero on this axis (Fig 1). This means that the small proportion of producers supporting group management was distributed between the two extreme tendencies observed. The position of the modalities of familial workforce and independent farmer at the middle of the second dimension illustrated the fact that these modalities were chosen by producers from the two tendencies identified. The small proportion of producers choosing an employed workforce was positioned at the top of the second dimension (Fig 1)."

"The first tendency, related to high scores on the second MCA dimension, corresponds to IFF with the following characteristics: global market, standard milk, intensive system, employed workforce, specialised and strongly based on new technologies."

Lines 339-343: "The second tendency, contrary to the first tendency, was characterised by negative scores on the second MCA dimension. This axis was represented by the following modalities: weakly based on new technologies, diversified, differentiated quality milk, local market and extensive system (Fig 1). This reflects another form of dairy farming. »

Reason of the use of the second dimension:

*Lines 379-384: "To study the relationships between the different IFF, the reasons for these and other interesting technico-economic information, the second dimension was considered as a gradient (**IFFg**) interpreted at the extremities as global-based intensive producers (GBI: high positive scores) and local-based extensive producers (LBE: high negative scores). The choice to work with a gradient rather than a clear separation of the two tendencies was motivated by the will to not put dairy producers into boxes ~~pigeonholes~~"*

While the authors have provided a link to the survey online, that link is only in French and requires registration with an email address before it can be viewed, so that is not adequate. The authors still have not addressed the real concern that I raised in my previous review: they need to be clear about how they measured their concepts (intensive/extensive, etc.), what specific survey questions were used, and how those variables were coded. A clear list of questions for each concept and the coding

for each is needed. For instance, in Table 2, it is not clear what specific survey questions or variables represent these concepts and how those variables were actually coded.

A-C Dalcq: In the current version, we have added an annex (Appendix 1) with the translation of the questions mobilized in the paper. Given the length of the survey, we provided only the questions raised in the present paper. Some information are mentioned at lines 129-136 about the questions related to the ideal future farm characteristics (intensive vs. extensive,…), the way of measurement for our developed concepts. If you need more information, could you precise them to us explicitly ?

Lines 129-136: "The entire survey was composed of 127 questions where the answers were decomposed into 498 categorical and 44 quantitative variables. The question 'Without taking into account your current farm, what is, according to you, the ideal future farm to ensure a revenue?" was proposed to the producers and they must ~~could~~ choose between short propositions on seven items: 1) intensive or extensive production; 2) specialised or. diversified activity (or activities); 3) farming strongly or weakly based on new technologies; 4) farm managed by an independent farmer or a group of managers; 5) family or employed workforce; 6) providing production for local or global markets; 7)providing standard or differentiated quality production. The modality "no opinion" was available for each IFF question."

The authors still do not address response rate for the survey. They do now address representativeness of their respondents for this specific region, but they have not addressed the bigger questions of representativeness: how does this one region in one nation represent that nation, Europe, and/or agriculture broadly?

A-C Dalcq: The response rate of 6,1% was already precised in the past manuscript. You can find it at lines 200-201 of the current text.

Lines 200-201 : "The sample set of 245 producers represented 6.1% of the dairy producers in Wallonia (about 4,000 dairy producers in 2015 and 3,500 in 2017 (STATBEL, 2019)*)."*

For the second part of your comment, do you want that we precise that the Walloon Region is one of the two regions of Belgium, which is one of the 27 members countries of the European Union? If yes, the proposal could be : "The Walloon Region is one of the two regions of Belgium, which is one of the 27 members countries of the European Union".

Or do you want that we precise the number of dairy producers in Belgium and in the European Union? The International Dairy Federation mentions 9,674 dairy farms in Belgium and 1,130,700 farms with a dairy activity in the European Union (Confédération Belge de l'Industrie Laitière, 2020)*. The number of Walloon dairy farms is obviously low amongst all of these countries. We do not know if all this information is relevant to be written in the manuscript.*

Complete reference: Confédération Belge de l'Industrie Laitière. 2020. Rapport Annuel 2020.

The goal of this paper is to inform about the position of dairy producers of a region, which is moreover quite heterogeneous regarding the geopedologic conditions (lines 205-206), this one can represent the context and the resources of other producers in Europe. The goal was not to give a complete vision of all the European producers, which needs higher means.

In all tables the n, or respondent totals, should be clear.

A-C Dalcq: We added a sentence at lines 395-396 :"These analysis were conducted on the producers who have an opinion (N = 207)." And the N was precised and added in each table. Thanks for this remark which brings clarity throughout all the paper.

For Table 5 there are subscripts/footnotes that are never defined or labeled.

A-C Dalcq: The subscripts are now precised in each table. "Means with different letters are significantly different.". Thanks for this remark.

For Tables 3 and 4, no significance tests are reported.

A-C Dalcq: Indeed, the goal was not to test the differences between the no-opinion producers and all the sample but to give an idea of the characteristics of the No-opinion producers. Reviewer#3 asked us to give all of this information also for producers with an opinion. We realized ANOVA tests between the no-opinion producers and the producers with an opinion (lines 280-284)

The interpretation of the MCA results is circular logic. They define the clusters based on variables such as the attitude toward technology and then present a finding that people who are in the "pro-technology" GBI cluster have more positive attitudes towards technology. Of course, that is how you defined the scale in the first place.

A-C Dalcq: The variable "technology" is "Mechanisation and robotisation : help for workload and administrative aspects " and is present in the part "Reasons". This result is presented to explain one reason of the producers tending towards "GBI-model" to tend to this model and one of its component, the technology. We have better precised our idea by adding the sentence: "We observed that the wish of technology of producers tending towards GBI model can be explained by the fact that they considered it as help for workload." at lines 534-536.

Regarding writing, the presentations of results and its mixing with discussion of existing literature is still extremely unclear and difficult to follow. Both reviewer 1 and myself raised this critique: presenting your results intermingled with other literature is difficult to read and makes it unclear what your key findings are. This is not about the technical requirements of the journal. In its current presentation, readers cannot easily identify what your key findings are in each subsection and it is very difficult to read. For instance, in the section on pages 15-16, the authors spend substantially more time discussing other studies than they do their own results.

A-C Dalcq: As already mentioned and visible at lines 304-364 in the revised paper, the explanations based on the findings of other past studies help to explain the relationships observed between the modalities of the seven ideal future farm characteristics.

The introduction is improved, but still weak. The first paragraph is overly general and does nothing to build the focus of the paper. The authors also spend too much time asserting the contribution of their study before they have even reviewed the literature or told us what their analysis will be.
A-C Dalcq: What could be your expectations about the structure of this introduction? As the reviewer#3 did not make comments about the redaction of this part and without deeper expectations from your, the structure of the introduction was not changed.

The attempts to incorporate new literature are cursory.

A-C Dalcq: The work of Mr Mooney was consulted and two references was added to the paper (lines 236-238, lines 577-580). Did you expect references to more elements of his work? Could you precise which ones? Moreover, we also investigated the phenomenon of bifurcation. Literature about bifurcation was mainly found for the organic activity. We consulted a Professor with skills in sociology, Prof Kevin Maréchal, of Gembloux Agro-Bio Tech-University of Liège (Belgium), who provided us also this literature reference. Do you have other literature to advice to me?

The paper is not appropriately written for a general audience. They assume too much prior knowledge from readers regarding methods both methods and the case.

A-C Dalcq: We have now added information about the choice of the method between MCA and LCA, and about the method WARD (regarding a following remark). We hope that it brings the missing information. If this information is not complete for you, could you precise us explicitly the requested information?

Lines 165-170: "This method was chosen instead of the creation of classes, possible with the Latent Class Analysis method or the Numerical Classification on the scores of MCA (Hierarchical Clustering on Principal Components). This choice was motivated by the wish to not put the producers in boxes but study their position on a gradient between potential extreme models identified along the dimension."
Lines 153-156 : "The WARD method is a hierarchical agglomerative method (Everitt et al., 2011). *The principle of this kind of method is to put initially the n individuals in n groups and then to agglomerate the groups. The algorithm of WARD makes it in such a way that the gatherings induce the lowest decrease of* R^2 *at each step."*

Overall, the authors have inadequately addressed the careful feedback of the reviewers and made inadequate improvements. Throughout the response to reviewers they reject several important critiques with no justification of their rejection.

A-C Dalcq: We recognized that we do not explain the choice of the method and we do not test its robustness with statistic treatments. We apologized for that. We missed this request. We realized this analysis at this time.

A number of specific points are highlighted below:

Line 79- How was that ensured (respondent producers were asked not to take into account their current farm when considering their IFF)?

A-C Dalcq: It is now precised in the Materials and methods section (Lines 131-134). Thanks for your remark.

Lines 131-134 : The question 'Without taking into account your current farm, what is, according to you, the ideal future farm to ensure a revenue?" was proposed to the producers and they must choose between short propositions on seven items: 1) intensive or extensive production; 2)[…]"

Line 121- survey link not accessible without registering. Include in appendix?

A-C Dalcq: The appendix is realized and available in the new submission.

Line 146- What is WARD?

A-C Dalcq: We added sentences of explanation about the Ward method at lines 153-156. It is a hierarchical agglomerative method of Numerical classification.

Lines 153-156: "The WARD method is a hierarchical agglomerative method (19). The principle of this kind of method is to put initially the n individuals in n groups and then to agglomerate the groups. The algorithm of WARD makes it in such a way that the gatherings induce the lowest decrease of R^2 *at each step."*

Line 145- What are "particular characteristics" beyond no-opinion profiles?

A-C Dalcq: We replaced particular by "some" (Lines 151). We hope it makes it clearer.

Line 157- What?

A-C Dalcq: *This sentence is a part of the method. As explained and precised before, we realized a MCA on the seven ideal future farm questions. We observed that the modalities "no-opinion" of the seven questions gathered and were positively related with the first dimension of the MCA. All the modalities reflecting an opinion were negatively related to the first dimension. Thus, the first dimension allowed to differentiate the producers with an opinion or not.*

The modalities "intensive", "global-market", "specialized", "standard quality milk", "employed workforce" and "strongly based on new technologies" gathered and were positively related to the second dimension. The modalities "extensive", "local-market", "diversified", "quality differentiated milk" and "lowly based on new technologies" gathered and were negatively related to the second dimension. The second dimension appeared to us as a gradient between the two extreme models of ideal future farm "Global-based intensive" and "Local-based extensive".

The two dimensions explained almost 95% of the variability of the dataset. Therefore, the study of only these two dimensions appeared to us relevant. Then, we realized a numerical classification on the scores on the two dimensions of the MCA (statistic treatment equivalent to HCPC- Hierarchical Clustering on Principal Components). The first two groups created were the "no-opinion" producers and the producers "with an opinion". This allowed us to exclude the "no-opinion" producers and to study the producers with an opinion thanks to the second dimension, this one had at its extremities the "Local-based extensive model" and the "Global-based intensive model". But the producers with an opinion distributed themselves along this dimension. Thus, we decide to work with the second dimension, as a gradient of ideal future farm (Fig 5).

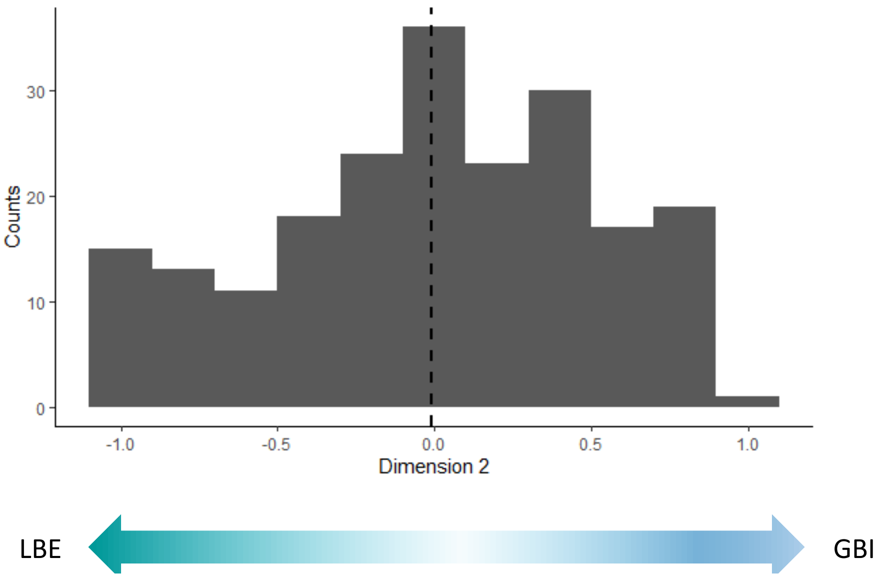

Figure 5 Distribution of the producers with an opinion along the second MCA dimension (N = 207)

Then we want to study the relationships between this ideal future farm gradient and the other information present in the survey.

The relationships between the ideal future farm gradient and the categorical variables of the survey were studied thanks to generalized linear models.

The gradient was the y, the variable to explain. The modalities of the categorical variables were the fixed effect of the generalized linear model, the factors explaining.

$$y = effect + residual$$

Where y was a vector contained the score on the ideal future farm gradient (the second dimension of MCA); effect was the qualitative variables of the survey. In other words, the model was :

$$Ideal\ future\ farm\ gradient = categorical\ variable + e$$

To study the relationships between the gradient and the quantitative variables of the survey, correlation coefficients and their level of significance were calculated.

Line 160- What are the quantitative variables?

A-C Dalcq: Quantitative is a statistical term defining a continuous numerical variable. Do you want that we use the term « numerical » ? But this term is less precise as it does not reflect the continuous dimension of the variable.

Table 1- What are the ns? Maybe a total figure? Percentages?

A-C Dalcq: Absolute frequencies named counts. We precised at lines 186 and 194: "Absolute frequencies (counts)".

How many questions in each dimension?

A-C Dalcq: One question. We have now mentioned that in the Materials and methods section at Lines 187-188: "and of the answer to the question which corresponds to this corresponding characteristic for the current situation".

Line 180- specifics of response rate still missing.

A-C Dalcq: The response rate is 6,1% (Line 200). Which supplementary indication do you need? We gave you numbers of farms with a dairy activity in Belgium and in European Union in this letter. We give also more information about the conditions where the survey was communicated to the producers to give you an idea of the way of proceeding. More information is given at lines 120-126.

Lines 120-126: "We communicated with Walloon dairy producers about the goals of the survey and its access broadly via all communication ways towards them : specialised press, agricultural internet websites, Unions and also advertisements through the milk payment letter which is sent to all the Walloon dairy producers once a month. The survey written in French can be viewed at the following internet link: https://www.gembloux.ulg.ac.be/enquete/index.php/219425?lang=fr and its English translation is viewable in the Appendix"

Table 2- list questions?

We have added the question in the table (Line 223).

**Question**<br>Without taking into account your current farm, what is, according to you, the ideal future farm to ensure a revenue?"	**Proposition**	**Percentage (%)**
Intensive *vs*. extensive	Intensive	43
	Extensive	30
	No opinion	27
Specialised *vs*. diversified	Specialised	43
	Diversified	47
	No opinion	10
Strongly *vs*. weakly based on new technologies	Strongly	35
	Weakly	41
	No opinion	24
Managed by an independent farmer *vs*. a group of managers	Independent farmer	72
	Group of managers	18
	No opinion	10
Family *vs*. employed workforce	Family	87
	Employed	5
	No opinion	8
Providing dairy production for local *vs*. global market	Global	43
	Local	32
	No opinion	25
Providing standard *vs*. differentiated quality dairy production	Standard	38
	Differentiated quality	45
	No opinion	17

Totals

A-C Dalcq: It is now mentioned at line 223.

Figure 1 define dimensions. What are the percentages in the axes labels?

A-C Dalcq: The percentages in the axes labels are the inertia but these ones underestimated the part of information explained by the dimensions. The corrected inertia values were calculated. This is explained in the Materials and methods section at lines 142-146: "For a MCA, the eigenvalue of the dimensions generated, named principal inertia, is a biased measure of the amount of information presented by a dimension (Palm, 2007). Corrected inertia rates were calculated, as described by Benzécri (Benzécri, 1979), to quantify the correct proportion of information of a dimension."

The corrected values of inertia are presented at lines 248-250. The nature of the values are precised in the caption of the figure (lines 251-253).

"The percentage of principal inertia of the dimensions 1 and 2 of MCA were 16.75% and 12.38%, respectively (Fig 1). The value of corrected inertia for the two first dimensions reached 72.7% and 21.5% respectively, gathering almost 95% of the information.

Fig 1. Representation of the modalities in the multiple correspondence analysis first factorial plan. Values of principal inertia reached 16.75% and 12.38%. Values of corrected inertia reached 72.7% and 21.5%."

The correction of Benzécri is made following this calculation:

$$corrected\ inertia = (\frac{s}{s-1})^2 * \left(\mu_k - \frac{1}{s}\right)^2 \ with\ \mu_k > \frac{1}{s}$$

s = number of categorical variables in the MCA

μ_k= eigenvalue of the dimension

(0.33508 and 0.2475, eigenvalues of respectively the first and second dimension of the present MCA)

The corrected inertia gives a better appreciation of the amount of information explained by each dimension (Benzécri, 1979), than the inertia automatically provided by the software.

We did not think necessary to precise this in the paper but we provide the reference where this calculation is presented.

Line 335- where is this figure reference from

A-C Dalcq : We did not provide a representation of the distribution of the producers along the second dimension, we did not think it was necessary as we provided the percentages and this figure would lengthen the paper. But following your request, we have now added this figure in the present manuscript (Lines 376-377).

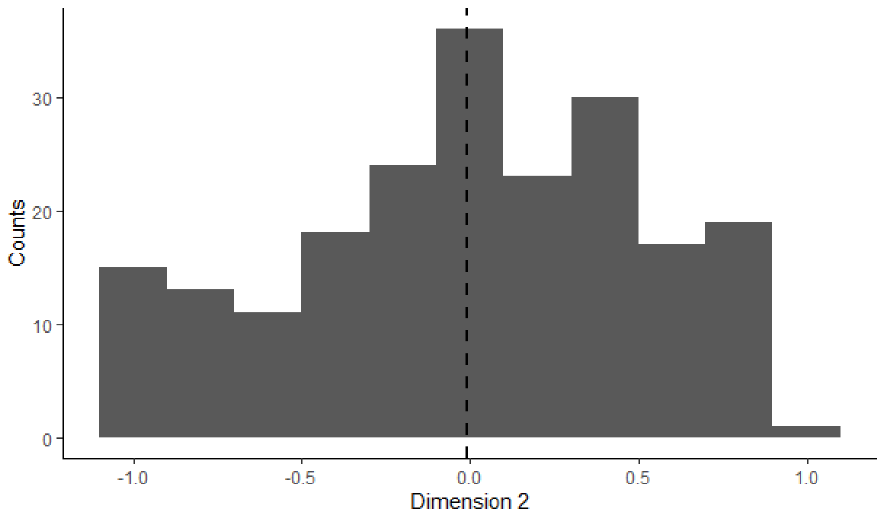

Fig 2. Distribution of the producers along the second dimension (the dotted line represents the mean score on the second dimension of the producers)(N = 207)

Table 5-footnotes? Which is LBI and which GBI

A-C Dalcq : We were not sure about the goal of your question. We precised the function of the letters a and b. Concerning your request about the LBE and GBI, as explained with more details in the point 1/, the analysis is not done between groups LBE or GBI and the variables present in the survey but between an ideal future farm gradient and the variables present in the survey. The means presented in the tables 5, 7 and 8 are the mean value of this gradient for the different modalities of the categorical variables. We explained this at lines 390-395.

Lines 390-395: "Tables 5, 7 and 8 give the results of generalised linear models where the categorical variables were introduced separately as a fixed effect in the model. Significantly lower estimates of IFFg for a specific modality of the considered categorical variable depicts a tendency of producers desiring a LBE model to choose this modality, while significantly higher estimates of IFFg means a tendency of producers wanting a GBI model to choose this modality."

What test is used here?

A-C Dalcq: Generalised linear models were used to study the level of significance of the differences between the means values of the gradient of the modalities of categorical variables: y was the ideal future farm gradient and the effect included in the model was the categorical variable. We explained this part of the method in the Materials and methods section at lines 178-184.

Lines 178-184: "For categorical variables, the scores of MCA dimensions were modelled using these variables as a fixed effect in a generalised linear model. Least squares means were estimated for the two-by-two comparisons using the Tukey test. The level of significance of those differences was assessed based on the P-value of the test. For quantitative variables, Pearson correlation coefficients were calculated between the scores of MCA dimensions and these variables. Their corresponding P-values were estimated to observe if the correlation values were significantly different from 0."

Line 356 explain what they mean by introduced as fixed effect

A-C Dalcq: As explained in the point 1/ of this letter, the gradient was used as y (variable to be explained) of the generalized linear models and the fixed effect introduced in the generalized linear model was the categorical variables. See the previous answer related to the same topic for more details.

Line 620- What is SFI

A-C Dalcq : We precised this at line 661. "SFI = study, formation and information ». It is after the table. We have now added an asterisk to highlight the explanation of this abbreviation.

Reviewer #3: Thanks to the authors, ho made significant improvements to the paper. The full potential of the data is now revealed in the analysis. I particularly appreciate the improvements on the description of the « no-opinion » farmers, as suggested in my first review.

A-C Dalcq: Thank you for the interest given to this study.

Specific comments:

84: add references to « This change implied the disappearance of regulation of dairy supplies and caused volatility and decrease in the milk price »

A-C Dalcq: We added a reference and precised our purpose (Lines 83-84). Thanks for your remark.

Lines 83-84: "This change implied the disappearance of regulation of dairy supplies and was bringing uncertainty about the milk price (16). ~~caused volatility and decrease in the milk price.~~ "

Reference 16: Salou, T., H.M.G. van der Werf, F. Levert, A. Forslund, J. Hercule, and C. Le Mouël. 2017. Could EU dairy quota removal favour some dairy production systems over others? The case of French dairy production systems. Agric. Syst. 153:1–10. doi:10.1016/j.agsy.2017.01.004.

99-100: the question #2 is unclear. « What is the proportion of producers desiring the different IFF? » should be rephrased a bit maybe. The expression « the different IFF » will be vague for the readers. Is the IFF always different from the current farm ? And I guess « their IFF » is more thuitable thant « the IFF » as each respondent will provide a personal definition of their IFF.

A-C Dalcq: We understand your will to make this question clearer. We have replaced it by « How the dairy producers distribute themselves between IFF highlighted ?" (Lines 100-101, lines 365-366).

122: you do not answer to another reviewer's comment, who wanted to know the response rate to the interview. To how many farmers was this survey submitted ? e.g. number of farmers buying the « specialised press », number of advertisements sent with the milk payment letter etc.

A-C Dalcq: We precised in the text that all the Walloon dairy producers received this payment letter: +/- 4,000 producers. Therefore, the 245 respondents correspond to 6,1% of the population, i.e. the response rate. We added information at lines 120-126 & 200-201. We do not know if the information number of farmers buying the « specialised press », number of advertisements sent with the milk payment letter are necessary in this paper but we added information to give an idea of the conditions in which the survey was communicated.

Lines 120-126: "We communicated with Walloon dairy producers about the goals of the survey and its access broadly via all communication ways towards them : specialised press, agricultural internet websites, Unions and also advertisements through the milk payment letter which is sent to all the Walloon dairy producers once a month. The survey written in French can be viewed at the following internet link: https://www.gembloux.ulg.ac.be/enquete/index.php/219425?lang=fr and its English translation is viewable in the Appendix.

A total of 245 producers completed our survey between November 2014 and January 2015."

Lines 200-201: "The sample set of 245 producers represented 6.1% of the dairy producers in Wallonia (about 4,000 dairy producers in 2015 and 3,500 in 2017 (STATBEL, 2019)*."*

240:253 : the description of the no-opinion farmers adds value to the data analysis. However I am not sure that the last sentence is useful. This is your personal interpretation, but the data do not permit to reveal it.

A-C Dalcq: Indeed, this sentence was deleted (Line 279). Thanks for your remark.

Tables 3 and 4: you have two columns, which are « complete sample » and « no-opinion farmers ». A third column which reflects the sample excluding the no-opinion farmers will permit the reader to compare the no-opinion ones with the others.

A-C Dalcq: Indeed, this column was added (Lines 280, 283). And, as asked by reviewer#2, we realized (1) generalized linear models to compare the means of the quantitative variables between the no-opinion producers and producers with an opinion and (2) tests of proportion to compare the proportion of each modalities of the categorical variables between the no-opinion producers and producers with an opinion. We added description of these statistical treatments in the Materials and Methods section (Lines 157-160).

333 : as already said, I think this title is not well written and could be more explicit.

A-C Dalcq: It was changed at lines 365-366. See also the answer to your previous comment related to the same topic.

350 : pigeonholes. Could you be more precise?

A-C Dalcq: As it caused doubt in your comprehension of this idea, we replaced by boxes (Line 384).

- ⇨ *Put people (here dairy producers) in boxes.*

It was used to explain the fact to put a label of someone. But in this paper, we want to nuance the position of the producer regarding its ideal future farm.
