# Supplementary material for: The Walloon farmers position differently their ideal dairy production system between a global-based intensive and a local-based extensive model of farm
Source: PLoS One. 2020 Dec 3;15(12):e0223346. doi: 10.1371/journal.pone.0223346 (PMC7714150; doi:10.1371/journal.pone.0223346)
Supplement: Attachment — Submitted filename: Response to reviewers.docx [file pone.0223346.s003.docx]

Gembloux Agro-Bio Tech, University of Liège

TERRA Teaching and Research Centre

Statistics, Informatics and Applied Modelling

Passage des Déportés, 2

5030 Gembloux

Belgium

22^nd^ March 2020,

Dear PLOS ONE Editor,

In the following of the letter, you are going to find the answers to the points raised by the academic editor and reviewers.

Sincerely,

Anne-Catherine Dalcq

Journal Requirements:

When submitting your revision, we need you to address these additional requirements:

1. Please ensure that your manuscript meets PLOS ONE's style requirements, including those for file naming. The PLOS ONE style templates can be found at <http://www.plosone.org/attachments/PLOSOne_formatting_sample_main_body.pdf> and <http://www.plosone.org/attachments/PLOSOne_formatting_sample_title_authors_affiliations.pdf>

 A-C D : I checked the PLOS ONE’s style requirements.

2. We suggest you thoroughly copyedit your manuscript for language usage, spelling, and grammar. If you do not know anyone who can help you do this, you may wish to consider employing a professional scientific editing service. 
Whilst you may use any professional scientific editing service of your choice, PLOS has partnered with both American Journal Experts (AJE) and Editage to provide discounted services to PLOS authors. Both organizations have experience helping authors meet PLOS guidelines and can provide language editing, translation, manuscript formatting, and figure formatting to ensure your manuscript meets our submission guidelines. To take advantage of our partnership with AJE, visit the AJE website (<http://learn.aje.com/plos/>) for a 15% discount off AJE services. To take advantage of our partnership with Editage, visit the Editage website ([www.editage.com](http://www.editage.com)) and enter referral code PLOSEDIT for a 15% discount off Editage services.  If the PLOS editorial team finds any language issues in text that either AJE or Editage has edited, the service provider will re-edit the text for free.

Upon resubmission, please provide the following:

- The name of the colleague or the details of the professional service that edited your manuscript
- A copy of your manuscript showing your changes by either highlighting them or using track changes (uploaded as a *supporting information* file)
- A clean copy of the edited manuscript (uploaded as the new *manuscript* file)

A-C D :  As I precised after, Cate Chapman, a professional native speaker reviser, revised the modifications of my paper.

3. Please include additional information regarding the survey or questionnaire used in the study and ensure that you have provided sufficient details that others could replicate the analyses. For instance, if you developed a questionnaire as part of this study and it is not under a copyright more restrictive than CC-BY, please include a copy, in both the original language and English, as Supporting Information.

 A-C D : I provided an internet link where the survey is retrievable. The internet link is given in the manuscript.

4. We note that you have indicated that data from this study are available upon request. PLOS only allows data to be available upon request if there are legal or ethical restrictions on sharing data publicly. For more information on unacceptable data access restrictions, please see <http://journals.plos.org/plosone/s/data-availability#loc-unacceptable-data-access-restrictions>.

In your revised cover letter, please address the following prompts:

a) If there are ethical or legal restrictions on sharing a de-identified data set, please explain them in detail (e.g., data contain potentially sensitive information, data are owned by a third-party organization, etc.) and who has imposed them (e.g., an ethics committee). Please also provide contact information for a data access committee, ethics committee, or other institutional body to which data requests may be sent.

A-C D : When the survey was proposed to the dairy producers, it was precised that their answers will remain anonymous and that the results will be presented collectively. If the database was accessible for everyone, it would not respect this commitment. Moreover, the new law about the data protection in European Union stipulates that if the cross of informations can identify an individual, the protection of personal data principle is not respected. This database, with a lot of informations per person, allow the identification of a specific producer. The survey was conducted before this law. But it seems important according to us to respect these new reglementations when considering the supply of the database. The protection data officer of the University of Liège, who adviced me for this area is Pierre-François Pirlet ([dpo@uliege.be](mailto:dpo@uliege.be)). Finally, the data are owned by a committee “le Carrefour des Productions animales”, which gathers stakeholders of the dairy sector (university, research center, unions, breeding association,…) Request of data can be sent to the coordinator, Yves Beckers (yves.beckers@uliege.be) .

b) If there are no restrictions, please upload the minimal anonymized data set necessary to replicate your study findings as either Supporting Information files or to a stable, public repository and provide us with the relevant URLs, DOIs, or accession numbers. For a list of acceptable repositories, please see <http://journals.plos.org/plosone/s/data-availability#loc-recommended-repositories>.

We will update your Data Availability statement on your behalf to reflect the information you provide.

5. Your ethics statement must appear in the Methods section of your manuscript. If your ethics statement is written in any section besides the Methods, please move it to the Methods section and delete it from any other section. Please also ensure that your ethics statement is included in your manuscript, as the ethics section of your online submission will not be published alongside your manuscript.

A-C : A letter from the ethics committee was provided at the first submission. It is precised in this document that no agreement from the ethics committee was required at the time of the collect of the data.

Reviewer #1: Dear authors,

The theme of the paper is very interesting but in my point of view the paper is not good enough at the present moment to be published in this journal. I believe you need more time to improve the manuscript for several reasons. The first is the English. Many parts of the text I could not understand what you want to say including wrong words.

A-C D : The English was revised by a native speaker, Cate Chapman, which is a professional to review the English of scientific papers. The modifications made on the manuscript following your remarks were also revised by Cate Chapman.

Also, I believe that you not formatted the text as the journal guidelines – for example the paper should be on .doc format, not pdf.

A-C D : I don’t understand this comment as the article that I submitted was a .docx document, not a pdf.

The first page with the items is absolutely unnecessary and anyone include this to submit any paper.

A-C D : You are right. I included a table of contents as I understood this demand of PLOSONE in the guidelines but when reading these ones again, I think I made a mistake. This table is not deleted.

Also, the archive missed the number pages after the first table, and the Figure 1 is not included. Is impossible to evaluate your results without it.

A-C D : I admitted I made a mistake by forgetting the line numbers after the first table but not the page numbers. I corrected this in the new version. I don’t understand the problem with the figure as it was included when I submitted the article.

Finally, I am not aware about any good journal that publish results and discussion of a study together. I strongly recommend to separate this section on two sections. You can note that you only comment some results rather than really discuss them. This is why we separate the sections.

A-C D : PlosOne guidelines allowed the organization of the manuscript using a common Results and Discussion section. According to me, it was more suitable for the current article. It allows a progressive presentation of the results and their discussion directly step by step. We have also improved the clarity of this section using questions as proposed by one reviewer.

See the PlosOne below:

**“Results, Discussion, Conclusions**

These sections may all be separate, or may be combined to create a mixed Results/Discussion section (commonly labeled “Results and Discussion”) or a mixed Discussion/Conclusions section (commonly labeled “Discussion”). “

Another general comments:

Introduction: The introduction is too brief in the literature review. I believe the introduction need an improvement showing us other studies to better contextualize the problem because is not clear enough.

A-C D : I have developed what authors found through their researches in the discussion, when I have compared my results to them. In order to limit the repetitions, I have decided to limit the references in the introduction section. Consequently, in the introduction section, I have warned that some authors have already observed trends and highlighted the innovative aspects related to our contribution. However, according to your comment, I have modified some sentences to make them clearer (lines 66-75). Moreover, another reviewer asks me to shorten my introduction by putting more literature review in the discussion. So this also explained my current position.

Introduction: You explain some characteristics that actually would be in the methodology section.

A-C D : Could you mention the lines in order to help my understanding? Indeed, I don’t know where is the problematic section.

Also you say in the introduction that the study was performed just before the end of European milk quota. Do you think this fact impact in your data? How? Please find some place to write about this fact and the impact in your study.

A-C D : As proposed by you, I have completed my idea at the lines 81-85 of this new version. Thanks for your remark, it made my article clearer.

Methodology: The methodology is confused. We do not know for example how the survey was lauched but we have the information about how many farmers were surveyed, that actually could be said in the results. Please start the methodology from the begining of your project.

A-C D : You are right. The methodology about the survey protocol was not enough detailed. All information is now added in the Material and Methods section (lines 109-120). Moreover a new section entitled “Data representativeness” was created in this new version to compile all information regarding the surveyed dairy farmer’s population (lines 178-193).

Some specific comments:

Ln 84: Who “asked”? Please contextualize.

A-C D : It is now precised at lines 64 and 65.

Ln 90-91: It is important to say what they found on these researches.

A-C D : As mentioned in my previous answer to this similar, I have developed with more details what authors found through their researches in the discussion section in order to confront my results. This allows to limit the redundancies throughout the manuscript.

Ln 91: “realized”: I think this word is in the wrong place... did you mean "performed"?

A-C D : You are right, “performed” suits well in this sentence. The change was done at line 74.

Ln 93: Which choices? You did show us which paths they found. The last sentence says nothing.

A-C D : You are right. This sentence was modified at line 75.

Ln 94: You did not present those questions so is bad to say that your study is different. Readers are not aware of these studies.

A-C D : The sentence was modified (lines 77-78). I have never seen this question in other studies but now in the manuscript, I have precised that it is to our knowledge.

Ln 101: Which choice?

A-C D : The choice is now better mentioned (line 87).

Ln 105: “impacts of agriculture and breeding on the environment” Such as…?

A-C D : The sentence was modified (line 91-92).

Ln 105: Which "formation aspect"? What do you mean? I did not understand this entire sentence…

A-C D : The formation aspect concerns the training aspect. The sentence was modified (line 89).

Ln 110: “was” instead “are”.

A-C D : When I spoke about the objectives of my research, I use present tense as these goals are untemporal and multiple.

Ln 112 – 117: This explanation is not necessary here, is like a summary and here is the introduction.

A-C D : I think this statement of objectives are important at the end of introduction to refocus the objectives at the end of the introduction. However, as proposed by the second reviewer, the objectives are now presented throughout questions. This introduction part is now “lighter” as asked by you.

Ln 120 – 121: Usually we say that in the end of the methodology when we are describing the analysis... it is not good here. Please start the methodology from the beginning.

A-C D : I moved this sentence at the end of the material and methods section (Lines 169-170).

Ln 123: But where the study was carried out?

A-C D : : New sentences were added to bring that clearer (lines 109-120).

Ln 123: "a survey"? or "this/our survey"? It means that is another survey that not yours.

A-C D : It is now precised at line 121.

Ln 124 – 125: Why you emphasize this but do not explain the implications of this?

A-C D : As mentioned previously, I have now completed my idea by adding sentences (lines 81-85).

Ln 131 – 132: Bring this to the begining. Again, start from the begining.

A-C D : I have changed the beginning of the Material and methods section accordingly (lines 109-120 & lines 184-185).

Ln 133 – 134: These are results and this paragraph has only one sentence.

A-C D : As proposed, it was moved in the Results and Discussion section (lines 186-187).

Ln 177: Table 1 is showing results so should not be cited here.

A-C D : This reference was done to illustrate the method thanks to the titles of lines and columns of the table. It is to improve the understanding of the method. As no other reviewer made this remark, we have chosen to let this illustration.

After the Table 1 the archive lost the page numbers so I’m not able to do my comments. Also, where the Figure 1? I can not evaluate what you saying without it.

A-C D : I don’t understand the problem as Figure 1 was well submitted. I did not check if the line numbers continue after the first section break. Sorry for this mistake.

Reviewer #2: This study examines dairy farmers' preferred future for their farms, how close their farm is to this ideal, the reasons they arrived at this ideal, and the environmental aspects of the ideal. Using a survey of producers, the author(s) conduct MCA to create a gradient of "ideal future farm" ranging from local-extensive (LBE) to global-intensive (GBI). They then conduct Pearson correlations between this gradient and several key variables (including environmental aspects and decision-making) and finally compare the gradient score (preference) to the producers' current farm characteristics to assess whether the producers have a current farm that fits with their stated ideals.

It is noted that in the memo and manuscript the authors make no mention of having received ethics approval from their institutions for the survey used to collect data from dairy farmers. This must be addressed through correspondence with the editors prior to publication. In the United States, research such as this survey would have needed to receive prior approval/authorization from an Institutional Review Board. I'm not clear what the equivalent would be within the authors countries, but the editors should confirm that appropriate ethical standards were followed including voluntary participation and confidentiality. It appears that the authors have attached some type of letter of confirmation from their home university, which should be reviewed by the editors.

A-C D : In Belgium, five years ago, no agreement from Ethics committee was needed. We have given to the editor a letter of the committee confirming this fact.

The study is quite interesting and could make a valuable contribution with some important changes and improvements. The statistical analyses seem to be used appropriately throughout and the findings are quite interesting. In particular, the authors' discussion of how farmers arrive at their IFF and the relationship to environmental factors is enlightening. With revisions, the paper could have quite an impact. I raise my concerns for revision below in order of priority.

A-C D : Thanks for your opinion about the qualities of my paper.

Firstly, the authors must add much more methodological detail to their discussion of the survey. Specifically, they must address how the sample was obtained, generalizability, how the survey was actually administered (mail? online? etc.) and why that was appropriate, response rate (and non-response analysis), pre-testing, etc.

A-C D : You are right. This information was lacking. More details are now mentioned in the new manuscript (lines 109-120).

They should also discuss how missing data and "no opinion" was coded and treated in the MCA and GLM.

A-C D : Only surveys completely answered were analysed in this research. Therefore, we had no missing data. This is now precised (line 121). Each question proposed the two contrary choices and the No opinion proposal. As explained at lines 144-145, cluster analysis was used to exclude the group of producers with particular characteristics. This was done to remove the producers with a No-opinion profile (cf Figure 1 and lines 235-237). So, these producers were not taken into account for the GLM and Pearson correlation analyses.

Secondly, it is not clear to me why the authors use MCA instead of Latent Class Analysis. The goals of the study - to group farmers/respondents into classes - and the language that the authors use to discuss the MCA results feels more appropriate to LCA. Is there a specific reason they did not use LCA? If not, I would encourage them to consider using LCA or at least running LCA as a robustness check.

A-C D : The first goal of the article was to study the relationships between the modalities of the seven IFF sub-questions. Some sentences were added to better highlight this point (lines 133-134). Latent class analysis does not allow that. The idea was not to force the creation of groups of producers. Then two gatherings of modalities were observed. But, there was a will to not put producers into a pigeonhole but speak about the producers to have a tendency to be for a model or another. Some information was now added in the text to improve the understanding (lines 348-349).

Thirdly, it is not always clear how the concepts were actually measured in the survey. Some of the core concepts are quite complex, and the authors do not tell us what they actually asked the farmers and how they got from those survey questions to the concepts that they coded. Either a link to the survey text somewhere online or an appendix to provide more detail on the actual measures is necessary. Things like intensive/extensive, environmental aspects, are complex concepts and it needs to be clear how they were actually measured and why they were coded as simple binary variables.

A-C D : I put more details about the survey and its internet link in the Material and Methods section (lines 109-132). Intensive/ extensive were proposed without other explanations. Even considering bias, it give an insight of what is the tendency of the producer regarding this question.

There are two relevant rural sociology literatures that the authors should explore: one regarding the contradictory class location of family farms and one regarding bifurcation of farm systems. The first should start with the book My Own Boss by Pat Mooney and those who cite him. Mooney discusses how farmers exist as both workers and capitalists (employ others) and this literature would complicate the family/employee line the authors are trying to draw. The second literature describes the divide occurring in agriculture between large-intensive farms and small-local farms, calling this process bifurcation. This would bolster the authors' argument to engage with this literature.

A-C D : Thanks to inform me about these researches. I have explored these two literatures and implemented the contents of those article in this new manuscript (lines 215-217; lines 338-341; lines 538-541). Concerning bifurcation, I did not find articles about bifurcation between large-intensive farms and small-local farms as you said but I read phenomenon of bifurcation for the organic sector (lines 338-341).

Regarding the writing, I would encourage the authors to revise the introduction to set up their research using questions rather than statements. This would make it much more clear to readers what the aim is of their study. I have provided some specific notes and suggestions in the PDF.

A-C D : I have changed the end of my introduction to present the goals of my research using research questions (lines 97-104). Thanks for this suggestion and your proposals, it made the article topic clearer. For more clarity, I have also changed the titles of the different parts in the Results and discussion section using these same research questions.

Also, in the results section, there is much that should be removed and moved to the discussion and conclusion. The comparisons to other studies should be saved for the discussion and conclusion, not results. It makes it difficult to grasp your key findings (particularly when so much is covered) when you are constantly delving into other literature. That literature should be saved for a discussion section.

A-C D : We have decided to write a common Results and Discussion section because according to me, it was more suitable for my article. Indeed, this allows a progressive presentation of the results and their discussion directly step by step. I preferred to address each theme one by one as they are quite different but they followed a logic stream. However, I have changed the titles of this part based on the formulated research questions as you proposed in your past comments to improve its clarity.

It is a small, but important, note: the authors repeatedly use the term "qualitative" to describe some of their variables. These measures are not qualitative, they are categorical or nominal. Within social science research, qualitative has a specific meaning (meaning data derived from text, interviews, observation, etc.) and they should avoid using that term when that's not really what their data entails. Throughout the manuscript, this should be changed to "categorical", "nominal", or "binary" when appropriate.

A-C D : Thank you for this comment. I used “qualitative” as a contrary of “quantitative”. I was not aware that in social science research it could have another meaning. In the current manuscript, the term “qualitative” was replaced by “categorical”.

Abstract : MCA is like PCA but for binary variables. The only potential concern is the overly simplistic representation of the concepts with binaries.

A-C D : MCA shares a similar concept with PCA about the dimensionality reduction. However MCA is based on frequencies of modalities. However, it is not true to mention that MCA is for binary variables. Categorical variables can be also used in this kind of method reducing the dimensionality of X matrix. MCA was appropriate to this study as we don’t want to group farmers but we want to observe their tendency to belong to one or another group. It is why we have used the concept of gradient.

Abstract : The only concept here that's not immediately interpretable by me is the intensive versus extensive

A-C D : As mentioned in a previous comment, Intensive or extensive were proposed without other explanations. Even considering bias, it gave an insight of what is the tendency of the producer regarding this question.

Ln 61 : These two sentences do nothing to build the study up and are inflammatory (and probably inaccurate).

A-C D : We consider that these two sentences have sense because it reminds the sense of the profession of producer. It is a profession amongst others but which provides a basic need of the society. And this profession is at a turning point with its separation between two models at our time, as observed in the article. I do not think it is inflammatory, I added references to objectify this phenomenon (line 43).

Ln 69 : Quite a bit of this background here before getting to your actual questions. I would encourage you to frame your questions and study earlier.

A-C D : I am not sure to have understood your comment. However, the current manuscript is now reformatted based on research questions as proposed in one of your previous comment. The structure is now clearer.

Ln 95 : So the ideal type is only revenue specific? Or does it encompass other goals for the farmer and farm?

A-C D : The question was asked following this idea. It is now precised in the manuscript (lines 124-126).

Ln 101 : The wording here is not clear. "This choice" meaning the choice of an ideal? Be more specific in the wording.

A-C D : This sentence was rewritten (line 87).

Overall, the abstract does a much better job of setting up the study than this introduction is doing. What are your questions? Stating them as research questions will make it much more clear.

A-C D : As mentioned previously, it is now the case. Thank you for this proposal.

Ln 103 : Not clear what is meant by "formational"

A-C D : This sentence was rewritten (line 89).

Ln 109-112 : This is much more clear. Again, stating the research questions as QUESTIONS would help. What are dairy farmers "ideal future farming systems" (IFF)? How do farmers decide on their IFF? How do environmental aspects factor into IFF decisions? And, ultimately, how do farmers' IFF compare to their current farming systems?

A-C D : You are right. The manuscript is now re-structured using research questions. This improve greatly its clarity.

Introduction : Break the introduction and the literature review into discrete sections to allow your questions to be more clear, your case set up (this comes in introduction) and then give more depth on the studies that you are reviewing.

A-C D : The introduction was voluntary short to limit the redundancies with the discussion section. I have developed in the details the past studies in the discussion section in order to confront my results. However, now the end of this introduction was revised and the objectives were mentioned using research questions (lines 97-104) as proposed by you in one of your previous comments.

Ln 123 : Discuss the representativeness of the sample. From this group, can you generalize to the Belgian producers generally? What was the sampling approach? Was it random? What was the response rate to the survey, and did they conduct any non-response analysis to confirm that there is no bias? Ln 126 : They must describe this methodology in much greater detail. How was the sample obtained? Response rate? How was the survey administered? Was it pre-tested? Who on the farm actually filled out the survey? All of these things must be discussed.

A-C D : You are right. The description was not enough complete. Information were now added in the manuscript (Lines 109-122).

Ln 136 : They are all quantitative, it's just that some are categorical.

A-C D : Among all variables present in the survey, some of them were not quantitative. They measured not a quantity but inform on a quality.

Ln 142 : What about other types of missing data, such as skipped questions? How was this dealt with?

A-C D : We had no problem with missing data as only surveys fully completed were kept for this analysis. It is precised in the manuscript at line 121.

Ln 142 : How was this coded? Were these responses dropped out of the MCA and GLM?

A-C D : Details are given at lines 133-135 & 235-239.

Ln 147 : Again, not qualitative: categorical (or nominal). Qualitative has a specific meaning, and you should not use it this way.

A-C D : As mentioned previously based on your previous comments, the term “qualitative” was replaced by “categorical”.

Ln 150 : Did the authors explore using latent class analysis? Why or why not? It seems that LCA might be more appropriate than MCA for what the authors are trying to do which is classify respondents into categories.

A-C D : See the previous answers to your comments about the comparison between MCA and PCA as well as the interest of using MCA to define the gradient.

Ln 152 : If you want to use the term "classes" to discuss the grouping of the respondents, than Latent Class Analysis seems much more appropriate than MCA.

A-C D : Almost 95% of the variability is explained by the two first dimensions. The first separation of the cluster analysis was between the No-opinion and the other producers, which permitted to identify and isolate them. The second separation would be along the second dimension, the creation of classes encountered this objective. As mentioned previously, the latent class analysis was not suitable for our study.

Ln 166 : How did you determine which variables to test the relationship with?

A-C D : We have tested the relationships with all the variables related to the chosen themes (i.e., reasons, environmental aspects and training). Only variable with significant relationships with the IFF gradient are presented in this article. However, we have decided to present the results about two variables (i.e., age and geographical situation) which were not significant because we found them interesting for the interpretation.

Ln 180 : It would be helpful to provide a link to the actual survey text (perhaps translated) or more detail on the actual questions asked that became the core concepts. For instance, how were farmers asked about "intensive" versus "extensive"? These are complex concepts and not everyone would agree on the same meaning.

A-C D : The internet link of the survey is now added in the Material and Methods section (line 120). Intensive/ extensive were proposed without other explanations. Even considering bias, it give an insight of what is the tendency of the producer regarding this question.

Again, need to provide us with information about how these were actually measured. What did you actually ask farmers to get to these concepts? Why are these complex concepts represented only as binary variables? Particularly for questions about someone's IDEAL, you need to tell us what you actually asked them

A-C D : The link of the survey is now available at line 120. Sentences were added to precise the way of these questions was proposed (lines 124-131).

This especially seems problematic. Don't most farms use combinations of both family and non-family? Is the family meaning NO employed labor? This is why you need to give us more detail, probably as an[…] [the sentence is not complete in the comment]

A-C D : The questions were voluntary sliced to have their point of view (more a system or another?) in order to know what they consider as ideal.

There is a related rural sociology literature on the fact that family farmers are BOTH workers and employ other workers. You should look for pieces by Mooney (My Own Boss) and others who cite him

A-C D : Thank you for this suggestion. I have explored this literature and implemented the contents of this article in the new manuscript (lines 215-217; lines 538-541).

It is strange to mix the review of other studies into the results section here. This is all discussion and conclusion, not results.

A-C D : As mentioned previously, we have decided to write a common Results and Discussion section because according to us, it was more suitable for this article. It allows a progressive presentation of the results and their discussion directly step by step. I preferred to address each theme one by one as they are quite different but they followed a logic stream. However, the structure is now clearer thanks to your suggestion to use research questions.

The authors should look at the BIFURCATION literature and use that term.

A-C D : As mentioned previously, I have explored this literature and implemented the content of these articles in the manuscript (lines 338-341).

This table is quite hard to read as a whole. Perhaps subdivide or trim?

A-C D : It counts for me that it is in a whole because there are all the variables that I identified as reasons of the choice of IFF. As other reviewers made no remark, we have decided to let it like that.

Here add more substantive interpretation of the correlations. Give us the meaning, not just reading the table.

A-C D : The meaning of the correlation is explained in the following lines, with the interpretation of relationships with other variables depicting the same phenomenon. According to me, it is an interpretation and not only a reading.

“The choice of GBI model could be explained by this current workload, involving the need for an increase of revenue. So, the solution considered could be higher milk production and the breeding of more cows rather than diversification of activities and self-valorisation activity, the development of which requires a lot of time. Samson *et al.* (2016) [14] confirmed this in the Netherlands by highlighting a nearly significant effect of labour productivity on the DP increase strategy.”

Reviewer #3: The paper is clear and pleasant to read. Reporting the results of a survey on the ideal future farm for Walloon dairy farmers, this empirical research sheds light on the (ex ante) attitudes of farmers. The methods used are simple and do not add value, but they are clearly explained and used correctly. The results of the analysis are clearly explained. I would like to stress that a major effort has been made to compare the results with those of other neighbouring areas (France etc.). The comparison is of particular interest to the reader, as it allows us to situate this research work in relation to the existing literature, but also to underline the originality of the work and the new knowledge provided by this applied work on the aspirations of dairy farmers in Wallonia .

A few remarks:
- The analysis of the survey focuses on farmers' responses to questions regarding their perception of the ideal future farm. On page 13, it is written that "no opinion" farmers are excluded from the analysis to avoid potential bias. However, I would have liked to have seen a specific paragraph in which these "no opinion" farmers are analysed, even if only briefly. For example, is the answer 'no opinion' characteristic of a particular type of farmer? Furthermore, the proportion of these respondents is itself an indication of the lack of perspective that some dairy farmers may feel. The analysis of this sub-sample can therefore complement the overall analysis.

A-C D : Frequencies and means of variables for the no-opinion producers are now added. We have also performed a comparison with the complete sample to characterize this group (lines 239-258). Your second remark is really interesting. Indeed, 15% of producers may miss of perspective. I saw this conclusion in no other studies. I tried to explain this phenomenon thanks to the results observed.

- The MCA is carried out correctly, and the conclusions you draw show that there are two main types of farmers, both in terms of their current activity and their vision of the ideal future farm. It would have been interesting, and it is advisable, that you supplement your analysis with a hierarchical clustering, whether divisive or agglomerative. Such an analysis will provide a solid complement to your data processing and could corroborate and even support your conclusions.

A-C D : It was deliberately chosen to not create groups, to speak about tendency for a model or the other, producers being more or less convinced by the two extreme LBE and GBI model. To quantify the producers for a model or another, we have created classes along the dimension 2 which reveals to be a gradient between the two extreme ideal future farms. Moreover, almost 95% of the variability is explained by the two first dimensions. The first separation of the cluster analysis was between the No-opinion and the other producers, which permitted to identify and isolate them. The second separation would be along the second dimension, the creation of classes encountered this objective.

- The main conclusions of the analysis highlight dairy farmers' vision of the future of their sector. As written in the text, the survey dates from 2015. Is it possible to compare the results with more recent statistics on the development of dairy activity in Wallonia? In other words, are there data to make a comparison between ex ante attitudes and ex post behaviours?

A-C D : It is an interesting remark. Indeed, those findings motivated us to make the survey again, five years later. Dairy producers are answering the survey at this time.
