# Supplementary material for: The Walloon farmers position differently their ideal dairy production system between a global-based intensive and a local-based extensive model of farm
Source: PLoS One. 2020 Dec 3;15(12):e0223346. doi: 10.1371/journal.pone.0223346 (PMC7714150; doi:10.1371/journal.pone.0223346)
Supplement: Attachment — Submitted filename: Response to reviewers.docx [file pone.0223346.s004.docx]

Dear Academic Editor, dear Reviewers,

First, I would like to thank you for the time spent to improve the understanding of this manuscript. This letter is organized in two parts. The first part deals with the main issue of the review (i.e., the method mobilized, which corresponds to the point raised by the academic editor and one remark of the reviewer). The second part concerns the specific answers to the specific remarks formulated by the reviewers.

Sincerely,

Anne-Catherine Dalcq

1/ Methodology

Please read carefully the propositions of  reviewer #2 especially about the recommendations you did not took into account in your revision. I am afraid the reviewer is right about the interest of LCA vs MCA, as we expect a characterization of different profiles of dairy farmers.

But my proposition is that instead of LCA, you use HCPC (Hierarchical Clustering on Principal Components) which a natural and standard extension of MCA (see Arguelles et al. 2014,  Kassambara 2017 or the following technical report <http://factominer.free.fr/more/HCPC_husson_josse.pdf>).

*H. Soyeurt :*

Dear Editor,

Dear Reviewers,

Instead of my PhD student, Miss Anne-Catherine Dalcq, I would like to answer to the question related to the methodology used in this study. I am Professor Hélène Soyeurt and I teach courses related to Data Mining and Machine Learning at Gembloux Agro-Bio Tech (University of Liège). Therefore, I have an experience in the use of multivariate analysis. It is why I would like to answer by myself to the question related to the method used in this article.

Before starting an explanation, I would like to precise that we have not really understood the comments of the reviewer about Latent Class Analysis (LCA) because the objective was not to create groups of farmers. Indeed, and this is the innovative aspect of this paper, a gradient between two quite different models of farms was studied. The gradient is really important because it appears to us simplistic to classify farmers in only 2 groups, working with a gradient allowed us to nuance the position of the dairy producers and to analyze more precisely the link between this position and other characteristics. The use of the gradient allows studying the trend of a farmer. It is why in this paper, we always mention “tend towards” to make a reference to the position on the gradient and not a binary choice of a model.

As we did not want to create farmer’s clusters, LCA was not appropriate as well as HCPC. However, as asked by the second reviewer to prove the robustness of our approach, we have decided to show you the similarities and the extended work that it is possible to do using Multiple Correspondence Analysis (MCA) and LCA. Indeed, it is also possible to use LCA to create a gradient instead of using the clusters.

So, I would like to remind the methodology that we have proposed in this study (see the figure below, Fig 1)). Again, MCA was used to observe the relationships between the seven studied variables. Based on the interpretation of MCA dimensions, we have observed that the second dimension represented the positioning between the two models for a dairy farm. Therefore, the score for this dimension for a specific producer allows to know its perception of ideal future farm between the two extreme models. This gradient allows avoiding to limit the farm typology to 2 clusters. This is interesting because many farmers combined some approaches specific to one model of farms (no more intensification but extensification, local-based market) or another one (global-based market, continuously improvement of the productivity thanks to, notably, intensification, …). Therefore, wishing to split the dairy farmers into 2 groups is too limitative. Some farmers remain between these two models (Fig 2), some farmers are more convinced than others by a model. Farmers showing higher scores are really convinced by GBI model, farmers showing lower but positive scores choose GBI model but are not 100% in this way of farm development. Negatives scores express the position of farms in favor of LBE model, the lowest scores reflected pronounced adhesion to this model. It is why the gradient as proposed in this paper was useful and seem to us the most interesting tool to represent dairy producers.

MCA

245 responders

7 variables

MCA dimensions explanation

Representation of individuals

Deletion "No opinion"

MCA combined with Ward clustering (equivalent to HCPC)

Definition of a cluster related to "No opinion" included 38 respondees

Cleaning of dataset ==> 245 - 38 = 207 records

Gradient

Positioning between the two models was represented on the second dimension of MCA

Use the score of MCA to characterize the position.

Figure 1 Methodology

Figure 2 Distribution of the producers (the "with an opinion" ones) along the second dimension of the MCA

Similarly, we have done this job using LCA methodology as requested by the reviewer#2. LCA allows defining clusters from the dataset. In this case and to be in line with the study objective, we have decided to create 3 clusters. Moreover, based on the AIC and BIC values, it was also the best model (Fig 3). LCA allows us to create those 3 clusters. After the interpretation of defined clusters, it appeared that we have a cluster representing “No opinion” responders, one cluster representing “global-based intensive” (**GBI**) farmers and one cluster representing “local-based extensive” (**LBE**) farmers (Fig 4). Therefore, those clusters were similar to the ones obtained by combining MCA and Ward clustering (=HCPC method) as done to clean the dataset (i.e., extract responders with “no opinion” behavior). Again, we did not want to use those clusters to make our analysis. But, using this methodology, it is possible to obtain a probability to belong to a specific cluster. Therefore, we have decided to compare the gradient as defined in the current study to the probability to belong to the LBE or GBI clusters defined using the LCA method.

So, now, it is time to present you the results. I will not present you the results about MCA as those results are reported in the article. In this paragraph, we will focus on the LCA results. As we used categorical variables which are not ordinal, we have decided to use the polytomous latent class analysis. The variables used, called manifest variables, were the same than the one used for MCA. The modalities for each variable were recoded from 1 to 3:

- Intensification: 1. Intensive, 2. Extensive, 3. No opinion
- Number of activities: 1. Specialised, 2. Diversified, 3. No opinion
- Technology: 1. Strongly based on new technologies, 2. Weakly based on new technologies, 3. No opinion
- Workforce: 1. Familial workforce, 2. Employed workforce, 3. No opinion
- Kind of management: 1. Group of farmers management, 2. Independent farmer management, 3. No opinion
- Market: 1. Global market, 2. Local market, 3. No opinion
- Milk quality: 1. Standard quality milk, 2. Differentiated quality milk, 3. No opinion

As this clustering is very sensitive to the prior values used to start the iteration, 10 repetitions were used to provide you the final results for all models. All calculations were done with R software and more specifically the package poLCA. First, we have run different LCA models using a different number of classes (clusters). We have tested models from 1 to 10 classes and then we have estimated the AIC and BIC criteria to observe which model allowed the best fitting. The results of AIC and BIC for all models are presented in the figure below (Fig 3). From this figure, we can conclude that the model allowing the creation of 3 classes is a good comprise between BIC and AIC (i.e., the lowest BIC and AIC values).

Figure 3 AIC & BIC criteria values

The figure below shows you the clusters defining by the model allowing a discrimination of the data into 3 clusters (Fig 4). This figure represents the probability to have a specific modality for each cluster. So, based on those results, we can conclude that the first clustering (class 1) is related to GBI dairy producers, the second cluster (class 2) is related to LBE dairy producers and the last cluster (class 3) is related to the dairy producers with “no opinion”.

Figure 4Description of the three clusters obtained by LCA method

Therefore, in order to clean the dataset (i.e., delete records from farmers having many “no opinion” views), we can use the clustering 3. This process is similar to the MCA + Ward clustering (HCPC) proposed in the manuscript. Again, we did not want to use clusters but we want to use a gradient. For MCA, this gradient was the second MCA dimension. In the context of LCA, this gradient can be derived from the probability to belong to class 1 (“GBI producer”) or class 2 (“LBE producer”). So, to show the robustness of the MCA approach used, we calculated the correlation between those probabilities and the score of the second MCA dimension (called gradient in the manuscript). The correlation between the probability to belong to class 1 and the gradient was equal to 0.83. The correlation between the probability to belong to class 2 and the intensification gradient was equal to -0.87. The correlation between the 2 probabilities was equal to -0.97.

From all of those results, you can see that the relationship is strong between MCA and LCA using an innovative approach focusing on the score/probability of an individual and not directly to a cluster. Using only the second dimension of MCA, we can reflect both clusters (class 1 and class 2) simultaneously. Indeed, the second dimension is a gradient “GBI-LBE” and included both. This is really interesting to observe the relationships between this gradient and other quantitative or qualitative variables as now the studied trait is quantitative. It allows to see if a modality of a categorical variable is the choice of really convinced GBI dairy producers or dairy producers only sticking out of the GBI model.

I hope that this demonstration illustrates well the relevancy of the approach proposed in our paper. A sentence will be added in the materials and methods section to explain why LCA was not used. Moreover, some articles exist also in the literature to prove the mathematical relationships between MCA and LCA (e.g., Lautsch and Plichta, Psychology Science 2003:298-323 as well as Van der Heijden, et al., Sociological methodology 1999).

The fact that the two dimensions explained almost 95% of the variability and that all the modalities representing an opinion positioning themselves along the second dimension support us to take the second dimension as gradient, after deletion of the No-opinion producers which were discriminated by the first dimension.

Finally, I would like to acknowledge you for the great job done in the review of this paper. The manuscript was improved a lot.

Sincerely,

Prof Hélène Soyeurt

PS : The answers to all other comments were done by Anne-Catherine Dalcq.

2/ Others comments

**Reviewer #2: In this revision, the authors have significantly improved the English language translation and clarified their research questions, but they have failed to address the shared concerns of reviewers regarding methods and clarity of writing. They have not adequately addressed the substance of the comments from reviewers.**

**Regarding methods, the reviewers still do not offer a compelling and clear justification for why MCA is appropriate for their goals and they do not offer the latent class analysis that I suggested or the hierarchical clustering (related) suggested by reviewer 3 as alternatives or robustness checks. MCA, while performed adequately, is not well suited to the way they discuss their results. They continually refer to “types” of respondents, which is what latent class analysis is for. MCA is about identifying clustering of variables, not clusters of respondents. All of their interpretation of results is about clustering and patterns of PEOPLE, not variables. This indicates a significant misalignment between the method and the research goals.**

*A-C Dalcq: First, we would like to thank you for the deep reading done on this article and for your formulated comments. We missed your request to test the robustness of our method with other ones. We apologize for this mistake. You will find our work of comparison with LCA method in the first part of this letter. In the article, we speak about producers tending towards “GBI” or “LBE” models to express the results coming from the use of our gradient. Indeed, MCA is a method to identify relationships between variables but is also suitable to make groups of individuals thanks to the use of its extension HCPC, as proposed by the academic editor. A detailed information about the comparison of MCA and LCA is now given in the first part of this letter.*

**The authors do not adequately discuss the two dimensions of the MCA (figure 1) and the axes are not adequately labeled. It is not made clear why the second dimension is retained.**

*A-C Dalcq: We added some information. The updated explanations of the two dimensions are taken up just after (Lines 254-261/286-306/339-343). Do you need more information? If it is the case, could you guide us?*

*Remark : Lines specified throughout this letter are those of the revised manuscript with track changes.*

*The axes are labeled as the figure is provided by SAS 9.4. We precised its meaning thanks to the caption and the following interpretation.*

*Explanations of the two dimensions:*

*Lines 254-261: “The first dimension of MCA showed positive relationships with the modalities no opinion of each characteristic and negative relationships with all the modalities representing an opinion. Thus, the first dimension of the MCA allowed ~~permitted~~ differentiation between the producers who did not give their opinion concerning characteristics of IFF and the producers who did (Fig 1). Cluster analysis was used to isolate the group of producers with a lot of ‘no opinion’ answers to the seven questions: this formed the first separation of classes of the analysis, dividing the “no-opinion” producers (15%) from the others (85%).”*

*Lines 286-306: “The second dimension of the MCA showed positive relationships with some modalities of the IFF characteristic and negative relationships with their opposite. Thus, this dimension seems ~~was the most interesting for highlighting~~ to highlight the wishes of dairy farmers about their IFF, for those who took a position on this question. More precisely, this axis showed a gradation of question modalities and proximity between several characteristics. This dimension led to the identification of two extreme tendencies (Fig 1); the modalities of familial workforce, independent farmer management and management by a group of farmers were near to zero on this axis (Fig 1). This means that the small proportion of producers supporting group management was distributed between the two extreme tendencies observed. The position of the modalities of familial workforce and independent farmer at the middle of the second dimension illustrated the fact that these modalities were chosen by producers from the two tendencies identified. The small proportion of producers choosing an employed workforce was positioned at the top of the second dimension (Fig 1).”*

*“The first tendency, related to high scores on the second MCA dimension, corresponds to IFF with the following characteristics: global market, standard milk, intensive system, employed workforce, specialised and strongly based on new technologies.”*

*Lines 339-343: “The second tendency, contrary to the first tendency, was characterised by negative scores on the second MCA dimension. This axis was represented by the following modalities: weakly based on new technologies, diversified, differentiated quality milk, local market and extensive system (Fig 1). This reflects another form of dairy farming. »*

*Reason of the use of the second dimension:*

*Lines 379-384: “To study the relationships between the different IFF, the reasons for these and other interesting technico-economic information, the second dimension was considered as a gradient (****IFFg****) interpreted at the extremities as global-based intensive producers (GBI: high positive scores) and local-based extensive producers (LBE: high negative scores). The choice to work with a gradient rather than a clear separation of the two tendencies was motivated by the will to not put dairy producers into boxes ~~pigeonholes~~”*

**While the authors have provided a link to the survey online, that link is only in French and requires registration with an email address before it can be viewed, so that is not adequate. The authors still have not addressed the real concern that I raised in my previous review: they need to be clear about how they measured their concepts (intensive/extensive, etc.), what specific survey questions were used, and how those variables were coded. A clear list of questions for each concept and the coding for each is needed. For instance, in Table 2, it is not clear what specific survey questions or variables represent these concepts and how those variables were actually coded.**

*A-C Dalcq: In the current version, we have added an annex (Appendix 1) with the translation of the questions mobilized in the paper. Given the length of the survey, we provided only the questions raised in the present paper. Some information are mentioned at lines 129-136 about the questions related to the ideal future farm characteristics (intensive vs. extensive,…), the way of measurement for our developed concepts. If you need more information, could you precise them to us explicitly ?*

*Lines 129-136: “The entire survey was composed of 127 questions where the answers were decomposed into 498 categorical and 44 quantitative variables. The question ‘Without taking into account your current farm, what is, according to you, the ideal future farm to ensure a revenue?” was proposed to the producers and they must ~~could~~ choose between short propositions on seven items: 1) intensive or extensive production; 2) specialised or. diversified activity (or activities); 3) farming strongly or weakly based on new technologies; 4) farm managed by an independent farmer or a group of managers; 5) family or employed workforce; 6) providing production for local or global markets; 7)providing standard or differentiated quality production. The modality “no opinion” was available for each IFF question.”*

**The authors still do not address response rate for the survey. They do now address representativeness of their respondents for this specific region, but they have not addressed the bigger questions of representativeness: how does this one region in one nation represent that nation, Europe, and/or agriculture broadly?**

*A-C Dalcq: The response rate of 6,1% was already precised in the past manuscript. You can find it at lines 200-201 of the current text.*

*Lines 200-201 : “The sample set of 245 producers represented 6.1% of the dairy producers in Wallonia (about 4,000 dairy producers in 2015 and 3,500 in 2017* (STATBEL, 2019)*).”*

*For the second part of your comment, do you want that we precise that the Walloon Region is one of the two regions of Belgium, which is one of the 27 members countries of the European Union? If yes, the proposal could be : “The Walloon Region is one of the two regions of Belgium, which is one of the 27 members countries of the European Union”.*

*Or do you want that we precise the number of dairy producers in Belgium and in the European Union? The International Dairy Federation mentions 9,674 dairy farms in Belgium and 1,130,700 farms with a dairy activity in the European Union* (Confédération Belge de l’Industrie Laitière, 2020)*. The number of Walloon dairy farms is obviously low amongst all of these countries. We do not know if all this information is relevant to be written in the manuscript.*

*Complete reference:* Confédération Belge de l’Industrie Laitière. 2020. Rapport Annuel 2020.

*The goal of this paper is to inform about the position of dairy producers of a region, which is moreover quite heterogeneous regarding the geopedologic conditions (lines 205-206), this one can represent the context and the resources of other producers in Europe. The goal was not to give a complete vision of all the European producers, which needs higher means.*

**In all tables the n, or respondent totals, should be clear.**

*A-C Dalcq: We added a sentence at lines 395-396 :“These analysis were conducted on the producers who have an opinion (N = 207).” And the N was precised and added in each table. Thanks for this remark which brings clarity throughout all the paper.*

**For Table 5 there are subscripts/footnotes that are never defined or labeled.**

*A-C Dalcq: The subscripts are now precised in each table. “Means with different letters are significantly different.”. Thanks for this remark.*

**For Tables 3 and 4, no significance tests are reported.**

*A-C Dalcq: Indeed, the goal was not to test the differences between the no-opinion producers and all the sample but to give an idea of the characteristics of the No-opinion producers. Reviewer#3 asked us to give all of this information also for producers with an opinion. We realized ANOVA tests between the no-opinion producers and the producers with an opinion (lines 280-284)*

**The interpretation of the MCA results is circular logic. They define the clusters based on variables such as the attitude toward technology and then present a finding that people who are in the “pro-technology” GBI cluster have more positive attitudes towards technology. Of course, that is how you defined the scale in the first place.**

*A-C Dalcq: The variable “technology” is “Mechanisation and robotisation : help for workload and administrative aspects “ and is present in the part “Reasons”. This result is presented to explain one reason of the producers tending towards “GBI-model” to tend to this model and one of its component, the technology. We have better precised our idea by adding the sentence: “We observed that the wish of technology of producers tending towards GBI model can be explained by the fact that they considered it as help for workload.” at lines 534-536.*

**Regarding writing, the presentations of results and its mixing with discussion of existing literature is still extremely unclear and difficult to follow. Both reviewer 1 and myself raised this critique: presenting your results intermingled with other literature is difficult to read and makes it unclear what your key findings are. This is not about the technical requirements of the journal. In its current presentation, readers cannot easily identify what your key findings are in each subsection and it is very difficult to read. For instance, in the section on pages 15-16, the authors spend substantially more time discussing other studies than they do their own results.**

*A-C Dalcq: As already mentioned and visible at lines 304-364 in the revised paper, the explanations based on the findings of other past studies help to explain the relationships observed between the modalities of the seven ideal future farm characteristics.*

**The introduction is improved, but still weak. The first paragraph is overly general and does nothing to build the focus of the paper. The authors also spend too much time asserting the contribution of their study before they have even reviewed the literature or told us what their analysis will be.**
*A-C Dalcq: What could be your expectations about the structure of this introduction? As the reviewer#3 did not make comments about the redaction of this part and without deeper expectations from your, the structure of the introduction was not changed.*

**The attempts to incorporate new literature are cursory.**

*A-C Dalcq: The work of Mr Mooney was consulted and two references was added to the paper (lines 236-238, lines 577-580). Did you expect references to more elements of his work? Could you precise which ones? Moreover, we also investigated the phenomenon of bifurcation. Literature about bifurcation was mainly found for the organic activity. We consulted a Professor with skills in sociology, Prof Kevin Maréchal, of Gembloux Agro-Bio Tech-University of Liège (Belgium), who provided us also this literature reference. Do you have other literature to advice to me?*

**The paper is not appropriately written for a general audience. They assume too much prior knowledge from readers regarding methods both methods and the case.**

*A-C Dalcq: We have now added information about the choice of the method between MCA and LCA, and about the method WARD (regarding a following remark). We hope that it brings the missing information. If this information is not complete for you, could you precise us explicitly the requested information?*

*Lines 165-170: “This method was chosen instead of the creation of classes, possible with the Latent Class Analysis method or the Numerical Classification on the scores of MCA (Hierarchical Clustering on Principal Components). This choice was motivated by the wish to not put the producers in boxes but study their position on a gradient between potential extreme models identified along the dimension.”
Lines 153-156 : “The WARD method is a hierarchical agglomerative method* (Everitt et al., 2011)*. The principle of this kind of method is to put initially the n individuals in n groups and then to agglomerate the groups. The algorithm of WARD makes it in such a way that the gatherings induce the lowest decrease of R^2^ at each step.”*

**Overall, the authors have inadequately addressed the careful feedback of the reviewers and made inadequate improvements. Throughout the response to reviewers they reject several important critiques with no justification of their rejection.**

*A-C Dalcq: We recognized that we do not explain the choice of the method and we do not test its robustness with statistic treatments. We apologized for that. We missed this request. We realized this analysis at this time.*

**A number of specific points are highlighted below:**

**Line 79- How was that ensured (respondent producers were asked not to take into account their current farm when considering their IFF)?**

*A-C Dalcq: It is now precised in the Materials and methods section (Lines 131-134). Thanks for your remark.*

*Lines 131-134 : The question ‘Without taking into account your current farm, what is, according to you, the ideal future farm to ensure a revenue?” was proposed to the producers and they must choose between short propositions on seven items: 1) intensive or extensive production; 2)[…]”*

**Line 121- survey link not accessible without registering. Include in appendix?**

*A-C Dalcq: The appendix is realized and available in the new submission.*

**Line 146- What is WARD?**

*A-C Dalcq: We added sentences of explanation about the Ward method at lines 153-156. It is a hierarchical agglomerative method of Numerical classification.*

*Lines 153-156: “The WARD method is a hierarchical agglomerative method (19). The principle of this kind of method is to put initially the n individuals in n groups and then to agglomerate the groups. The algorithm of WARD makes it in such a way that the gatherings induce the lowest decrease of R^2^ at each step.”*

**Line 145- What are “particular characteristics” beyond no-opinion profiles?**

*A-C Dalcq: We replaced particular by “some” (Lines 151). We hope it makes it clearer.*

**Line 157- What?**

A-C Dalcq: *This sentence is a part of the method. As explained and precised before, we realized a MCA on the seven ideal future farm questions. We observed that the modalities “no-opinion” of the seven questions gathered and were positively related with the first dimension of the MCA. All the modalities reflecting an opinion were negatively related to the first dimension. Thus, the first dimension allowed to differentiate the producers with an opinion or not.*

*The modalities “intensive”, “global-market”, “specialized”, “standard quality milk”, “employed workforce” and “strongly based on new technologies” gathered and were positively related to the second dimension. The modalities “extensive”, “local-market”, “diversified”, “quality differentiated milk” and “lowly based on new technologies” gathered and were negatively related to the second dimension. The second dimension appeared to us as a gradient between the two extreme models of ideal future farm “Global-based intensive” and “Local-based extensive”.*

*The two dimensions explained almost 95% of the variability of the dataset. Therefore, the study of only these two dimensions appeared to us relevant. Then, we realized a numerical classification on the scores on the two dimensions of the MCA (statistic treatment equivalent to HCPC- Hierarchical Clustering on Principal Components). The first two groups created were the “no-opinion” producers and the producers “with an opinion”. This allowed us to exclude the “no-opinion” producers and to study the producers with an opinion thanks to the second dimension, this one had at its extremities the “Local-based extensive model” and the “Global-based intensive model”. But the producers with an opinion distributed themselves along this dimension. Thus, we decide to work with the second dimension, as a gradient of ideal future farm (Fig 5).*

GBI

LBE

Figure 5 Distribution of the producers with an opinion along the second MCA dimension (N = 207)

*Then we want to study the relationships between this ideal future farm gradient and the other information present in the survey.*

*The relationships between the ideal future farm gradient and the categorical variables of the survey were studied thanks to generalized linear models.*

*The gradient was the y, the variable to explain. The modalities of the categorical variables were the fixed effect of the generalized linear model, the factors explaining.*

*y = effect + residual*

*Where y was a vector contained the score on the ideal future farm gradient (the second dimension of MCA); effect was the qualitative variables of the survey. In other words, the model was :*

*Ideal future farm gradient = categorical variable + e*

*To study the relationships between the gradient and the quantitative variables of the survey, correlation coefficients and their level of significance were calculated.*

**Line 160- What are the quantitative variables?**

*A-C Dalcq: Quantitative is a statistical term defining a continuous numerical variable. Do you want that we use the term « numerical » ? But this term is less precise as it does not reflect the continuous dimension of the variable.*

**Table 1- What are the ns? Maybe a total figure? Percentages?**

*A-C Dalcq: Absolute frequencies named counts. We precised at lines 186 and 194: “Absolute frequencies (counts)”.*

**How many questions in each dimension?**

*A-C Dalcq: One question. We have now mentioned that in the Materials and methods section at Lines 187-188: “and of the answer to the question which corresponds to this corresponding characteristic for the current situation”.*

**Line 180- specifics of response rate still missing.**

*A-C Dalcq: The response rate is 6,1% (Line 200). Which supplementary indication do you need? We gave you numbers of farms with a dairy activity in Belgium and in European Union in this letter. We give also more information about the conditions where the survey was communicated to the producers to give you an idea of the way of proceeding. More information is given at lines 120-126.*

*Lines 120-126: “We communicated with Walloon dairy producers about the goals of the survey and its access broadly via all communication ways towards them : specialised press, agricultural internet websites, Unions and also advertisements through the milk payment letter which is sent to all the Walloon dairy producers once a month. The survey written in French can be viewed at the following internet link:* [*https://www.gembloux.ulg.ac.be/enquete/index.php/219425?lang=fr*](https://www.gembloux.ulg.ac.be/enquete/index.php/219425?lang=fr) *and its English translation is viewable in the Appendix“*

**Table 2- list questions?**

*We have added the question in the table (Line 223).*

| **Question**  Without taking into account your current farm, what is, according to you, the ideal future farm to ensure a revenue?” | **Proposition** | **Percentage (%)** |
| --- | --- | --- |
| Intensive *vs*. extensive | Intensive | 43 |
|  | Extensive | 30 |
|  | No opinion | 27 |
| Specialised *vs*. diversified | Specialised | 43 |
|  | Diversified | 47 |
|  | No opinion | 10 |
| Strongly *vs*. weakly based on new technologies | Strongly | 35 |
|  | Weakly | 41 |
|  | No opinion | 24 |
| Managed by an independent farmer *vs.* a group of managers | Independent farmer | 72 |
|  | Group of managers | 18 |
|  | No opinion | 10 |
| Family *vs*. employed workforce | Family | 87 |
|  | Employed | 5 |
|  | No opinion | 8 |
| Providing dairy production for local *vs*. global market | Global | 43 |
|  | Local | 32 |
|  | No opinion | 25 |
| Providing standard *vs*. differentiated quality dairy production | Standard | 38 |
|  | Differentiated quality | 45 |
|  | No opinion | 17 |

**Totals**

*A-C Dalcq: It is now mentioned at line 223.*

**Figure 1 define dimensions. What are the percentages in the axes labels?**

*A-C Dalcq: The percentages in the axes labels are the inertia but these ones underestimated the part of information explained by the dimensions. The corrected inertia values were calculated. This is explained in the Materials and methods section at lines 142-146: “For a MCA, the eigenvalue of the dimensions generated, named principal inertia, is a biased measure of the amount of information presented by a dimension (Palm, 2007). Corrected inertia rates were calculated, as described by Benzécri (Benzécri, 1979), to quantify the correct proportion of information of a dimension.”*

*The corrected values of inertia are presented at lines 248-250. The nature of the values are precised in the caption of the figure (lines 251-253).*

*“The percentage of principal inertia of the dimensions 1 and 2 of MCA were 16.75% and 12.38%, respectively (Fig 1). The value of corrected inertia for the two first dimensions reached 72.7% and 21.5% respectively, gathering almost 95% of the information.*

Fig 1. Representation of the modalities in the multiple correspondence analysis first factorial plan. Values of principal inertia reached 16.75% and 12.38%. Values of corrected inertia reached 72.7% and 21.5%.”

*The correction of Benzécri is made following this calculation:*

$$corrected inertia={(\frac{s}{s-1})}^{2}*\left( \mu_{k}-\frac{1}{s} \right)^{2} with \mu_{k}> \frac{1}{s}$$

*s = number of categorical variables in the MCA*

*µ_k_= eigenvalue of the dimension*

*(0.33508 and 0.2475, eigenvalues of respectively the first and second dimension of the present MCA)*

*The corrected inertia gives a better appreciation of the amount of information explained by each dimension (Benzécri, 1979), than the inertia automatically provided by the software.*

*We did not think necessary to precise this in the paper but we provide the reference where this calculation is presented.*

**Line 335- where is this figure reference from**

*A-C Dalcq : We did not provide a representation of the distribution of the producers along the second dimension, we did not think it was necessary as we provided the percentages and this figure would lengthen the paper. But following your request, we have now added this figure in the present manuscript (Lines 376-377).*

Fig 2. Distribution of the producers along the second dimension (the dotted line represents the mean score on the second dimension of the producers)(N = 207)

**Table 5-footnotes? Which is LBI and which GBI**

*A-C Dalcq : We were not sure about the goal of your question. We precised the function of the letters a and b. Concerning your request about the LBE and GBI, as explained with more details in the point 1/, the analysis is not done between groups LBE or GBI and the variables present in the survey but between an ideal future farm gradient and the variables present in the survey. The means presented in the tables 5, 7 and 8 are the mean value of this gradient for the different modalities of the categorical variables. We explained this at lines 390-395.*

*Lines 390-395: “Tables 5, 7 and 8 give the results of generalised linear models where the categorical variables were introduced separately as a fixed effect in the model. Significantly lower estimates of IFFg for a specific modality of the considered categorical variable depicts a tendency of producers desiring a LBE model to choose this modality, while significantly higher estimates of IFFg means a tendency of producers wanting a GBI model to choose this modality.”*

**What test is used here?**

*A-C Dalcq: Generalised linear models were used to study the level of significance of the differences between the means values of the gradient of the modalities of categorical variables: y was the ideal future farm gradient and the effect included in the model was the categorical variable. We explained this part of the method in the Materials and methods section at lines 178-184.*

*Lines 178-184: “For categorical variables, the scores of MCA dimensions were modelled using these variables as a fixed effect in a generalised linear model. Least squares means were estimated for the two-by-two comparisons using the Tukey test. The level of significance of those differences was assessed based on the P-value of the test. For quantitative variables, Pearson correlation coefficients were calculated between the scores of MCA dimensions and these variables. Their corresponding P-values were estimated to observe if the correlation values were significantly different from 0.”*

**Line 356 explain what they mean by introduced as fixed effect**

*A-C Dalcq: As explained in the point 1/ of this letter, the gradient was used as y (variable to be explained) of the generalized linear models and the fixed effect introduced in the generalized linear model was the categorical variables. See the previous answer related to the same topic for more details.*

**Line 620- What is SFI**

*A-C Dalcq : We precised this at line 661. “SFI = study, formation and information ». It is after the table. We have now added an asterisk to highlight the explanation of this abbreviation.*

**Reviewer #3: Thanks to the authors, ho made significant improvements to the paper. The full potential of the data is now revealed in the analysis. I particularly appreciate the improvements on the description of the « no-opinion » farmers, as suggested in my first review.**

*A-C Dalcq: Thank you for the interest given to this study.*

**Specific comments:**

**84: add references to « This change implied the disappearance of regulation of dairy supplies and caused volatility and decrease in the milk price »**

*A-C Dalcq: We added a reference and precised our purpose (Lines 83-84). Thanks for your remark.*

*Lines 83-84: “This change implied the disappearance of regulation of dairy supplies and was bringing uncertainty about the milk price (16). ~~caused volatility and decrease in the milk price.~~ “*

*Reference 16: Salou, T., H.M.G. van der Werf, F. Levert, A. Forslund, J. Hercule, and C. Le Mouël. 2017. Could EU dairy quota removal favour some dairy production systems over others? The case of French dairy production systems. Agric. Syst. 153:1–10. doi:10.1016/j.agsy.2017.01.004.*

**99-100: the question #2 is unclear. « What is the proportion of producers desiring the different IFF? » should be rephrased a bit maybe. The expression « the different IFF » will be vague for the readers. Is the IFF always different from the current farm ? And I guess « their IFF » is more thuitable thant « the IFF » as each respondent will provide a personal definition of their IFF.**

*A-C Dalcq: We understand your will to make this question clearer. We have replaced it by « How the dairy producers distribute themselves between IFF highlighted ?” (Lines 100-101, lines 365-366).*

**122: you do not answer to another reviewer’s comment, who wanted to know the response rate to the interview. To how many farmers was this survey submitted ? e.g. number of farmers buying the « specialised press », number of advertisements sent with the milk payment letter etc.**

*A-C Dalcq: We precised in the text that all the Walloon dairy producers received this payment letter: +/- 4,000 producers. Therefore, the 245 respondents correspond to 6,1% of the population, i.e. the response rate. We added information at lines 120-126 & 200-201. We do not know if the information number of farmers buying the « specialised press », number of advertisements sent with the milk payment letter are necessary in this paper but we added information to give an idea of the conditions in which the survey was communicated.*

*Lines 120-126: “We communicated with Walloon dairy producers about the goals of the survey and its access broadly via all communication ways towards them : specialised press, agricultural internet websites, Unions and also advertisements through the milk payment letter which is sent to all the Walloon dairy producers once a month. The survey written in French can be viewed at the following internet link:* [*https://www.gembloux.ulg.ac.be/enquete/index.php/219425?lang=fr*](https://www.gembloux.ulg.ac.be/enquete/index.php/219425?lang=fr) *and its English translation is viewable in the Appendix.*

*A total of 245 producers completed our survey between November 2014 and January 2015.”*

*Lines 200-201: “The sample set of 245 producers represented 6.1% of the dairy producers in Wallonia (about 4,000 dairy producers in 2015 and 3,500 in 2017* (STATBEL, 2019)*.”*

**240:253 : the description of the no-opinion farmers adds value to the data analysis. However I am not sure that the last sentence is useful. This is your personal interpretation, but the data do not permit to reveal it.**

*A-C Dalcq: Indeed, this sentence was deleted (Line 279). Thanks for your remark.*

**Tables 3 and 4: you have two columns, which are « complete sample » and « no-opinion farmers ». A third column which reflects the sample excluding the no-opinion farmers will permit the reader to compare the no-opinion ones with the others.**

*A-C Dalcq: Indeed, this column was added (Lines 280, 283). And, as asked by reviewer#2, we realized (1) generalized linear models to compare the means of the quantitative variables between the no-opinion producers and producers with an opinion and (2) tests of proportion to compare the proportion of each modalities of the categorical variables between the no-opinion producers and producers with an opinion. We added description of these statistical treatments in the Materials and Methods section (Lines 157-160).*

**333 : as already said, I think this title is not well written and could be more explicit.**

*A-C Dalcq: It was changed at lines 365-366. See also the answer to your previous comment related to the same topic.*

**350 : pigeonholes. Could you be more precise?**

*A-C Dalcq: As it caused doubt in your comprehension of this idea, we replaced by boxes (Line 384).*

- *Put people (here dairy producers) in boxes.*

*It was used to explain the fact to put a label of someone. But in this paper, we want to nuance the position of the producer regarding its ideal future farm.*
